# NNB-Type Tridentate Boryl Ligands Enabling a Highly Active Iridium Catalyst for C–H Borylation

**DOI:** 10.3390/molecules24071434

**Published:** 2019-04-11

**Authors:** Siyi Ding, Linghua Wang, Zongcheng Miao, Pengfei Li

**Affiliations:** 1 Key Laboratory of Organic Polymer Photoelectric Materials, School of Science, Xijing University, Xi’an 710123, China; dingsiyi2009@163.com; 2 Center for Organic Chemistry, Frontier Institute of Science and Technology, Xi’an Jiaotong University, Xi’an 710054, China; wanglinghua1036@126.com

**Keywords:** boryl ligand, iridium-catalyzed, C–H borylation reaction

## Abstract

Boryl ligands play a very important role in catalysis because of their very high electron-donating property. In this paper, NNB-type boryl anions were designed as tridentate ligands to promote aryl C–H borylation. In combination with [IrCl(COD)]_2_, they generate a highly active catalyst for a broad range of (hetero)arene substrates, including highly electron-rich and/or sterically hindered ones. This work provides a new NNB-type tridentate boryl ligand to support homogeneous organometallic catalysis.

## 1. Introduction

**Scheme 1:**
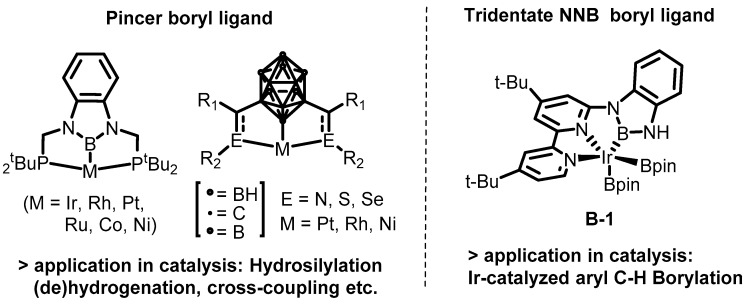
Pincer-type tridentate boryl ligand and proposed structures for the real catalyst in the iridium-catalyzed C–H borylation using the NNB ligand.

In many types of stoichiometric and catalytic organometallic reactions, transition metals together with boryl ligands play a very important role as key intermediates [1,2,3,4,5,6,7,8]. Among these reactions, the *sp^2^* boryl anion can be transferred to the target product from metal complexes, which we usually call a “reactive” ligand; the boron atom acting as a “reactive” boryl anion ligand has much stronger capacity for donating σ-electrons compared with C, N, and O atoms [9,10]. In 2009, Yamashita and Nozaki successfully synthesized the tridentate boryl anion PBP-type ligand [11] and its iridium complexes for the first time. Since then, the chemistry of pincer boryl ligands and their transition-metal complexes have rapidly advanced in recent years. In the same year, Mirkin prepared the SeBSe–Pd and SBS–Pd complexes using a carborane-based pincer ligand [12] for the first time. Unfortunately, the XBX-type ligand (X = P, S, Se) was not suitable for the transition metal-catalyzed reactions until the formation of the first carborane-based chiral NBN pincer-type ligand in 2011 [13]. Meanwhile, the Carbox–Rh–NBN complexes were examined as chiral catalysts for the asymmetric conjugate reduction of α, β–unsaturated esters, and reductive aldol reaction. Yamashita synthesized a platinum–PBP complex in 2013 that was very reactive in the metal-catalyzed hydrosilylation of olefins [14]. During 2013 and 2014, Peters and Lin published their work on the preparation and application of PBP pincer-type tridentate ligands and related cobalt complexes [15,16]. In addition, the combination of *o-*carborane and *m-*carborane-based NBN pincer-type ligands and palladium catalysts can serve as good catalyst precursors in Suzuki coupling reactions with low amounts of catalyst loadings (Scheme 1).

## 2. Results

**Scheme 2:**
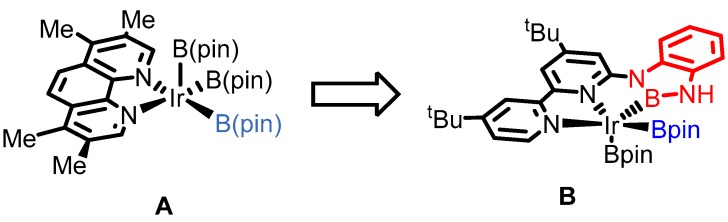
Rational design for the real catalyst in iridium-catalyzed C–H borylation using the NNB ligand.

Arylboron compounds are very useful building blocks in organic chemistry [17,18,19,20,21,22]; the most commonly used method to form the C–B bond is iridium-catalyzed C–H borylation [23,24,25,26,27,28,29,30,31,32,33,34,35,36,37,38]. The well-known catalyst system for this transformation is the combination of an iridium catalyst and a 2,2’-bipyridine-type ligand, such as 4,4′-di-tert-butyl bipyridine (dtbpy), 1,10-phenanthroline (1,10-phen), or 3,4,7,8-tetramethyl-1,10-phenanthroline (tmphen) [39,40,41,42]. Previous studies demonstrated that Compound **A** shown in Scheme 2 was the key intermediate in the iridium-catalyzed C–H borylation reaction. There are three boryl anion ligands in Compound **A**, but only one can be transferred to the arene ring, which can be referred as a “reactive” ligand. Herein, we present our results in developing a newly tridentate boryl anion NNB-type ligand that was synthesized through the recombination of the bidentate N ligand and a boryl anion. As expected, it can be applied in highly active Ir-catalyzed C–H borylation (Scheme 2).

**Scheme 3:**
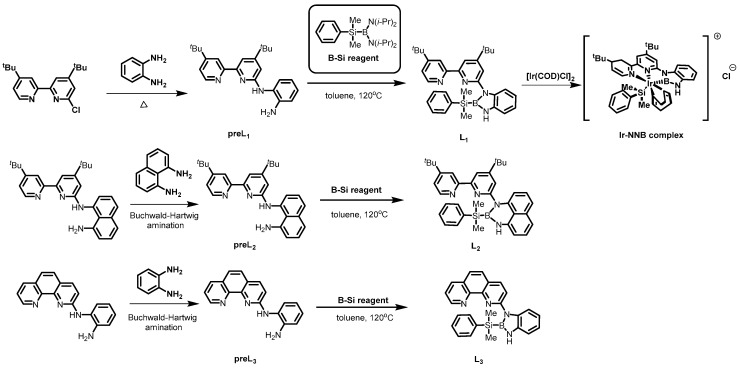
Preparation of the NNB ligand and the oxidative addition with [Ir(COD)Cl]_2._ and NNB Ligand 1.

The synthetic route to preligand **NNB-Ln** is shown in Scheme 3. For preligand **NNB-L1**, heating a neat mixture of 2-chloro-4,4’-di-tert-butyl bipyridine with a 10.0 equivalent of o-phenylenediamine [43,44,45] gave **preL_1_** in a 48% isolated yield. The treatment of **preL_1_** with a 1.05 equivalent of B–Si reagent [46,47,48,49,50] at 120 °C in toluene cleanly gave the new **NNB-L_1_** in excellent yield. As for the other two preligands, **preL_2_** and **preL_3_** were synthesized through Buchwald–Hartwig amination [51,52,53] in a Pd/BINAP catalytic system in moderate yield; the second step is identical to that previously mentioned. When a solution of **L_1_** and a 0.5 equivalent of [Ir(COD)Cl]_2_ in hexane was heated at 70 °C for 12 h, an **Ir-NNB complex** was formed in a 90% isolated yield after hexane purification. This complex showed a great catalytic effect in the iridium-catalyst C–H borylation reaction (Table 1, entry 5).

We chose 1,3-dimethoxybenzene as the starting material because of its electron-rich property; it was used in iridium-catalyzed aryl C–H borylation to test the effectiveness of the NNB ligand. After repeated attempts, we found that a mixture of precatalyst [Ir(COD)Cl]_2_ (1 mol%), **NNB-L_1_** (2 mol%), B_2_pin_2_ (1.0 equivalent), and 1,3-dimethoxybenzene was heated at 100 °C in cyclopentyl methyl ether (CPME) for 3 h, leading to quantitatively produce the borylated product. Compared with classical ligands such as dtbpy, 1,10-phen, and tmphen in standard condition, the NNB-type ligands showed excellent catalytic effect (Table 1, entries 1 to 4). To further test the effectiveness of the NNB-type ligands in Ir-catalyzed C–H borylation, **L_2_** and **L_3_** performed slightly worse (Table 1, entries 15 and 16). Changing the reaction temperature was not helpful for borylation (Table 1, entries 13 and 14). The reaction was sensitive to the dosage of the boron reagents (Table 1, entries 4 to 9). The borylated yield could reach 81% in one hour, and then slowly rise to the maximum in three hours (Table 1, entries 10 to 12). An attempt to run the reaction in air atmosphere resulted in no target product. Finally, the reaction could be conducted on the 1.0-mmol and 5.0-mmol scales, producing the target product in 85% and 75% isolated yields, respectively (for more details, see the Supplementary Material).

**Scheme 4:**
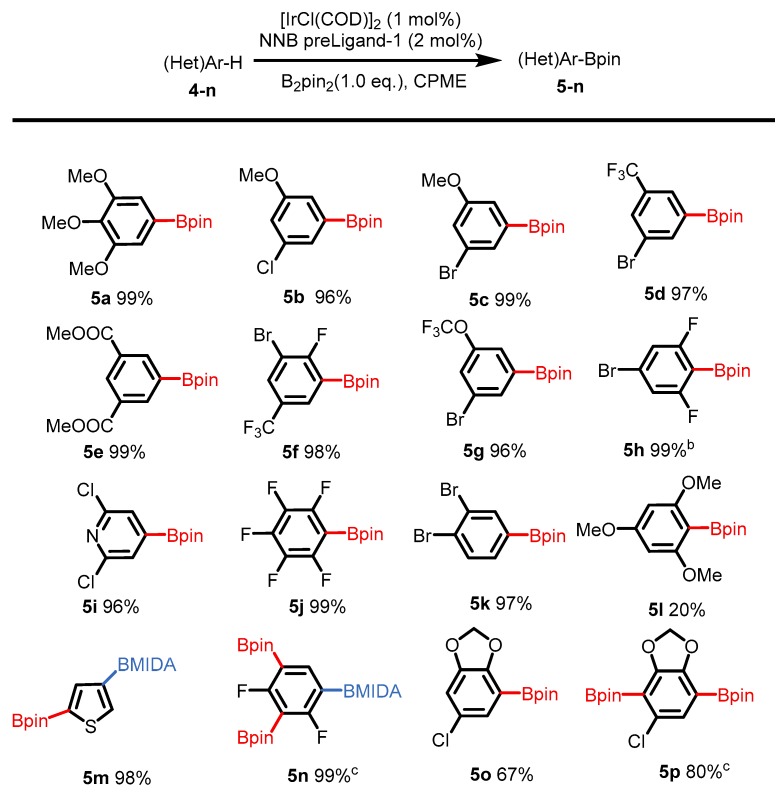
Substrate scope of C–H borylation using NNB-L_1_^a^. ^a^ Yield of the isolated product. ^b^ Reaction was conducted at 140 °C for 12 h. ^c^ 2.0 equivalent of B_2_pin_2_ was used in the standard condition.

Having identified optimal reaction conditions, we explored the substrate scope for this direct C–H borylation, as shown in Scheme 4. Various arenes with electron-donating or electron-withdrawing substituents are good substrates, cleanly forming the desired borylated products in moderate to high yields. The borylation reaction was under the control of steric hindrance. For the 1,3-disubstituted arenes, various functional groups at the 3-position on the arene ring, such as halogen, ether groups, and alkoxyl groups, were well tolerated, which can afford the corresponding borylated products at the 5-position. The 2,6-dichloropyridine was also found to successfully participate in the current reaction. The borylation of 5-chloro-1,3-benzodioxole with a 1.0 equivalent of B_2_pin_2_ produced a mixture of monoborylated and diborylated products. The isolated yield of the monoborylated product was 67%, but when we used a 2.0 equivalent of B_2_pin_2_ instead, the isolated yield of the diborylated product could be 80%. For a small atom such as fluorine, both of the two ortho positions could be borylated in the standard condition to give a mixture of monoborylated and diborylated products that are not easy to separate and purify; only by increasing the amount of the boron reagent could we get the diborylated products in good yield. Aryl or heteroaryl MIDA (N-methyliminodiacetic acid) boronates also served as viable substrates, producing the diborylated products in an almost quantitative yield. We could conveniently and efficiently obtain the arenes with different boron groups in a moderate yield, which could be used in the selective Suzuki–Miyaura cross-coupling reactions.

## 3. Discussion

**Scheme 5:**
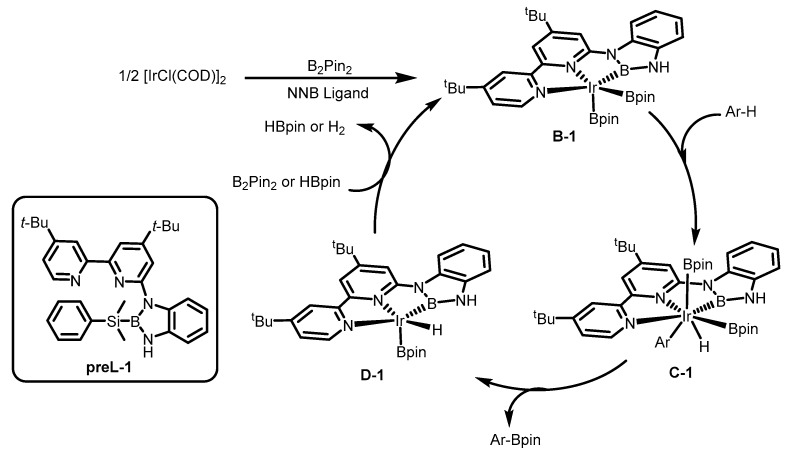
Proposed mechanism.

On the basis of the experiment results, we proposed a catalytic cycle for the present Ir/pre-NNB ligand catalytic borylation reaction, as depicted in Scheme 5. Taking the NNB L_1_ ligand as an example, the iridium catalyst reacted with the NNB L_1_ ligand and B_2_pin_2_ to form the real reactive intermediate **B-1**; then, **B-1** went through oxidative addition with the aryl C–H bond to form the five valent iridium intermediate **C-1**. Then, a reductive elimination reaction occurred to give the borylated product and Ir(III) intermediate **D-1**; the Ir–H bond in the intermediate **D-1** could exchange with B_2_pin_2_ or HBpin to form the intermediate **B-1**, which was used to restart the catalytic cycle.

## 4. Materials and Methods

### 4.1. Methods and Material

#### 4.1.1. General Information

Unless otherwise noted, all the reactions were carried out in a flame-dried, sealed Schlenk reaction tube under an atmosphere of nitrogen. Analytical thin-layer chromatography (TLC) was performed on glass plates coated with 0.25-mm 230 to 400-mesh silica gel containing a fluorescent indicator. Preparative thin-layer chromatography (PTLC) was performed on pre-coated, glass-backed GF254 silica gel plates. Visualization was accomplished by exposure to a UV lamp. All the products in this article are compatible with standard silica gel chromatography. Column chromatography was performed on silica gel (200 to 300 mesh) using standard methods.

#### 4.1.2. Structural analysis

NMR spectra were measured on a nuclear magnetic resonance apparatus (Avance III HD 400M, Bruker, Germany) and chemical shifts (δ) are reported in parts per million (ppm). ^1^H-NMR spectra were recorded at 400 MHz in NMR solvents and referenced internally to the corresponding solvent resonance, and ^13^C-NMR spectra were recorded at 100 MHz and also referenced to the corresponding solvent resonance. Carbons bearing boron substituents were generally not observed due to quadrupolar relaxation. Coupling constants are reported in Hz with multiplicities denoted as s (singlet), d (doublet), t (triplet), q (quartet), m (multiplet), and br (broad). High-resolution mass spectra (HRMS) were acquired with an ESI source.

#### 4.1.3. Materials

Commercial reagents and solvent were purchased from J&K Chemical Ltd. (Shanghai, China), Energy Chemical Co. (Shanghai, China), Sigma-Aldrich (Shanghai, China), Alfa Aesar (Beijing, China), Acros Organics (Shanghai, China), Strem Chemicals (Shanghai, China), TCI (Shanghai, China) and used as received, unless otherwise stated.

### 4.2. The Preparation of New NNB-Type Tridentate Boryl Anion Ligand

*4,4′-Di-tert-butyl-[2,2′-bipyridine] 1-oxide.* A solution of dtbpy (1.34 g, 5.0 mmol, 1.0 eq.) in CHCl_3_ was stirred at 0 °C for 35 min; then, a solution of *m*-chloroperbenzoic acid (1.03 g, 6.0 mmol, 1.2 eq.) in CHCl_3_ was added dropwise to the mixture slowly, and the mixture was stirred at room temperature for 20 h. Then, the solution was washed with 5% aqueous Na_2_CO_3_ solution, dried over anhydrous Na_2_SO_4_, and concentrated. The Na_2_CO_3_ washings were extracted with CHCl_3_. The extract was dried over anhydrous Na_2_SO_4_ and concentrated. Each residue oil was put together and then purified by column chromatography using a PE:EA ratio of 10:1 to get the target product as a light yellow solid, (1.08 g, 3.8 mmol, yield = 76%). (The spectral data were in accordance with literature [54]). ^1^H-NMR (400 MHz, Acetone-*d*_6_) δ 9.00 (dd, *J* = 0.7, 2.0 Hz, 1H), 8.50 (dd, *J* = 0.7, 5.2 Hz, 1H), 8.10–8.08 (m, 2H), 7.35–7.32 (m, 2H), 1.25 (s, 9H), 1.24 (s, 9H); ^13^C NMR (400 MHz, Acetone-*d*_6_) δ 159.7, 150.2, 149.2, 148.7, 145.8. 140.0, 124.3, 122.9, 122.2, 121.1, 34.6, 34.3.

*4,4′-Di-tert-butyl-6-chloro-2,2′-bipyridine*. A mixture of 4,4′-di-tert-butyl-[2,2′-bipyridine] 1-oxide (568.4 mg, 2 mmol, 1.0 eq.) and phosphoryl trichloride (20 mmol, 10.0 eq.) was refluxed for 4 h. Phosphoryl chloride was distilled out, and the residue was diluted with ethyl acetate. The organic layer was washed with saturated aqueous sodium bicarbonate solution and brine, and concentrated under reduced pressure. Then, it was purified by column chromatography on silica gel (PE:EA 10:1) to get the target product as a light yellow solid, (332.4 mg, 1.1 mmol, yield = 56%). The spectral data were in accordance with the literature [54]. ^1^H-NMR (400 MHz, Acetone-*d*_6_) δ 8.47 (dd, *J* = 0.6, 5.2 Hz, 1H), 8.37 (d, *J* = 1.6 Hz, 1H), 8.45 (dd, *J* = 0.6, 2.0 Hz, 1H), 7.37–7.35 (m, 2H), 1.27 (s, 9H), 1.26 (s, 9H); ^13^C-NMR (400 MHz, Acetone-*d*_6_) δ 164.8, 161.0, 156.8, 154.5, 150.9, 149.4, 121.5, 121.1, 117.6, 116.7, 35.1, 34.7.

*1,10-Phenanthroline 1-oxide*. 1,10-phenanthroline (4.7 g, 26 mmol), concentrated acetic acid (30 mL), water (2 mL), and 30% hydrogen peroxide (3.2 mL) were stirred at 70 °C for 3 h. A second crop of 30% hydrogen peroxide (3.2 mL) was added, and stirring was maintained at 70 °C for three more hours. After cooling to room temperature (RT), a last batch of 30% hydrogen peroxide (2 mL) was added, and the resulting mixture was stirred for 12 h. Evaporation under vacuum reduced the volume to 10 mL; then, fresh water (35 mL) was added and the mixture was concentrated to 10 mL, cooled to 10 ^o^C, and neutralized with potassium carbonate (50 g). The resulting yellow-brown solid was isolated and extracted with hot chloroform under reflux for 12 h (soxhlet). The organic layer was dried over magnesium sulfate and charcoal, filtered, and evaporated to dryness to give 1,10-phenanthroline-*N*-oxide (3.75 g, 19.1 mmol, yield = 74%). The spectral data were in accordance with the literature [55]. ^1^H-NMR (400 MHz, CDCl_3_) δ 9.31 (dd, *J* = 1.8, 4.3 Hz, 1H), 8.73 (dd, *J* = 1.2, 6.3 Hz, 1H), 8.23 (dd, *J* = 1.8, 8.0 Hz, 1H), 7.80 (d, *J* = 8.8 Hz, 1H), 7.74 (d, *J* = 8.9 Hz, 1H), 7.74 (d, *J* = 8.0 Hz, 1H), 7.66 (dd, *J* = 4.4, 8.1 Hz, 1H), 7.45 (dd, *J* = 0.3, 8.1 Hz, 1H); ^13^C-NMR (400 MHz, CDCl_3_) δ 150.0, 142.7, 140.8, 138.5, 135.8, 133.3, 129.1, 128.9, 126.5, 124.3, 123.1, 122.8.

*2-Chloro-1,10-phenanthroline.* To the mixture of 1,10-phenanthroline-N-oxide (196.1 mg, 1 mmol, 1.0 eq.), sodium chloride in anhydrous DMF, a neat POCl_3_ (3 mmol, 3.0 eq.) was added slowly after cooling to 0 °C. Then, the mixture was heated to 100 °C for 6 hours. After cooling to room temperature, water was added, and the mixture was quenched with saturated ammonium chloride (aqueous) and saturated with NaCl (20 mmol, 20.0 eq.), solids were filtered, and the solution was extracted with chloroform. The combined extracts were washed with brine, dried over MgSO_4_, and evaporated, and then purified by column chromatography using eluent EA/PE (3:2) to get the target product as a light yellow solid, (94.2 mg, 0.44 mmol, yield = 44%). The spectral data were in accordance with the literature [55]. ^1^H-NMR (400 MHz, CDCl_3_) δ 9.12 (dd, *J* = 1.7, 4.1 Hz, 1H), 8.57 (d, *J* = 9.4 Hz, 1H), 8.53 (dd, *J* = 1.7, 8.0 Hz, 1H), 8.07–8.03 (m, 2H), 7.84 (d, *J* = 8.4 Hz, 1H), 7.81 (dd, *J* = 4.3, 8.2 Hz, 1H); ^13^C-NMR (400 MHz, CDCl_3_) δ 151.3, 150.7, 146.0, 145.0, 138.7, 136.0, 129.0, 127.2, 126.9, 125.7, 124.2, 123.4.

*N1-(4,4′-di-tert-butyl-[2,2′-bipyridin]-6-yl)benzene-1,2-diamine(**pre L_1_**).* Benzene-1,2-diamine (1.08g, 10 mmol, 10.0 eq.) was mixed with 4,4′-di-tert-butyl-6-chloro-2,2′-bipyridine (302,2 mg, 1 mmol, 1.0 eq.) and heated at 160 °C for 10 h. After cooling down to room temperature, water (3.0 mL) and acetone (3.0 mL) were added to fully dissolve the solid mixture. The pH value of the resulting solution was adjusted to 10 by adding Na_2_CO_3_. Then, the mixture was extracted with ethyl acetate three times, and the combined organic layers were washed with brine, dried over anhydrous Na_2_SO_4_, and concentrated under reduced pressure. The residue was purified by silica gel chromatography (EtOAc/PE = 1:3 *v*/*v*) to give the target product as a light yellow solid (187.1 mg, 50% yield).^1^H-NMR (400 MHz, CDCl_3_) δ 8.58 (d, *J* = 5.2 Hz, 1H), 8.33 (d, *J* = 1.4 Hz, 1H), 7.83 (d, *J* = 1.4 Hz, 1H), 7.32 (dd, *J* = 1.2, 7.8 Hz, 1H), 7.28 (dd, *J* = 2.0, 5.2 Hz, 1H), 7.07 (td, *J* = 6.0, 2.0 Hz, 1H), 6.85 (dd, *J* = 1.3, 7.9 Hz, 1H), 6.79 (td, *J* = 7.6, 1.4 Hz, 1H), 6.56 (d, *J* = 0.9 Hz, 1H), 1.38 (s, 9H), 1.30 (s, 9H); ^13^C-NMR (400 MHz, CDCl_3_) δ 160.8, 156.8, 149.1, 142.1, 126.3, 125.6, 120.6, 119.0, 118.2, 116.5, 110.1, 104.9, 35.1, 35.0, 30.6, 30.6; HRMS (ESI^+^): Calculated for C_24_H_30_N_4_ [M + H] 375.2543, found 375.2533.

*N1-(4,4′-di-tert-butyl-[2,2′-bipyridin]-6-yl)naphthalene-1,8-diamine* (**pre L_2_**). Naphthalene-1,8-diamine (1.58 g, 10 mmol, 10.0 eq.) was mixed with 4,4′-di-tert-butyl-6-chloro-2,2′-bipyridine (302.2 mg, 1 mmol, 1.0 eq.), Pd_2_(dba)_3_ (18.3 mg, 2.0 mol%), BINAP (24.9 mg, 4.0 mol%), and *t*BuONa (19.2 mg, 1.2 mmol, 1.2 eq.) under nitrogen atmosphere, and then heated to 110 °C for 10 hours. After cooling down to room temperature, water (3.0 mL) was added. Then, the mixture was filtered through a pad of celite, using ethyl acetate to wash the filtrate; afterwards, the combined organic layers were washed with brine, dried over anhydrous Na_2_SO_4_, and concentrated under reduced pressure. The residue was purified by silica gel chromatography (CH_2_Cl_2_/MeOH = 20:1 *v*/*v*) to give the target product as a light pink solid (263.0 mg, 62% yield). ^1^H-NMR (400 MHz, CDCl_3_) δ 8.59 (d, *J* = 5.2 Hz, 1H), 8.36 (s, 1H), 7.91 (s, 1H), 7.66 (d, *J* = 7.4 Hz, 1H), 7.59 (d, *J* = 8.1 Hz, 1H), 7.55 (s, 1H), 7.36 (m, 1H), 7.29 (m, 2H), 6.69 (m, 2H), 1.38 (s, 9H), 1.27 (s, 9H); ^13^C NMR (400 MHz, CDCl_3_) δ 162.9, 160.5, 157.4, 156.7, 154.6, 149.0, 144.0, 138.0, 136.9, 126.3, 125.8, 125.4, 120.6, 120.3, 120.0, 119.2, 118.4, 112.2, 110.7, 106.5, 34.7, 30.3. HRMS (ESI^+^): Calculated for C_28_H_32_N_4_ [M + H] 425.2699, found 425.2700.

*N1-(1,10-phenanthrolin-2-yl)benzene-1,2-diamine (**pre L_3_**).* Benzene-1,2-diamine (1.08 g, 10 mmol, 10.0 eq.) was mixed with 2-chloro-1,10-phenanthroline (214.0 mg, 1 mmol, 1.0 eq.), Pd_2_(dba)_3_ (18.3 mg, 2.0 mol%), BINAP (24.9 mg, 4.0 mol%), and *t*BuONa (19.2 mg, 1.2 mmol, 1.2 eq.) under nitrogen atmosphere, and then heated to 110 °C for 10 h. After cooling down to room temperature, water (3.0 mL) was added. Then, the mixture was filtered through a pad of celite, using ethyl acetate to wash the filtrate; afterwards, the combined organic layers were washed with brine, dried over anhydrous Na_2_SO_4_, and concentrated under reduced pressure. The residue was purified by silica gel chromatography (CH_2_Cl_2_/MeOH = 20:1 *v*/*v*) to give the target product as a light pink solid (221.9 mg, 66% yield). ^1^H-NMR (400 MHz, CDCl_3_) δ 9.12 (d, *J* = 3.9 Hz, 1H), 8.21 (d, *J* = 8.0 Hz, 1H), 7.98 (d, *J* = 8.8 Hz, 1H), 7.66 (d, *J* = 8.6 Hz, 1H), 7.58 (m, 1H), 7.54 (d, *J* = 8.8 Hz, 1H), 7.26 (s, 1H), 7.23 (d, *J* = 7.7 Hz, 1H), 7.14 (m, 1H), 6.82 (s, 3H); ^13^C-NMR (400 MHz, CDCl_3_) δ 157.4, 149.6, 145.6, 144.9, 143.7, 138.7, 136.1, 129.4, 129.3, 127.9, 126.5, 124.7, 123.2, 122.5, 121.9, 118.7, 115.9, 109.8. HRMS (ESI^+^): Calculated for C_18_H_14_N_4_ [M + H] 287.1297, found 287.1289.

*1-Chloro-N,N,N′,N′-tetraisopropylboranediamine*. Trichloroborane (1.17 g, 10 mmol, 1.0 eq.), diisopropylamine (4.55 g, 45 mmol, 4.5 eq.), and toluene were added in flask at room temperature; a precipitate formed, and the temperature of the reaction was raised to above 40 °C. Then, the mixture was heated under reflux for 8 h. After cooling, the precipitate was rapidly filtered off, washed with 2*10 mL of cyclohexane, and concentrated; then, distillation of the filtrate yielded the pure target product as a colorless oil (~2.2 g). The spectral data were in accordance with the literature [45].

*1-(Dimethyl(phenyl)silyl)-N,N,N′,N′-tetraisopropylboranediamine*

Step I: Chlorodimethylphenylsilane was added to a suspension of Li in THF at room temperature. The mixture was stirred overnight to afford a solution of dimethylphenyl(silyl) lithium (~ 0.8 M).

Step II: Dimethylphenyl (silyl) lithium was added dropwise to 1-chloro-*N,N,N′,N′*-tetra-isopropyl-boranediamine (2.2 g, 8.9 mmol) in hexane at room temperature, and the mixture was stirred until the color changed from brown to white. Afterwards, the produced precipitate was filtered off under argon atmosphere and washed with dried THF. Then, the filtrate was evaporated and distilled to afford the target product at approximately 110 to 120 °C as a colorless oil (2.0 g, 65% yield). The spectral data were in accordance with the literature [45].

*1-(4,4′-Di-tert-butyl-[2,2′-bipyridin]-6-yl)-2-(dimethyl(phenyl)silyl)-2,3-dihydro-1H-benzo[d]*[1,3,2]*diazaborole (**L1**).* A mixture of *N*1-(4,4′-di-tert-butyl-[2,2′-bipyridin]-6-yl)benzene-1,2-diamine (**pre L*_1_***) (93.6 mg, 0.25 mmol, 1.0 eq.) and 1-(dimethyl(phenyl)silyl)-*N,N,N′,N′*-tetraisopropylboranediamine (90.0 mg, 0.26 mmol, 1.05 eq.) in anhydrous toluene was heated to 120 °C for 10 h. After cooling to room temperature, the crude product was obtained until the solvent was evaporated under vacuum, and the pure target product was obtained after washing with anhydrous hexane (115.3 mg, 89% yield). ^1^H-NMR (400 MHz, CDCl_3_) δ 8.73 (d, *J* = 5.7 Hz, 1H), 8.49 (d, *J* = 1.4 Hz, 1H), 8.33 (d, *J* = 1.6 Hz, 1H), 7.67 (d, *J* = 9.1 Hz, 1H), 7.52–7.49 (m, 2H), 7.32–7.27 (m, 4H), 7.25 (s, 1H), 7.05 (m, 1H), 7.02 (m, 3h), 1.36 (s, 9H), 1.28 (s, 9H), 0.34 (s, 6H); ^13^C-NMR (400 MHz, CDCl_3_) δ 165.7, 163.1, 158.3, 157.9, 156.8, 151.2, 142.3, 139.2, 138.9, 136.4, 130.8, 130.1, 123.0, 122.7, 121.9, 120.9, 118.4, 117.7, 114.2, 113.7, 37.4, 37.2, 32.8, 32.7, 2.2; HRMS (ESI^+^): Calculated for C_32_H_39_BN_4_Si [M + H] 519.3109, found 519.3113.

*1-(4,4′-Di-tert-butyl-[2,2′-bipyridin]-6-yl)-2-(dimethyl(phenyl)silyl)-2,3-dihydro-1H-naphtho [1,8-de][1,3,2]diazaborinine **(L2**)*. N1-(4,4′-di-tert-butyl-[2,2′-bipyridin]-6-yl)naphthalene-1,8-diamine (**pre L_2_**) (106.1 mg, 0.25 mmol, 1.0 eq.) and 1-(dimethyl(phenyl)silyl)-N,N,N′,N′-tetraisopropylboranediamine (90.0 mg, 0.26 mmol, 1.05 eq.) in anhydrous toluene was heated to 120 °C for 10 h. After cooling to room temperature, the crude product was obtained until the solvent was evaporated under vacuum, and the pure target product was obtained after washing with anhydrous hexane (109.4 mg, 77% yield). ^1^H-NMR (400 MHz, CDCl_3_) δ 8.50 (d, *J* = 5.2 Hz, 1H), 8.34 (d, *J* = 12.4 Hz, 2H), 7.30–7.29 (m, 2H), 7.20–7.16 (m, 4H), 7.10 (s, 1H), 7.01–6.86 (m, 4H), 6.15 (d, *J* = 7.1 Hz, 1H), 5.85 (s, 1H), 5.70 (d, *J* = 7.5 Hz, 1H), 1.22 (s, 9H), 1.24 (s, 9H), 0.00 (s, 3H), –0.30 (s, 3H); ^13^C-NMR (400 MHz, CDCl_3_) δ 164.5, 161.0, 157.6, 156.2, 155.7, 149.0, 143.2, 139.5, 139.4, 136.3, 134.0, 129.1, 128.6, 128.3, 128.0, 127.4, 127.4, 121.1, 121.0, 120.5, 118.9, 118.5, 118.2, 117.3, 106.0, 105.6, 35.2, 35.1, 30.7, 30.6, 22.7, 14.2. HRMS (ESI^+^): Calculated for C_36_H_41_BN_4_Si [M + H] 569.3272, found 569.3269.

*2-(2-(dimethyl(phenyl)silyl)-2,3-dihydro-1H-benzo[d]*[1,3,2]*diazaborol-1-yl)-1,10-phenanthroline (**L3**).* A mixture of N1-(1,10-phenanthrolin-2-yl)benzene-1,2-diamine (**pre L*_3_***) (84.0 mg, 0.25 mmol, 1.0 eq.) and 1-(dimethyl(phenyl)silyl)*-N,N,N′,N′*-tetraisopropylboranediamine (90.0 mg, 0.26 mmol, 1.05 eq.) in anhydrous toluene was heated to 120 °C for 10 h. After cooling to room temperature, the crude product was obtained until the solvent was evaporated under vacuum, and the pure target product was obtained after washing with anhydrous hexane (86.1 mg, 80% yield). ^1^H-NMR (400 MHz, CDCl_3_) δ 9.19 (dd, *J* = 1.6, 4.2 Hz, 1H), 8.25 (dd, *J* = 1.6, 8.0 Hz, 1H), 8.20 (d, *J* = 8.4 Hz, 1H), 7.78 (d, *J* = 4.1 Hz, 2H), 7.62 (d, *J* = 8.3 Hz, 2H), 7.46 (dd, *J* = 1.8, 7.5 Hz, 2H), 7.25–7.18 (m, 5H), 7.05–7.04 (m, 2H), 6.97 (s, 1H); ^13^C-NMR (400 MHz, CDCl_3_) δ 156.9, 152.7, 148.4, 148.3, 142.8, 140.4, 139.6, 139.0, 138.2, 136.5, 131.5, 130.7, 130.1, 129.0, 128.5, 128.1, 125.5, 123.2, 122.5, 122.2, 114.2, 113.8, 25.1, 16.6. HRMS (ESI^+^): Calculated for C_26_H_23_BN_4_Si [M + H] 431.4010, found 431.4018.

### 4.3. The Preparation of Iridium-NNB Ligand Complex

In a nitrogen-filled glove box, [Ir(cod)Cl]_2_ (6.7 mg, 0.01 mmol, 1.0 eq.) and NNB-L_1_ (10.4 mg, 0.02 mmol, 2.0 eq.) were added to a 10-mL flask; then, n-hexane (1.0 mL) was added as solvent. The flask was removed from the glove box and stirred at 70 °C for 12 h to afford a yellow suspension. Then, the solvent was removed from the suspension by syringe and the residue was washed with dry THF. Then, it was vacuumed and a 15.9-mg (90% yield) light yellow solid was obtained. ^1^H-NMR (400 MHz, CDCl_3_) δ 10.01 (s, 1H), 8.86 (d, *J* = 5.9 Hz, 1H), 8.19 (s, 1H), 7.79 (d, *J* = 7.7 Hz, 1H), 7.71 (s, 1H), 7.64 (s, 1H), 7.54 (d, *J* = 5.2 Hz, 1H), 7.45 (d, *J* = 7.8 Hz, 1H), 7.17 (t, *J* = 7.5 Hz, 1H), 7.03 (t, *J* = 7.5 Hz, 1H), 6.89 (t, *J* = 5.9 Hz, 1H), 6.66 (t, *J* = 7.3 Hz, 2H), 6.33 (d, *J* = 7.2 Hz, 2H), 5.30 (m, 1H), 4.37 (m, 1H), 4.05 (m, 1H), 3.85 (m, 1H), 3.56 (m, 1H), 2.94 (m, 1H), 2.49 (m, 1H), 2.28 (m, 1H), 1.84 (m, 3H), 1.54 (s, 9H), 1.53 (s, 9H), 0.10 (s, 3H), 0.13(s, 3H). ^13^C-NMR (100 MHz, CDCl_3_) δ 166.7, 164.9, 157.7, 157.0, 154.0, 153.4, 142.9, 142.3, 132.8, 128.0, 127.9, 125.7, 124.1, 121.9, 119.9, 114.9, 113.4, 112.6, 111.9, 109.2, 108.8, 73.8, 65.2, 37.1, 36.8, 32.7, 31.9, 31.7, 30.8, 26.1, 23.8, 15.2, 1.1, 0.4.

### 4.4. General Procedure for Iridium-Catalyzed arene C–H borylation Using NNB-type Ligand 

B_2_pin_2_, [IrCl(COD)]_2_ (1.0 mol%), preligand 1 (2.0 mol%), and (hetero)arene (0.2 mmol, if solid) were placed in a dried Schlenk flask (15 mL in volume) equipped with a stirring bar. After evacuating and refilling the flask with dry nitrogen three times, (hetero)arene (0.2 mmol, if liquid) and methoxycyclopentane (CAPE, 0.5 mL) were added with syringes under a stream of nitrogen. The resulting mixture was stirred at the corresponding temperature for the assigned time. After cooling to room temperature, the reaction mixture was concentrated and then purified by column chromatography on silica gel to give the target product (The spectra data for the borylated compounds can be seen in the Supplementary Material).

#### Analytical Data of products **5 to 5p**

*2-(3,5-Dimethoxyphenyl)-4,4,5,5-tetramethyl-1,3,2-dioxaborolane (**5**)*. [56]. Yield: 47.5 mg (90%); white solid; m.p.: 87–88 °C; ^1^H-NMR (400 MHz, CDCl_3_) δ 6.95 (d, *J* = 2.4 Hz, 2H), 6.57 (t, *J* = 2.4 Hz, 1H), 3.82 (s, 6H), 1.34 (s, 12H); ^13^C-NMR (100 MHz, CDCl_3_) δ 160.5, 111.7, 104.6, 84.0, 55.5, 25.0; ^11^B-NMR (128.4 MHz, CDCl_3_) δ 31.3 (bs).

*4,4,5,5-Tetramethyl-2-(3,4,5-trimethoxyphenyl)-1,3,2-dioxaborolane (**5a**).* Yield: 58.2 mg (99%); white solid; m.p.: 104–106 °C; ^1^H-NMR (400 MHz, CDCl_3_) δ 7.04 (s, 2H), 3.91 (s, 6H), 3.87 (s, 3H), 1.35 (s, 12H); ^13^C-NMR (100 MHz, CDCl_3_) 153.0, 140.9, 111.3, 84.0, 60.9, 56.3, 25.0; ^11^B-NMR (128.4 MHz, CDCl_3_) δ 32.0 (bs). The spectral data were in accordance with the literature [16].

*2-(3-Chloro-5-methoxyphenyl)-4,4,5,5-tetramethyl-1,3,2-dioxaborolane (**5b**)* [31]. Yield: 51.5 mg (96%); colorless oil; ^1^H NMR (400 MHz, CDCl_3_) δ 7.37 (d, *J* = 1.6 Hz, 1H), 7.19 (d, *J* = 2.4 Hz, 1H), 6.99 (t, *J* = 2.4 Hz, 1H), 3.82 (s, 3H), 1.34 (s, 12H); ^13^C-NMR (100 MHz, CDCl_3_) δ 160.0, 134.7, 127.0, 117.9, 117.5, 84.3, 55.7, 25.0; ^11^B-NMR (128.4 MHz, CDCl_3_) δ 30.9 (bs).

*2-(3-Bromo-5-methoxyphenyl)-4,4,5,5-tetramethyl-1,3,2-dioxaborolane (**5c**)* [27]. Yield: 61.8 mg (99%); colorless oil; ^1^H-NMR (400 MHz, CDCl_3_) δ 7.52 (d, *J* = 1.2 Hz, 1H), 7.24 (d, *J* = 2.4 Hz, 1H), 7.15 (t, *J* = 2.0 Hz, 1H), 3.81 (s, 3H), 1.34 (s, 12H); ^13^C-NMR (100 MHz, CDCl_3_) δ 160.0, 129.9, 122.8, 120.7, 118.0, 84.3, 55.7, 25.0; ^11^B-NMR (128.4 MHz, CDCl_3_) δ 30.5 (bs).

*2-(3-Bromo-5-(trifluoromethyl)phenyl)-4,4,5,5-tetramethyl-1,3,2-dioxaborolane (**5d**)* [31]. Yield: 67.9 mg (97%); colorless oil; ^1^H-NMR (400 MHz, CDCl_3_) δ 8.1 (s, 1H), 7.97 (s, 1H), 7.83 (s, 1H), 1.36 (s, 12H); ^13^C-NMR (100 MHz, CDCl_3_), 141.0, 132.1 (q, *J* = 32.7 Hz), 131.0 (q, *J* = 3.7 Hz), 130.0 (q, *J* = 3.7 Hz), 123.4 (q, *J* = 273.0 Hz), 122.6, 84.8, 25.0; ^19^F-NMR (376.5 MHz, CDCl_3_) δ -62.7; ^11^B-NMR (128.4 MHz, CDCl_3_) δ 30.3 (bs).

*Dimethyl 5-(4,4,5,5-tetramethyl-1,3,2-dioxaborolan-2-yl)isophthalate (**5e**)* [57]. Yield: 63.4 mg (99%); white solid; ^1^H-NMR (400 MHz, CDCl_3_) δ 8.77 (t, *J* = 2.0 Hz, 1H), 8.64 (d, *J* = 2.0 Hz, 1H), 3.95 (s, 1H) 1.37 (s, 12H); ^13^C-NMR (100 MHz, CDCl_3_) δ 166.4, 140.0, 133.4, 130.2, 84.5, 52.4, 25.0; ^11^B-NMR (128.4 MHz, CDCl_3_) δ 30.6 (bs).

*2-(3-Bromo-2-fluoro-5-(trifluoromethyl)phenyl)-4,4,5,5-tetramethyl-1,3,2-dioxaborolane (**5f**)* [6]. Yield: 72.1 mg (98%); white solid; ^1^H-NMR (400 MHz, CDCl_3_) δ 7.98–7.93 (m, 1H), 7.91 (dd, *J* = 6.0, 2.0 Hz, 1H), 1.37 (s, 12H); ^13^C-NMR (100 MHz, CDCl_3_) δ 164.9 (d, *J* = 258.5 Hz), 133.9 (m), 133.2 (m), 127.6 (dd, *J* = 33.8, 3.7 Hz), 123.1 (q, *J* = 272.5 Hz), 110.2 (d, *J* = 24.6 Hz), 84.9, 24.9; ^19^F-NMR (376.5 MHz, CDCl_3_) δ –62.0, –91.0; ^11^B-NMR (128.4 MHz, CDCl_3_) δ 29.5 (bs).

*2-(3-Bromo-5-(trifluoromethoxy)phenyl)-4,4,5,5-tetramethyl-1,3,2-dioxaborolane (**5g**)* [6]. Yield: 70.2 mg (96%); colorless oil; ^1^H-NMR (400 MHz, CDCl_3_) δ 7.86 (d, *J* = 1.2 Hz, 1H), 7.56 (s, 1H), 7.46 (s, 1H), 1.34 (s, 12H); ^13^C-NMR (100 MHz, CDCl_3_) δ 149.3, 136.1, 127.1, 125.5, 122.6, 120.5 (q, *J* = 257.9 Hz), 84.7, 25.0; ^19^F-NMR (376.5 MHz, CDCl_3_) δ –57.8; ^11^B-NMR (128.4 MHz, CDCl_3_) δ 30.2 (bs).

*2-(4-Bromo-2,6-difluorophenyl)-4,4,5,5-tetramethyl-1,3,2-dioxaborolane (**5h**)* [6]. Yield: 63.0 mg (99%); colorless oil; ^1^H-NMR (400 MHz, CDCl_3_) δ 7.05 (m, 2H), 1.37 (s, 12H); ^13^C-NMR (100 MHz, CDCl_3_) δ 166.6 (dd, *J* = 254.0, 14.0 Hz), 125.7 (t, *J* = 12.8 Hz), 115.3 (m), 84.5, 24.8; ^19^F-NMR (376.5 MHz, CDCl_3_) δ –99.8(s); ^11^B-NMR (128.4 MHz, CDCl_3_) δ 29.7 (bs). 

*2,6-Dichloro-4-(4,4,5,5-tetramethyl-1,3,2-dioxaborolan-2-yl)pyridine **(5i**)* [6]. Yield: 52.4 mg (96%); white solid; ^1^H-NMR (400 MHz, CDCl_3_) δ 7.59 (s, 2H), 1.35 (s, 12H); ^13^C-NMR (100 MHz, CDCl_3_) δ 150.5, 127.9, 85.4, 25.0; ^11^B-NMR (128.4 MHz, CDCl_3_) δ 29.8 (bs).

*4,4,5,5-Tetramethyl-2-(perfluorophenyl)-1,3,2-dioxaborolane (**5j**)* [24]. Yield: 58.2 mg (99%); colorless oil; ^1^H NMR (400 MHz, CDCl_3_) δ 1.38 (s, 12H); ^13^C-NMR (100 MHz, CDCl_3_) δ 85.1, 24.8; ^19^F-NMR (376.5 MHz, CDCl_3_) δ –129.5 (m, 2F), –149.7 (m, 1F), –161.9 (m, 2F); ^11^B-NMR (128.4 MHz, CDCl_3_) δ 29.2 (bs).

*2-(3,4-Dibromophenyl)-4,4,5,5-tetramethyl-1,3,2-dioxaborolane (**5k**)* [58]. Yield: 70.0 mg (97%); colorless oil; ^1^H-NMR (400 MHz, CDCl_3_) δ 8.03 (s, 1H), 7.62 (d, *J* = 8.0 Hz, 1H), 7.55 (dd, *J* = 8.0, 1.2 Hz, 1H) 1.34 (s, 12H); ^13^C-NMR (100 MHz, CDCl_3_) δ 139.8, 134.6,133.4, 128.3, 124.8, 84.5, 25.0; ^11^B-NMR (128.4 MHz, CDCl_3_) δ 30.6 (bs).

*4,4,5,5-Tetramethyl-2-(2,4,6-trimethoxyphenyl)-1,3,2-dioxaborolane (**5l**)* [6]. Yield: 30.1 mg (20%); white solid; ^1^H-NMR (400 MHz, CDCl_3_) δ 6.04 (s, 2H), 3.79 (s, 3H), 3.75 (s, 6H), 1.36 (s, 12H); ^13^C-NMR (100 MHz, CDCl_3_) δ 164.7, 163.3, 90.4, 83.6, 55.8, 55.4, 24.8; ^11^B-NMR (128.4 MHz, CDCl_3_) δ 32.5 (bs).

*(4r,8r)-4-Methyl-2,6-dioxo-8-(5-(4,4,5,5-tetramethyl-1,3,2-dioxaborolan-2-yl)thiophen-3-yl)hexahydro-[1,3,2]oxazaborolo[2,3-b][1,3,2]oxazaborol-4-ium-8-uide (**5m**)* [53]. Yield: 71.6 mg (98%); white solid; ^1^H-NMR (400 MHz, DMSO-d_6_) δ 7.89 (d, *J* = 0.8, 1H), 7.61 (d, *J* = 0.8 1H), 4.32 (d, *J* = 17.2Hz, 2H), 4.13 (d, *J* = 17.2Hz, 2H), 2.57 (s, 3H), 1.29(s, 12H). ^13^C-NMR (100 MHz, DMSO-d_6_) δ 169.6, 142.5, 139.0, 84.3, 62.2, 47.9, 25.1.

*(4r,8r)-8-(2,4-difluoro-3,5-bis(4,4,5,5-tetramethyl-1,3,2-dioxaborolan-2-yl)phenyl)-4-methyl-2,6-dioxohexahydro-[1,3,2]oxazaborolo[2,3-b][1,3,2]oxazaborol-4-ium-8-uide (**5n**)* [53]. Yield: 103.6 mg (99%); white solid; ^1^H-NMR (400 MHz, DMSO-*d*_6_) δ 7.90 (t, *J* = 8, 1H), 4.40 (d, *J* = 17.2Hz, 2H), 4.13 (d, *J* = 17.2Hz, 2H), 2.64 (s, 3H), 1.30 (s, 12H). ^13^C-NMR (100 MHz, DMSO-d_6_) δ 169.5, 84.5, 84.2, 66.8, 63.2, 48.3, 25.1, 24.9.

*2-(6-chlorobenzo[d][1,3]dioxol-4-yl)-4,4,5,5-tetramethyl-1,3,2-dioxaborolane (**5o**)* [6]. Yield: 37.8 mg (67%); white solid; ^1^H-NMR (400 MHz, CDCl_3_) δ 7.17 (d, *J* = 2.0 Hz, 1H), 6.86 (d, *J* = 2.4 Hz, 1H), 6.04 (s, 2H), 1.36 (s, 12H); ^13^C-NMR (100 MHz, CDCl_3_) δ 151.6, 148.1, 127.0, 126.0, 112.0, 102.0, 84.4, 24.9; ^11^B-NMR (128.4 MHz, CDCl_3_) δ 30.0 (bs).

*2,2′-(5-chlorobenzo[d][1,3]dioxole-4,7-diyl)bis(4,4,5,5-tetramethyl-1,3,2-dioxaborolane) (**5p**)* [6]. Yield: 65.3 mg (80%); white solid; ^1^H-NMR (400 MHz, CDCl_3_) δ 7.16 (s, 1H), 6.06 (s, 2H), 1.39 (s, 12H), 1.35 (s, 12H); ^13^C-NMR (100 MHz, CDCl_3_) δ 152.2, 150.6, 129.6, 127.4, 101.9, 84.7, 84.3, 24.95, 24.86; ^11^B-NMR (128.4 MHz, CDCl_3_) δ 31.5 (bs).

## 5. Conclusions

In summary, we designed and synthesized a new tridentate boryl anion NNB-type ligand and applied it to the iridium-catalyzed C–H borylation reaction as a very highly active iridium catalyst precursor. It is also suitable for the highly electron-rich arenes, and arenes with large steric hindrance. Due to the recombination of the bidentate N and boryl anion ligands, enlarging the electron density of the iridium center sped up the oxidative addition process with the C–H bond. This research may inspire the discovery of other metal complexes with the NNB ligand, while further investigation for the new catalytic systems continues.

## Figures and Tables

**Table 1:**
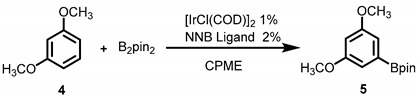
Variations from the “standard” reaction conditions of iridium-catalyzed C–H borylation ^a^.

Entry	Ligand	B_2_pin_2_	Temperature	Time	Yield(NMR) ^b^
1	dtbpy	1.0	100	3h	72%
2	1,10-ph	1.0	100	3h	90%
3	tmphen	1.o	100	3h	94%
**4**	**L1**	**1.0**	**100**	**3h**	**94% (90% ^c^)**
5 ^d^	Ir-NNB complex	1.0	100	3h	92%
6	L1	0.5	100	3 h	64%
7	L1	0.6	100	3 h	68%
8	L1	0.7	100	3 h	71%
9	L1	0.8	100	3 h	82%
10	L1	1.1	100	3 h	86%
11	L1	1.0	100	0.5 h	25%
12	L1	1.0	100	1 h	81%
13	L1	1.0	100	2 h	89%
14	L1	1.0	80	3 h	66%
15	L1	1.0	90	3 h	72%
16	L2	1.0	100	3 h	90%
17	L3	1.0	100	3 h	82%

*^a^* Reaction: 1,3-dimethoxybenzene (0.2 mmol), NNB ligand (4 µmol, 2.0 mmol%), [Ir(Cl)(COD)]_2_ (2 µmol, 1.0 mmol%) and B_2_pin_2_ (x mmol), 0.5 mL solvent, 2 h. *^b^* Determined by NMR analysis of the reaction mixture using 1,3,5-trimethoxybenzene as an internal standard; yield was based on 1,3-dimethoxylbenzene. *^c^* Yield of the isolated product. *^d^* Using Ir-NNB complex in the standard reaction condition. Dtbpy: 4,4′-di-tert-butyl bipyridine; 1,10-phen: 1,10-phenanthroline; tmphen: 3,4,7,8-tetramethyl-1,10-phenanthroline; CPME: cyclophenyl methyl ether.

## Supplementary Materials

Supplementary Materials are available online. Experimental procedures and spectral data for the borylated products.
