# Correction: Ding, S., et al. NNB-Type Tridentate Boryl Ligands Enabling a Highly Active Iridium Catalyst for C–H Borylation. *Molecules* 2019, *24*, 1434

**DOI:** 10.3390/molecules24091750

**Published:** 2019-05-06

**Authors:** Siyi Ding, Linghua Wang, Zongcheng Miao, Pengfei Li

**Affiliations:** 1 Key Laboratory of Organic Polymer Photoelectric Materials, School of Science, Xijing University, Xi’an 710123, China; dingsiyi2009@163.com; 2 Center for Organic Chemistry, Frontier Institute of Science and Technology, Xi’an Jiaotong University, Xi’an 710054, China; wanglinghua1036@126.com

The authors wish to make the following corrections to this paper [1]:

In the graphic abstract, the dosage for the B_2_pin_2_ was incorrectly displayed. The correct version of the table is as follows:

Graphic Abstract: 
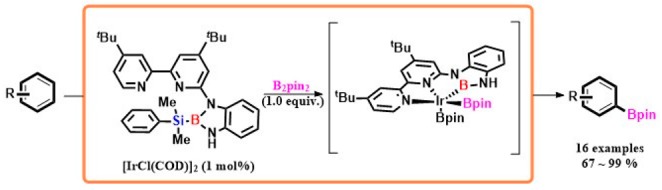


In Scheme 3, the structures of the starting materials for preparation of the preL_2_ and preL_3_ were incorrectly displayed. The correct version of the scheme is as follows:

The changes do not affect the scientific outcome. The manuscript will be updated and the original will remain online on the article webpage. The authors would like to apologize for any inconvenience caused to the readers by these changes.

## Figures and Tables

**Scheme 3:**
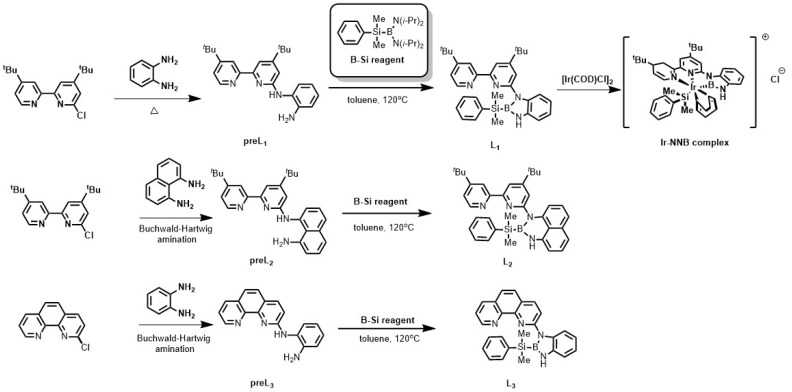
Preparation of the NNB ligand and the oxidative addition with [Ir(COD)Cl]_2_ and NNB ligand 1.
