# Supplementary material for: NNB-Type Tridentate Boryl Ligands Enabling a Highly Active Iridium Catalyst for C–H Borylation
Source: Molecules. 2019 Apr 11;24(7):1434. doi: 10.3390/molecules24071434 (PMC6479588; doi:10.3390/molecules24071434)
Supplement: Supplementary file 1 [file molecules-24-01434-s001.pdf]

Supporting Information

NNB-Type Tridentate Boryl Ligands Enabling a Highly Active Iridium-catalyst for C–H Borylation

Table of Contents

1. NNB-Type Tridentate Boryl Ligands Enabling a Highly Active Iridium-catalyst for C-H Borylation 1

1.1 Optimization Reactions 1

2. Copy of NMR spectra 3

1. NNB-Type Tridentate Boryl Ligands Enabling a Highly Active Iridium-catalyst for C–H Borylation

1.1 Optimization Reactions

Table S1. Ir-NNB ligand catalytic system for aryl borylation *via* C–H bond cleavage

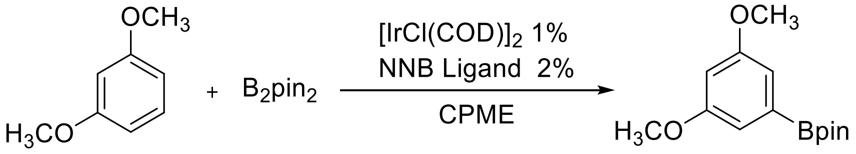

NNB ligand	B_2pin_2(eq.)	Reaction temperature	Reaction time	Yield (NMR)[b]
**1 (L1)**	1.0	100	3h	94%(90%[c])
**2 (L1)**	0.5	100	3h	64%
**3 (L1)**	0.6	100	3h	68%
**4 (L1)**	0.7	100	3h	71%
**5 (L1)**	0.8	100	3h	82%
**6 (L1)**	1.1	100	3h	86%
**7 (L1)**	1.0	100	0.5h	25%
**8 (L1)**	1.0	100	1h	82%
**9 (L1)**	1.0	100	2h	89%
**10 (L1)**	1.0	80	3h	66%
**11 (L2)**	1.0	100	3h	90%
**12 (L3)**	1.0	100	3h	82%

[a] Reaction conditions： 1,3-dimethoxylbenzene (0.2 mmol), NNB ligand (4 μmol, 2.0 mmol%), $[Ir(Cl)(COD)]_2$ (2 μmol, 1.0 mmol%) and $(Bpin)_2$ (x mmol), solvent 0.5 mL, 2 hours; [b] NMR yield using tridecane as internal standard.

Table 4-2 Screening for the optimal reaction conditions via the Ir-NNB ligand catalytic system

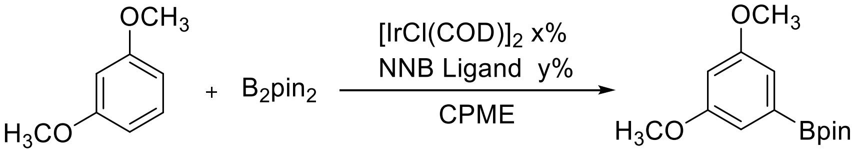

Entry	[Ir] x %	NNB ligand y %	Reaction time	Yield (NMR)[b]
**1**	0.1	0.2	3	60
**2**	0.1	0.2	6	70
**3**	0.1	0.2	18	90
**4**	0.2	0.4	3	80
**5**	0.2	0.4	6	95
**6**	0.2	0.4(dtbpy)	3	72

Entry	[Ir] x %	NNB ligand y %	Reaction time	Yield (NMR)[b]
**7**	0.2	0.4(phen)	3	90
**8**	0.2	0.4(me-phen)	3	94

[a] Reaction conditions：1,3-dimethoxylbenzene (2 mmol), NNB ligand (y mmol%), $[Ir(Cl)(COD)]_2$ (x mmol%) and $(Bpin)_2$ (2 mmol), solvent 0.5 mL, 2 hours; [b]NMR yield using tridecane as internal standard; dtbpy: 4,4'-di^{t}butyl-2,2'-bipyridine; Phen: 1,10-phenanthroline; me-phen: 3,4,7,8-tetramethyl-1,10-phenanthroline.

2. Copy of NMR spectra

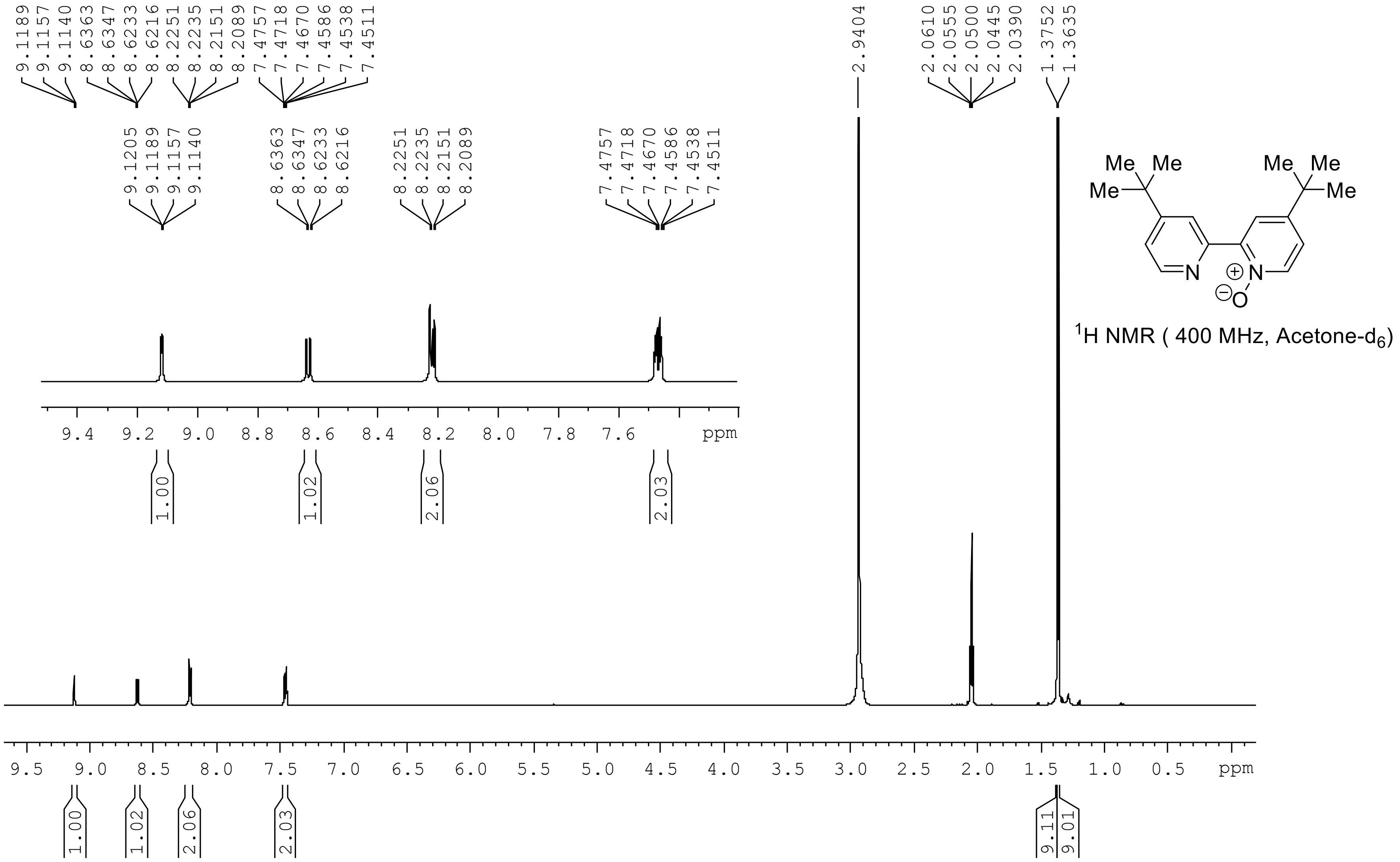

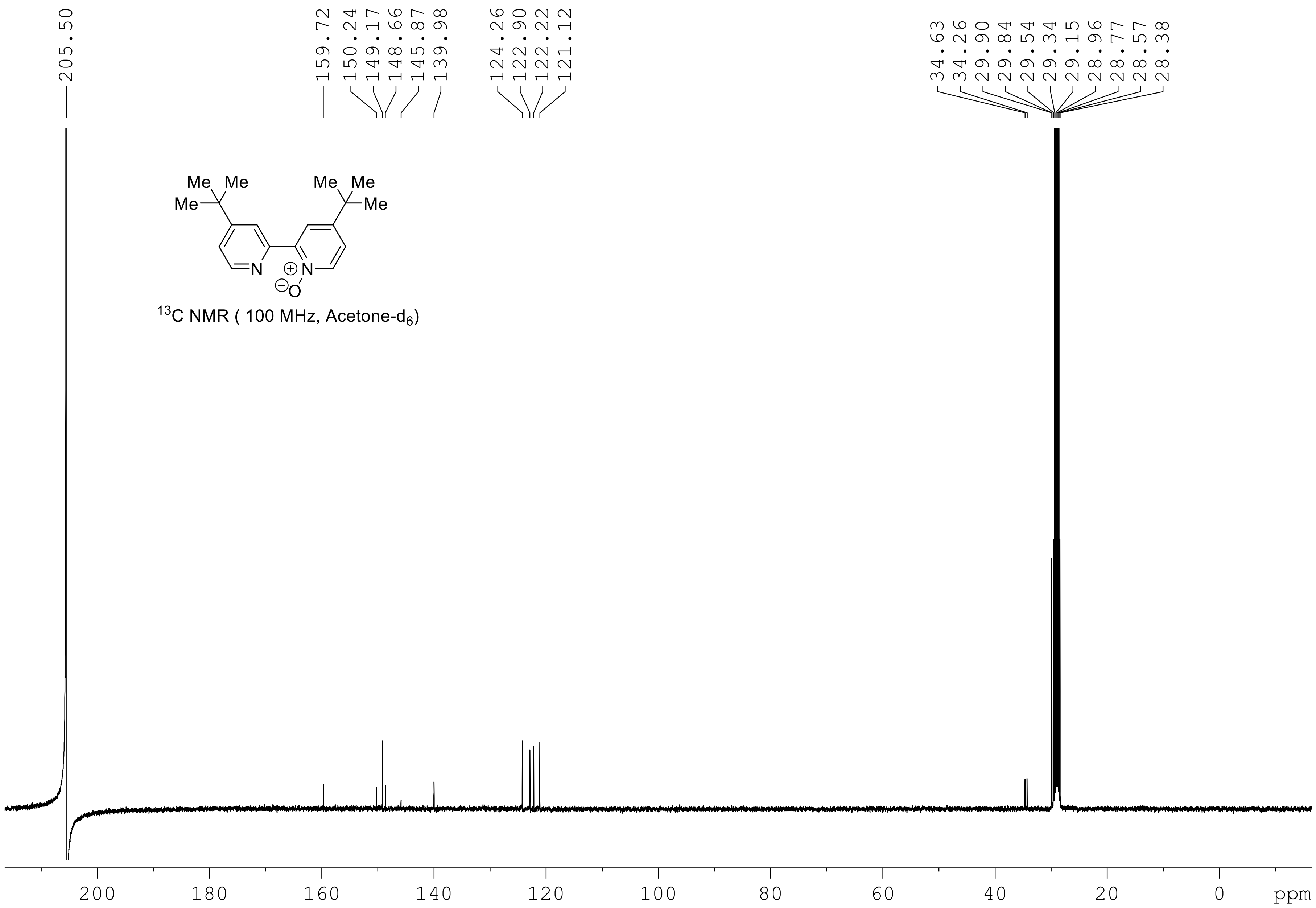
205.50
159.72
150.24
149.17
148.66
145.87
139.98
124.26
122.90
122.22
121.12
34.63
34.26
29.90
29.84
29.54
29.34
29.15
28.96
28.77
28.57
28.38
Me
Me
Me
Me
Me
Me
N
N
O
^{13}C NMR (100 MHz, Acetone-d_6)
200
180
160
140
120
100
80
60
40
20
0
ppm

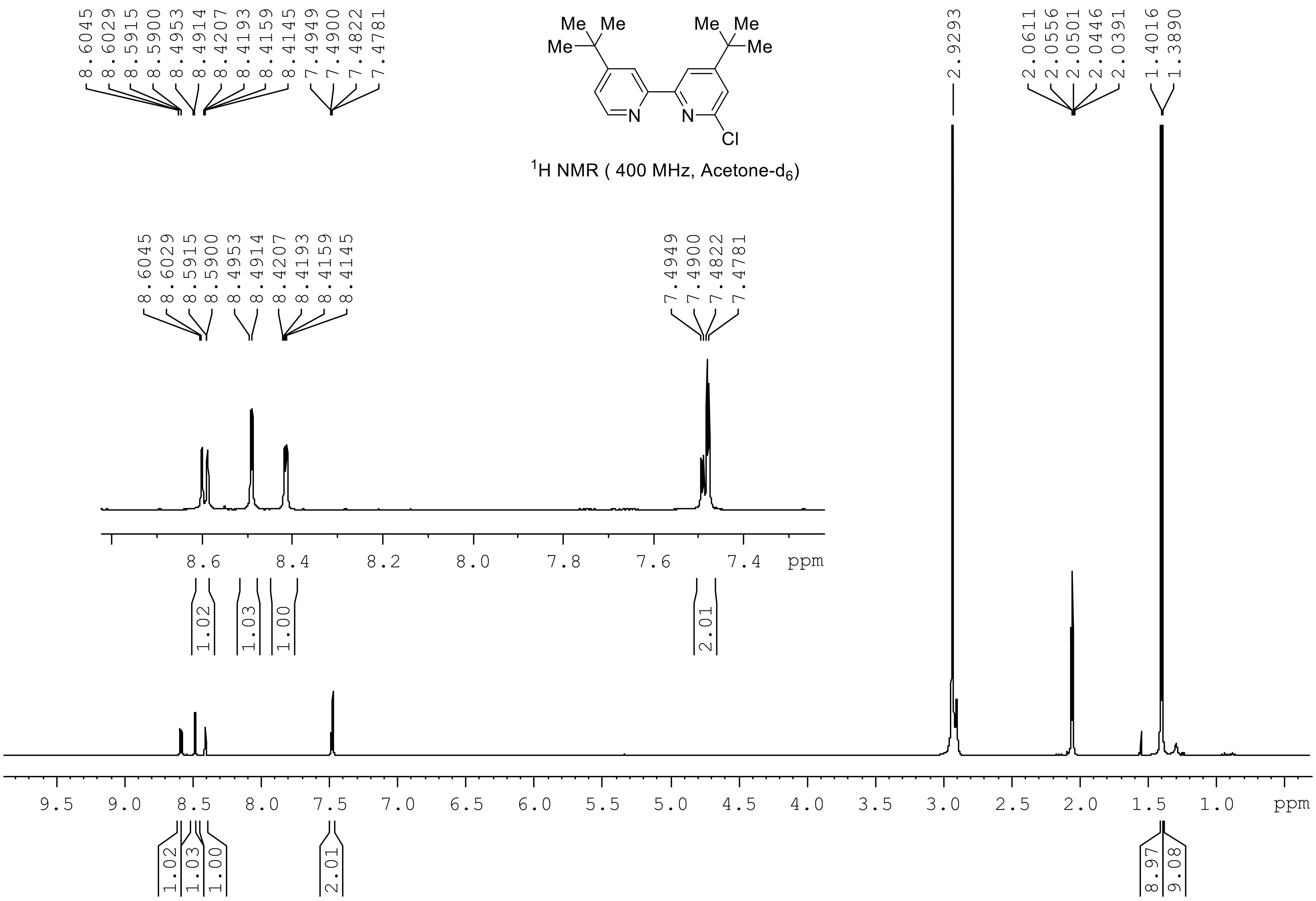
Me Me
Me
Me Me
Me
N
N
Cl
^{1}H NMR (400 MHz, Acetone-d_6)
8.6045 8.6029 8.5915 8.5900 8.4953 8.4914 8.4207 8.4193 8.4159 8.4145 7.4949 7.4900 7.4822 7.4781
2.9293
2.0611 2.0556 2.0501 2.0446 2.0391 1.4016 1.3890
8.6 8.4 8.2 8.0 7.8 7.6 7.4 ppm
1.02 1.03 1.00 2.01
9.5 9.0 8.5 8.0 7.5 7.0 6.5 6.0 5.5 5.0 4.5 4.0 3.5 3.0 2.5 2.0 1.5 1.0 ppm
8.97 9.08

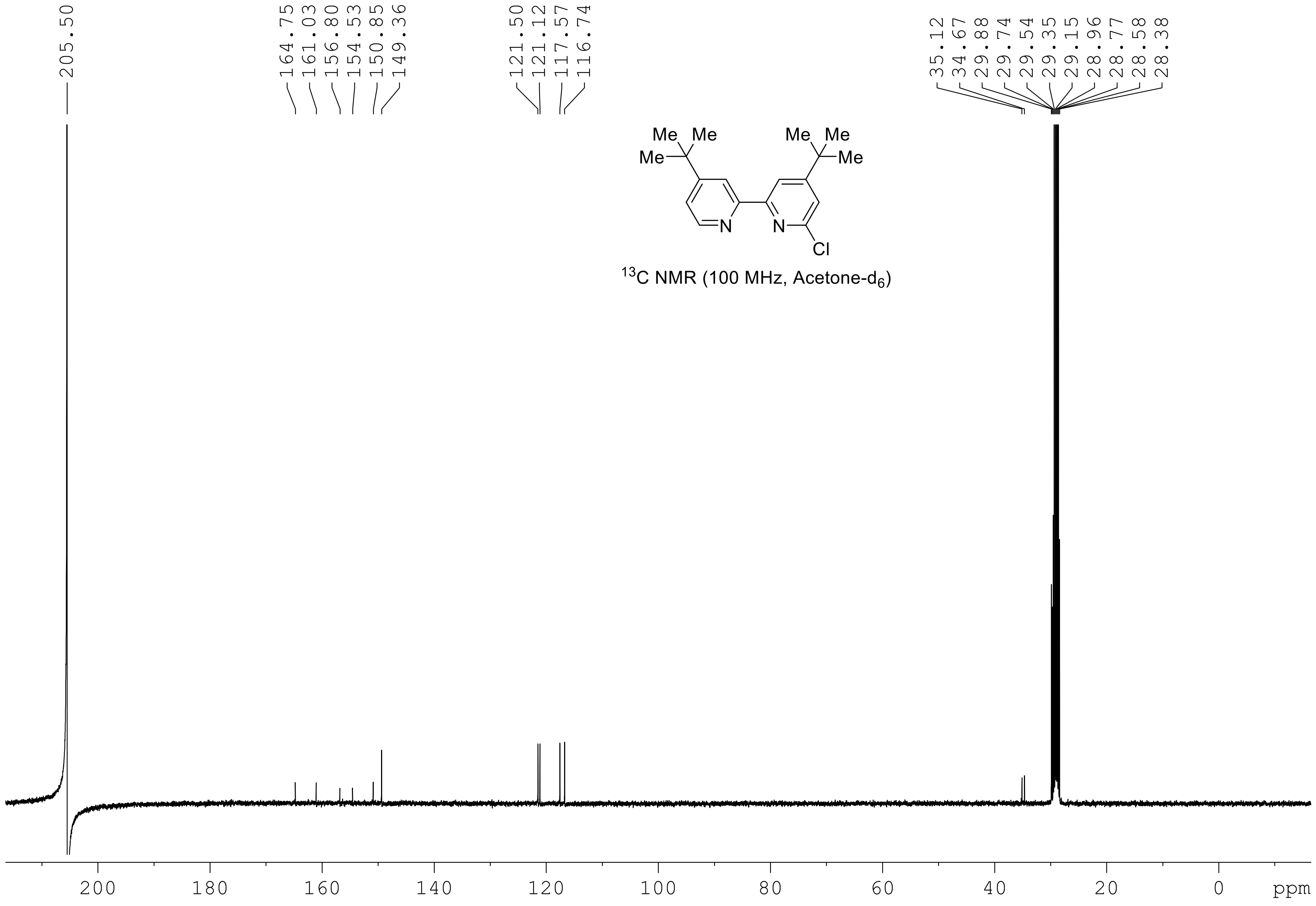
205.50
164.75
161.03
156.80
154.53
150.85
149.36
121.50
121.12
117.57
116.74
35.12
34.67
29.88
29.74
29.54
29.35
29.15
28.96
28.77
28.58
28.38
Me
Me
Me
Me
Me
Me
N
N
Cl
^{13}C NMR (100 MHz, Acetone-d$_6$)
200
180
160
140
120
100
80
60
40
20
0
ppm

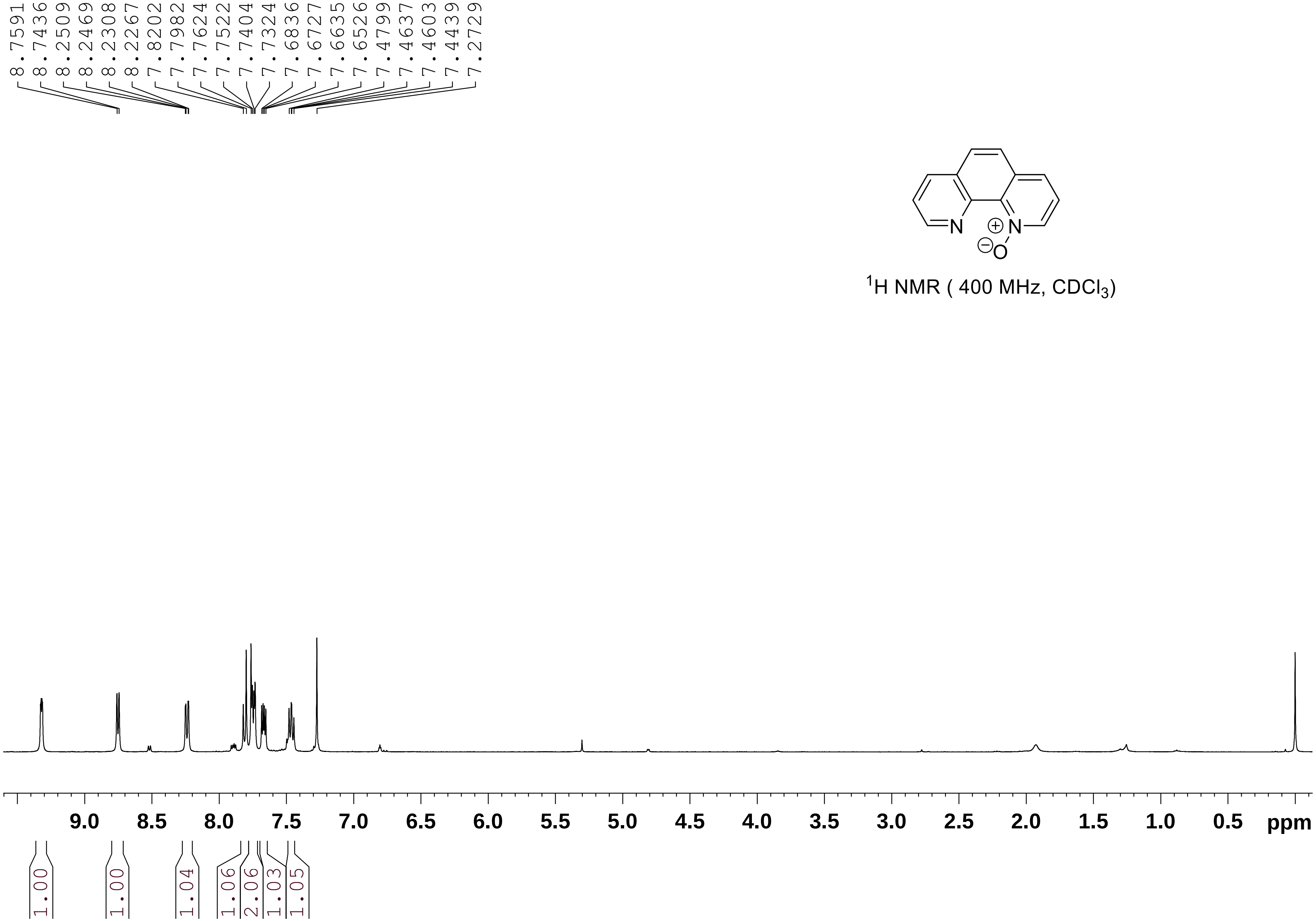

^{1}H NMR (400 MHz, $CDCl_3$)

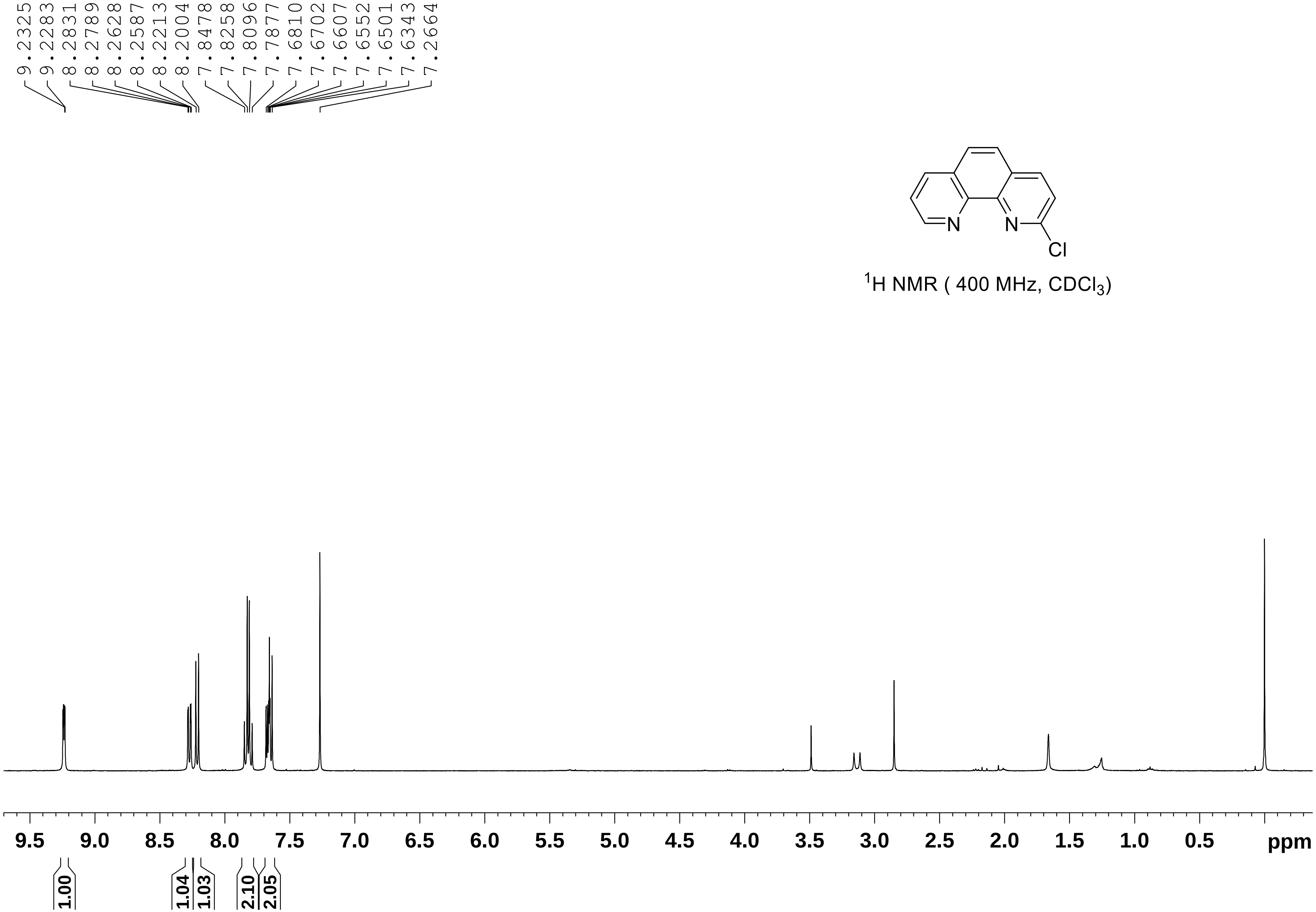

^1H NMR (400 MHz, $CDCl_3$)

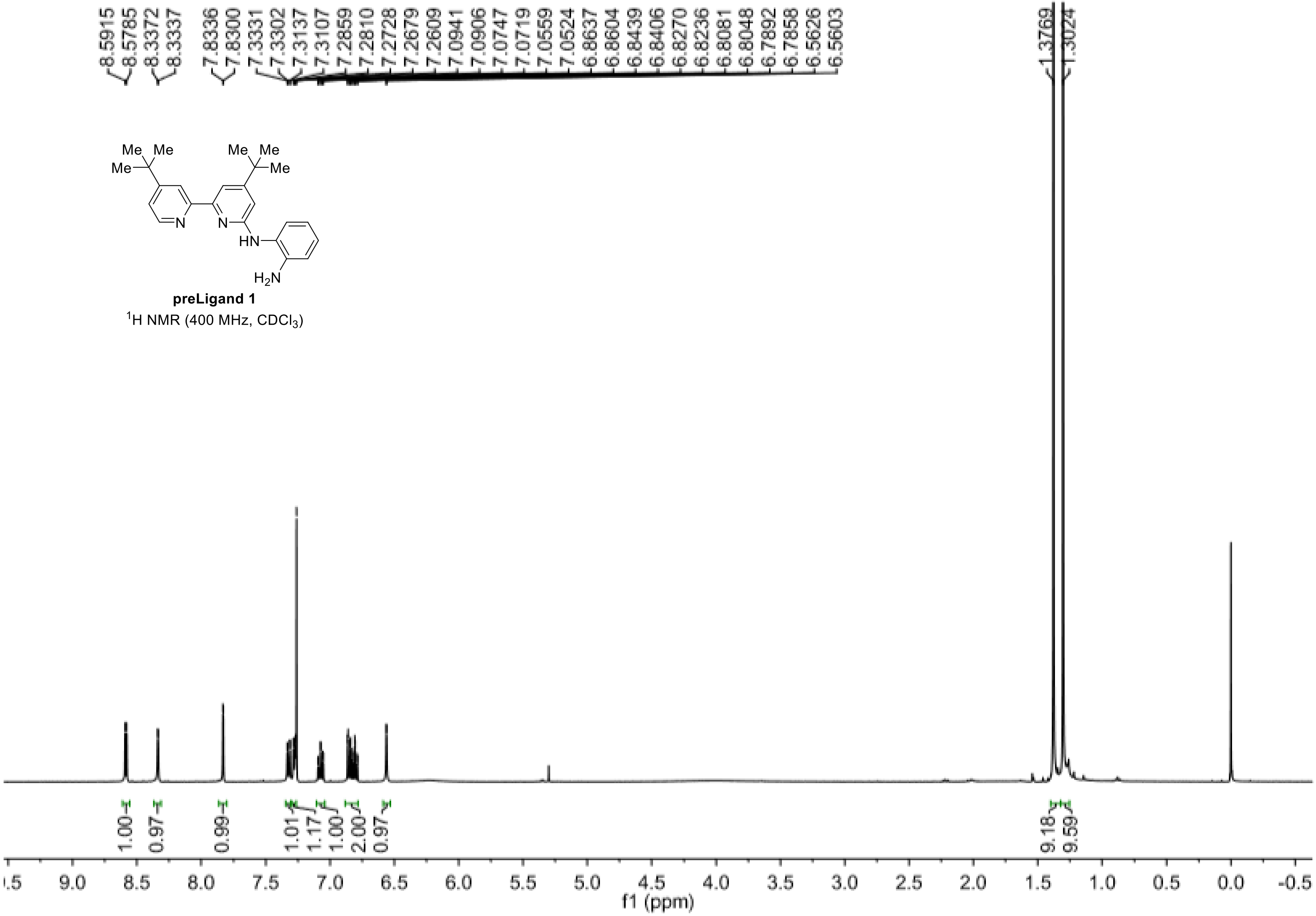
8.5915
8.5785
8.3372
8.3337
7.8336
7.8300
7.3331
7.3302
7.3137
7.3107
7.2859
7.2810
7.2728
7.2679
7.2609
7.0941
7.0906
7.0747
7.0719
7.0559
7.0524
6.8637
6.8604
6.8439
6.8406
6.8270
6.8236
6.8081
6.8048
6.7892
6.7858
6.5626
6.5603
1.3769
1.3024
Me
Me
Me
Me
Me
Me
N
N
HN
H_2N
preLigand 1
^{1}H NMR (400 MHz, $CDCl_3$)
1.00
0.97
0.99
1.01
1.17
1.00
2.00
0.97
9.18
9.59
f1 (ppm)

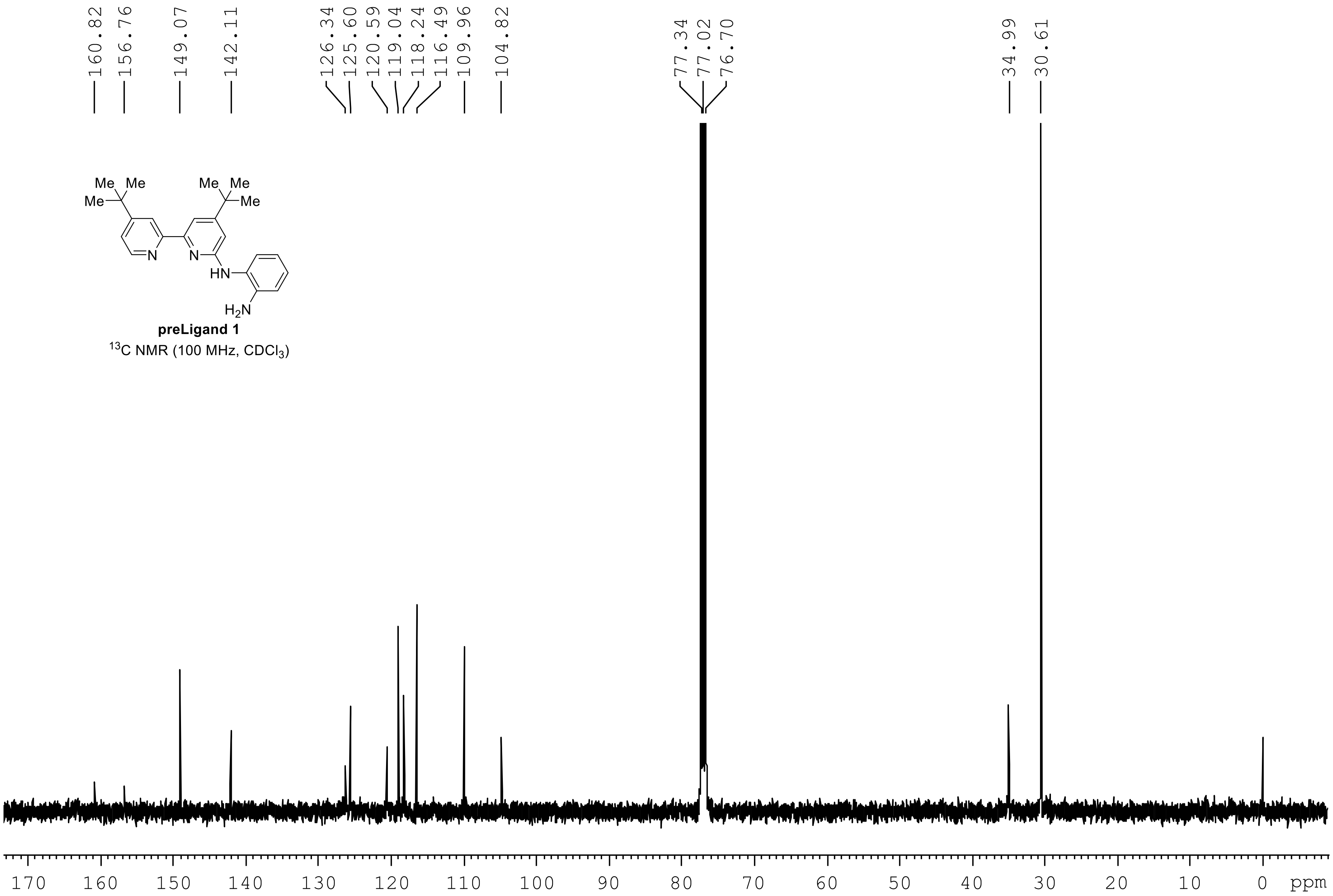

preLigand 1

^{13}C NMR (100 MHz, $CDCl_3$)

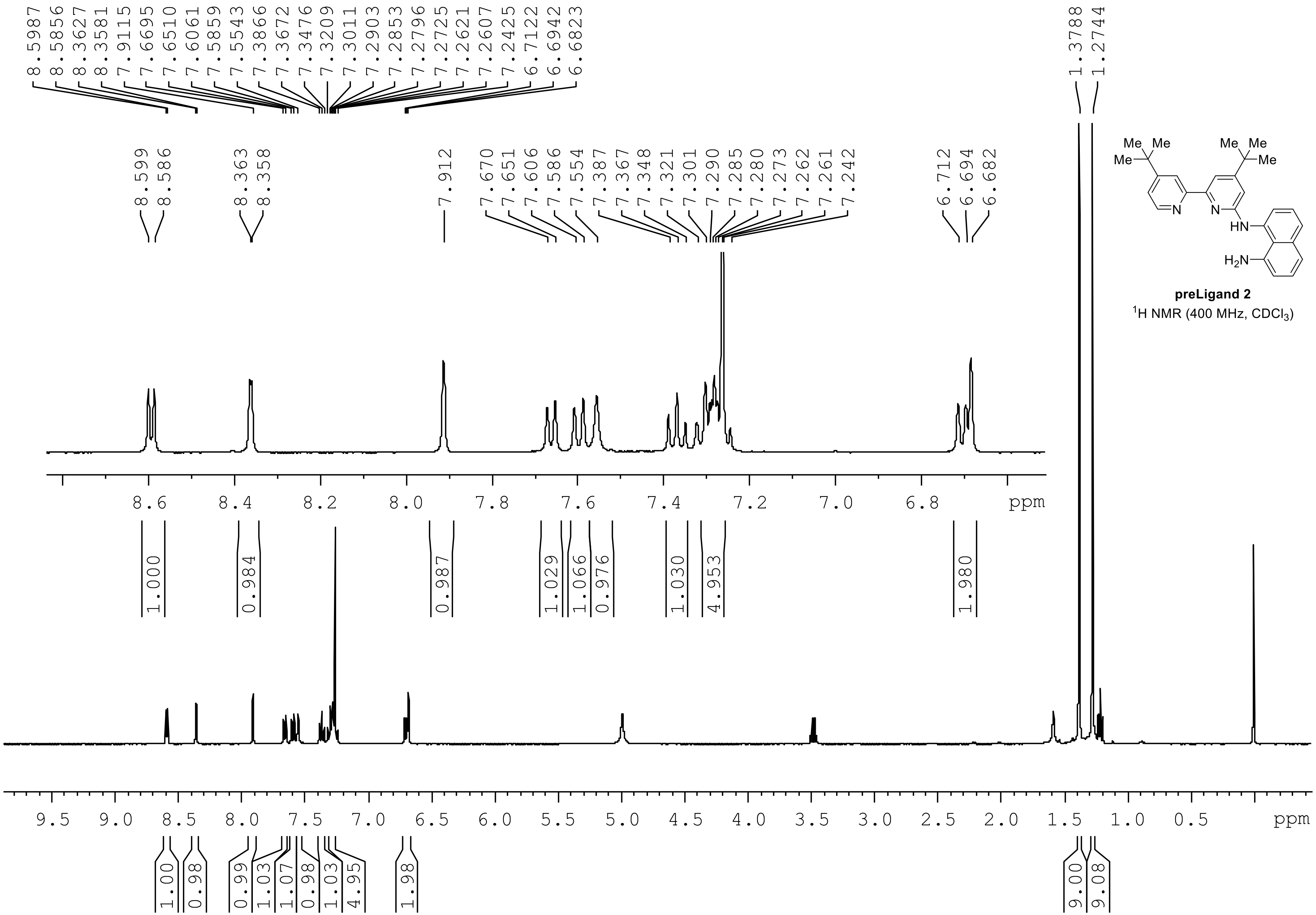

preLigand 2
^{1}H NMR (400 MHz, CDCl$_3$)
ppm

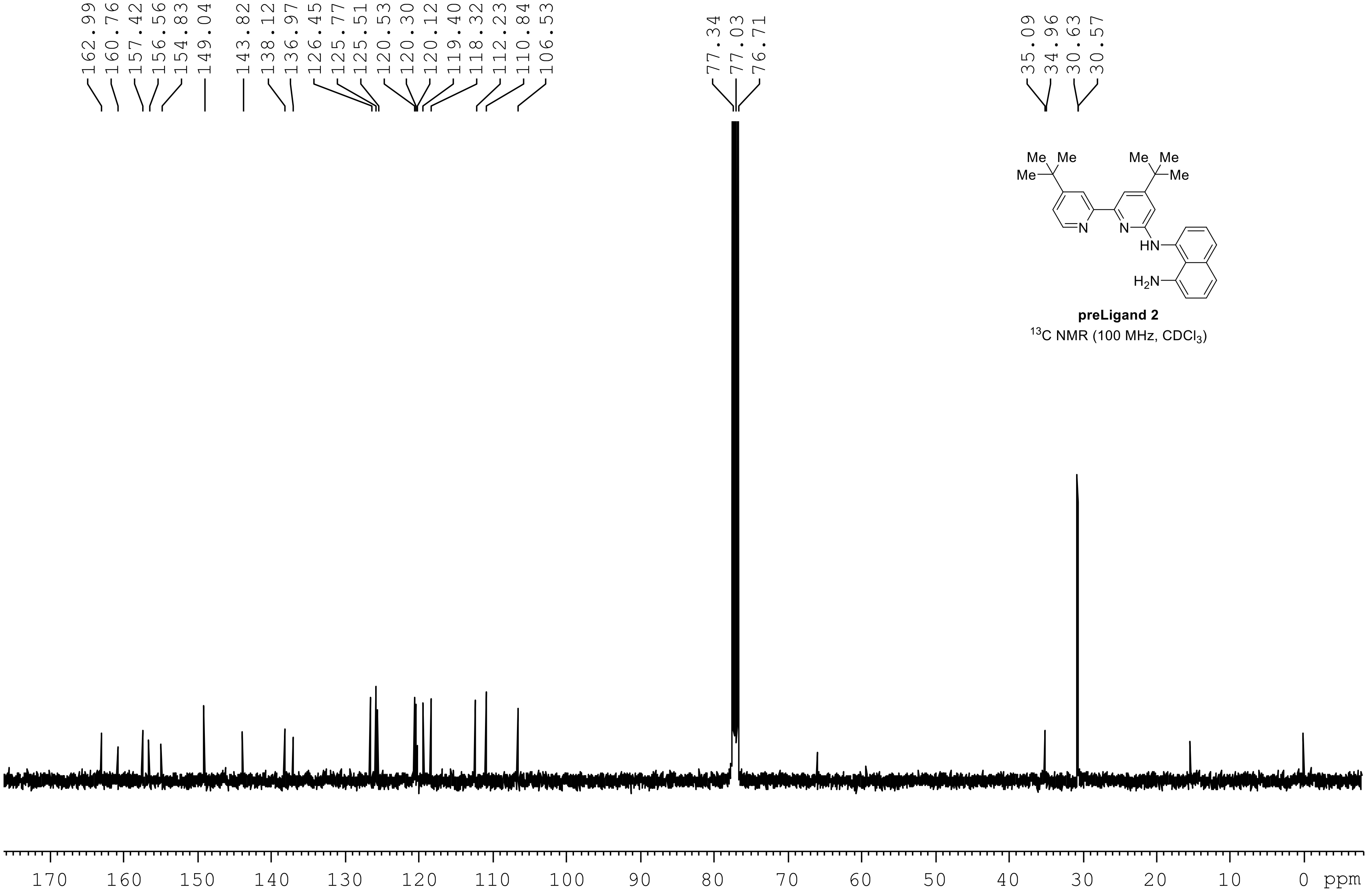

preLigand 2

^{13}C NMR (100 MHz, $CDCl_3$)

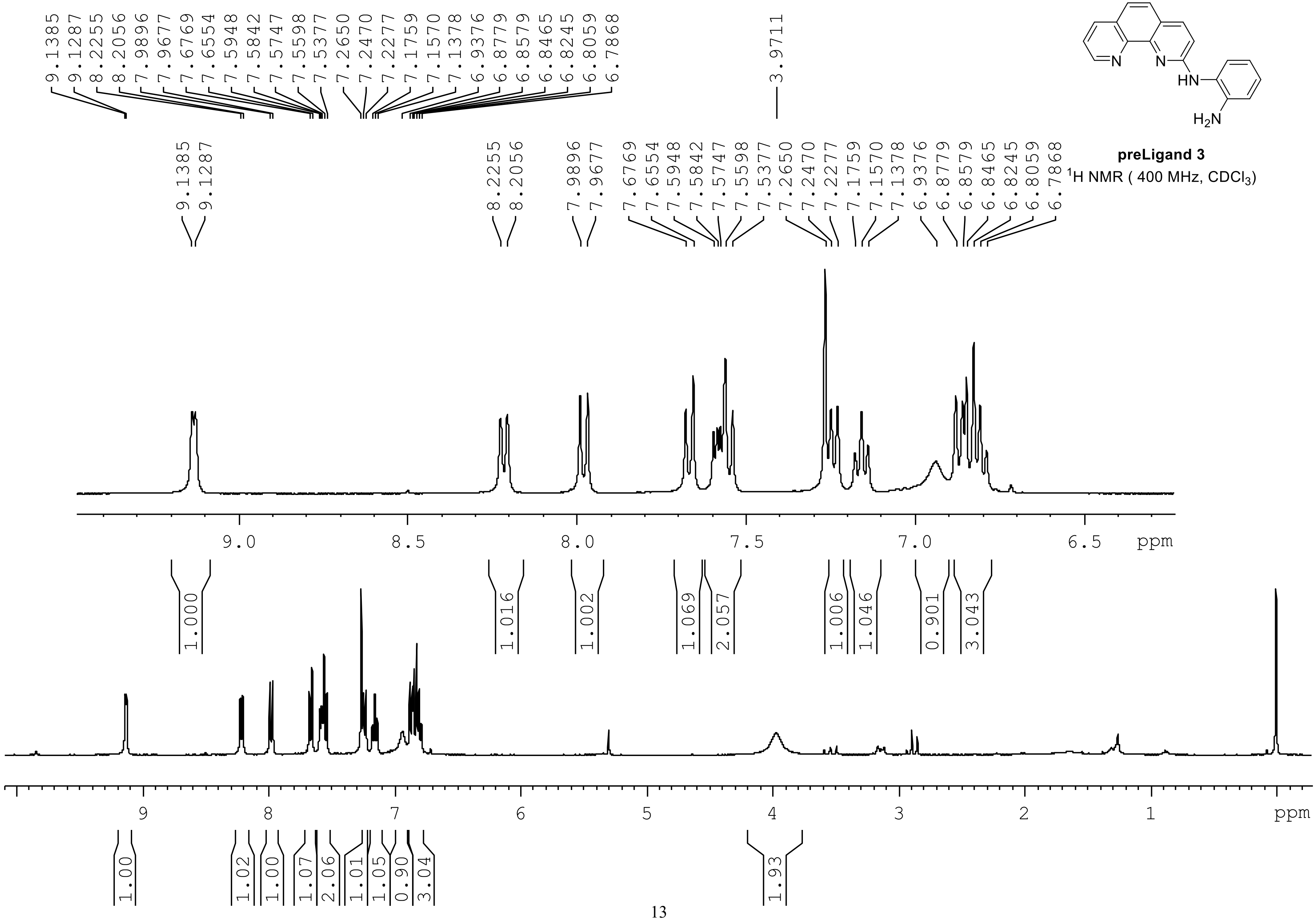

preLigand 3

^{1}H NMR (400 MHz, $CDCl_3$)

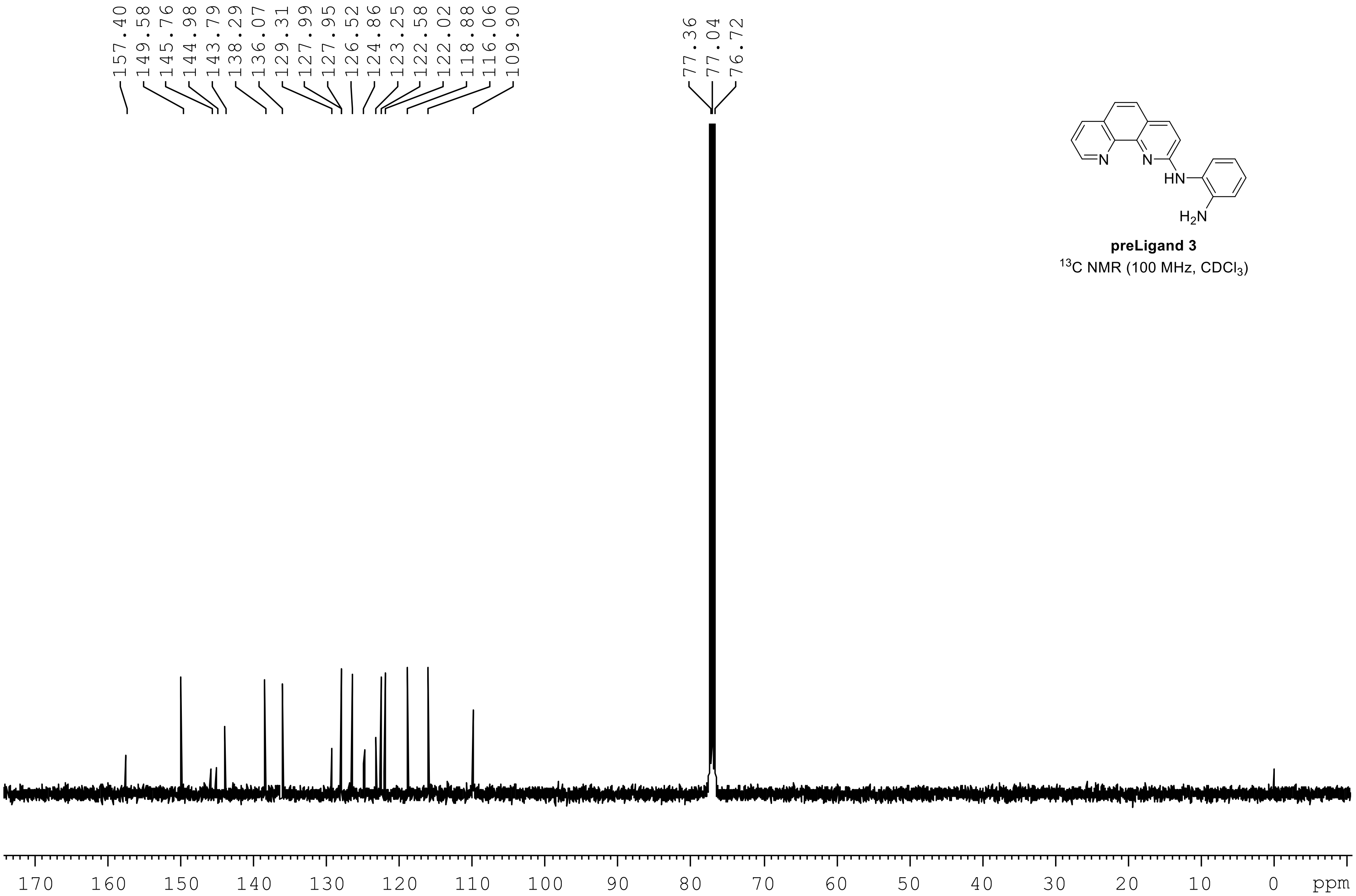

preLigand 3

^{13}C NMR (100 MHz, $CDCl_3$)

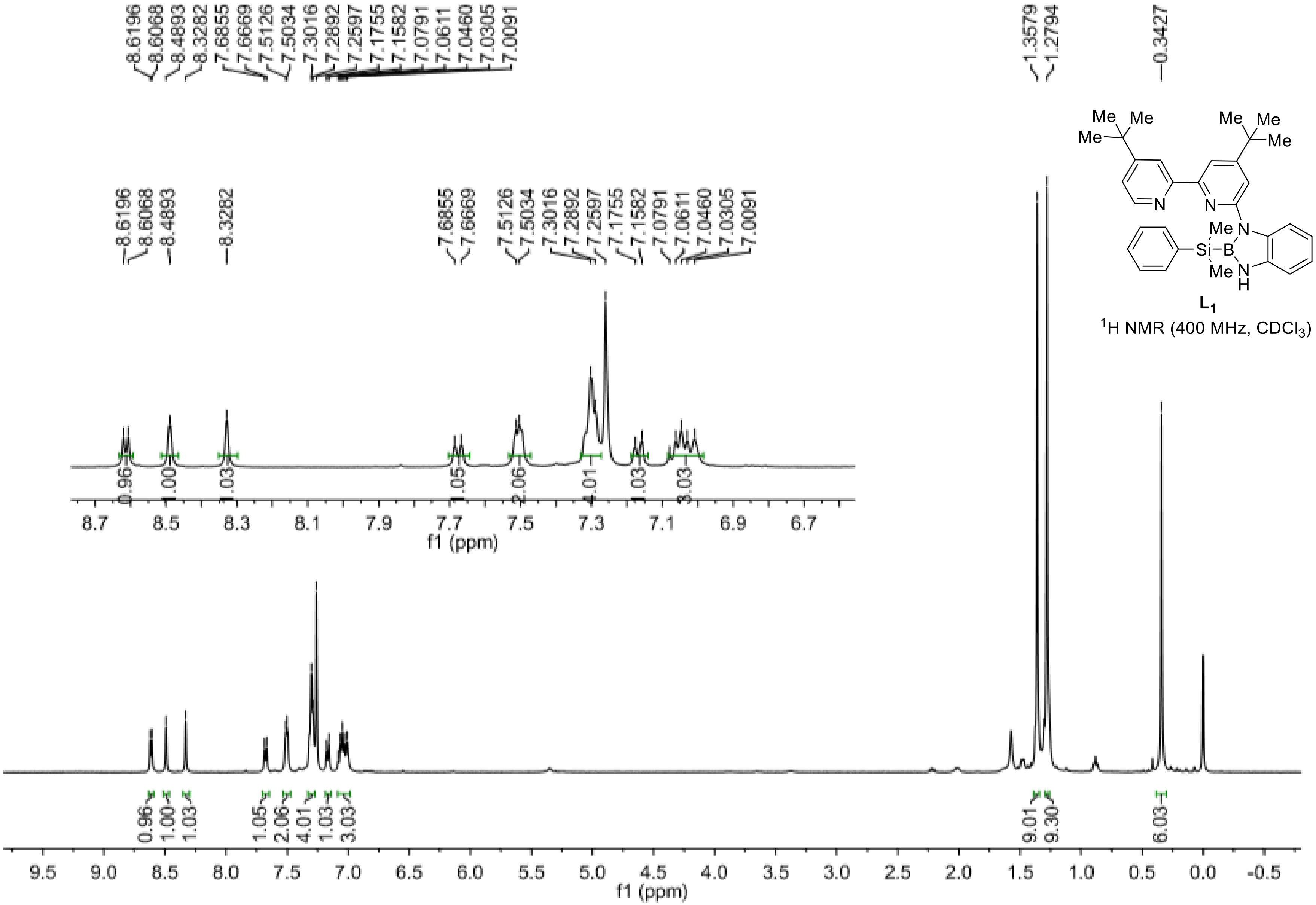
8.6196
8.6068
8.4893
8.3282
7.6855
7.6669
7.5126
7.5034
7.3016
7.2892
7.2597
7.1755
7.1582
7.0791
7.0611
7.0460
7.0305
7.0091
1.3579
1.2794
0.3427
L1
1H NMR (400 MHz, CDCl3)
f1 (ppm)

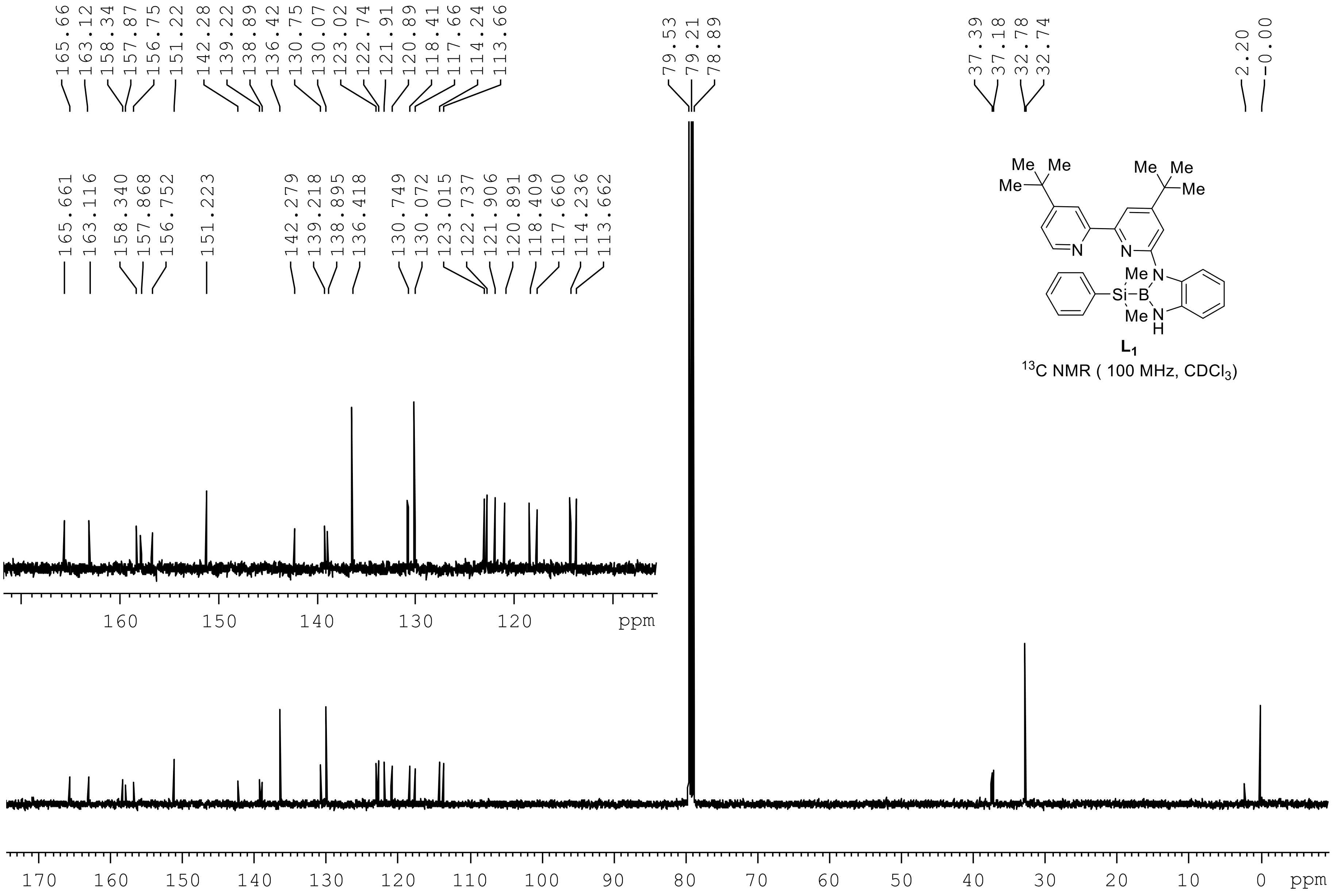
165.66
163.12
158.34
157.87
156.75
151.22
142.28
139.22
138.89
136.42
130.75
130.07
123.02
122.74
121.91
120.89
118.41
117.66
114.24
113.66
79.53
79.21
78.89
37.39
37.18
32.78
32.74
2.20
-0.00
L1
13C NMR (100 MHz, CDCl3)
ppm

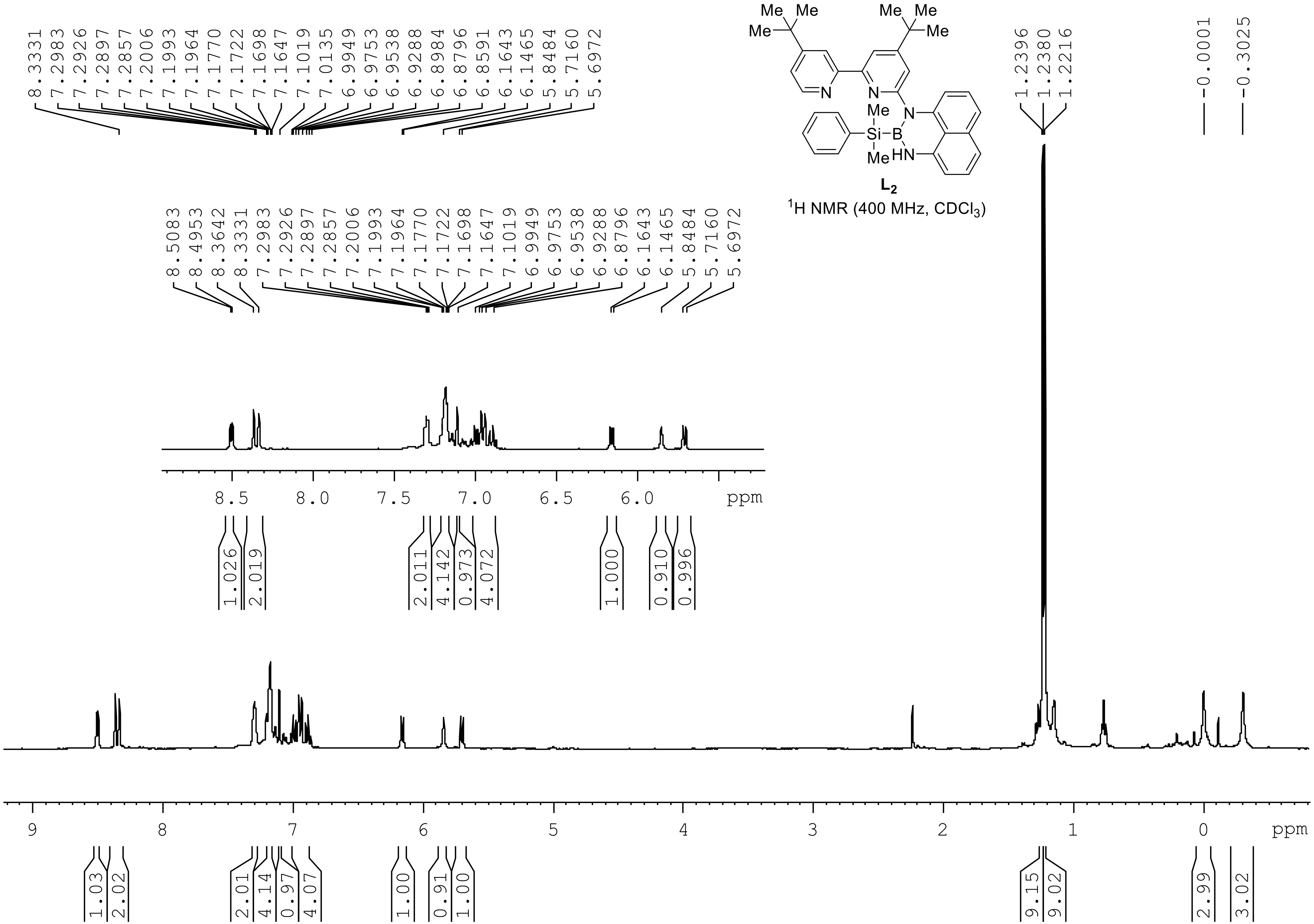
8.3331 7.2983 7.2926 7.2897 7.2857 7.2006 7.1993 7.1964 7.1770 7.1722 7.1698 7.1647 7.1019 7.0135 6.9949 6.9753 6.9538 6.9288 6.8984 6.8796 6.8591 6.1643 6.1465 5.8484 5.7160 5.6972
8.5083 8.4953 8.3642 8.3331 7.2983 7.2926 7.2897 7.2857 7.2006 7.1993 7.1964 7.1770 7.1722 7.1698 7.1647 7.1019 6.9949 6.9753 6.9538 6.9288 6.8796 6.1643 6.1465 5.8484 5.7160 5.6972
1.2396 1.2380 1.2216
-0.0001
-0.3025
Me Me Me Me Me Me
N N N Me Si B HN Me
L2
1H NMR (400 MHz, CDCl3)
8.5 8.0 7.5 7.0 6.5 6.0 ppm
1.026 2.019 2.011 4.142 0.973 4.072 1.000 0.910 0.996
9 8 7 6 5 4 3 2 1 0 ppm
1.03 2.02 2.01 4.14 0.97 4.07 1.00 0.91 1.00 9.15 9.02 2.99 3.02

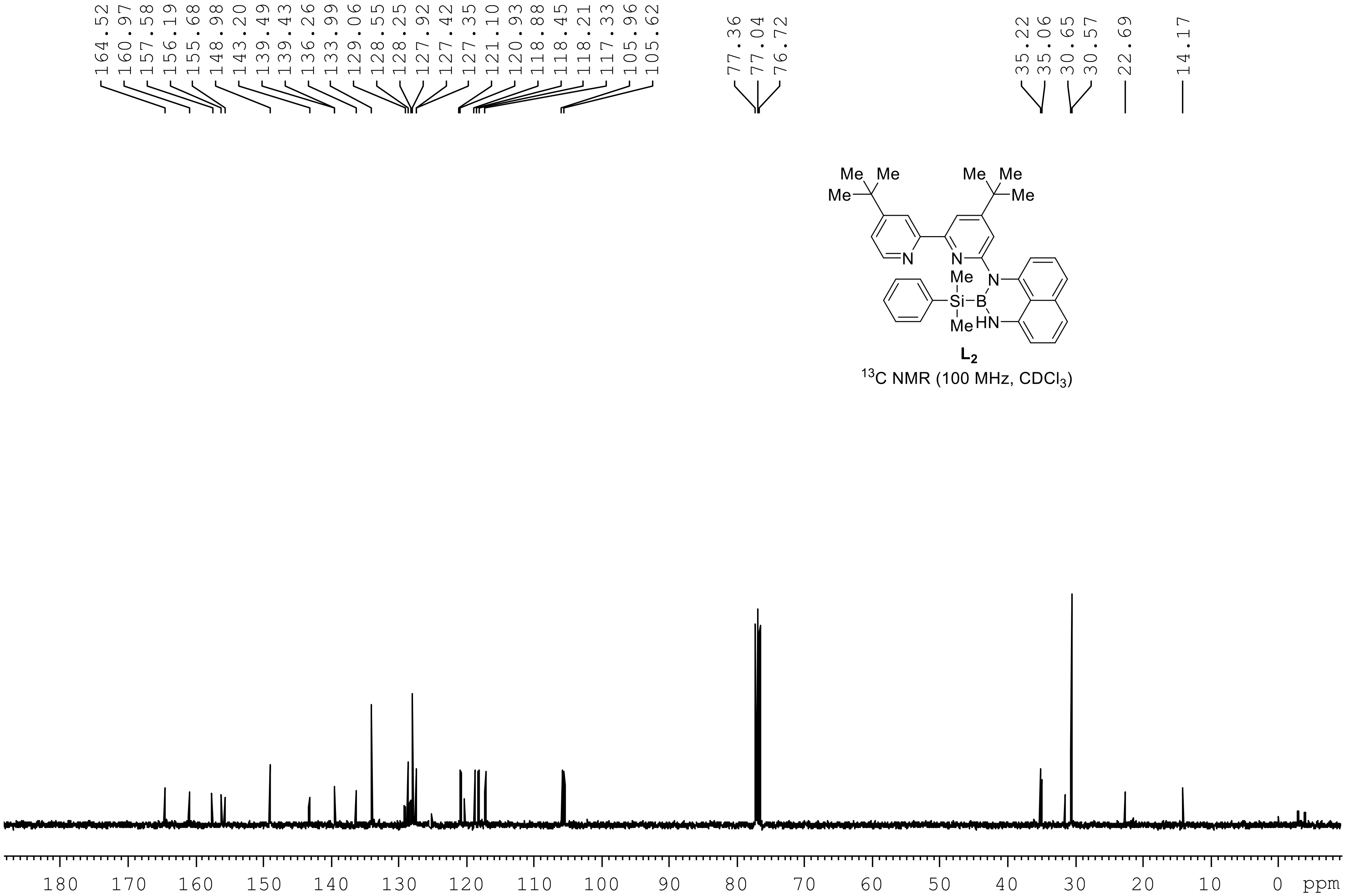
164.52
160.97
157.58
156.19
155.68
148.98
143.20
139.49
139.43
136.26
133.99
129.06
128.55
128.25
127.92
127.42
127.35
121.10
120.93
118.88
118.45
118.21
117.33
105.96
105.62
77.36
77.04
76.72
35.22
35.06
30.65
30.57
22.69
14.17
Me Me
Me
Me Me
Me
N
N
N
Me
Si
B
Me
HN
L2
13C NMR (100 MHz, CDCl3)
180 170 160 150 140 130 120 110 100 90 80 70 60 50 40 30 20 10 0 ppm

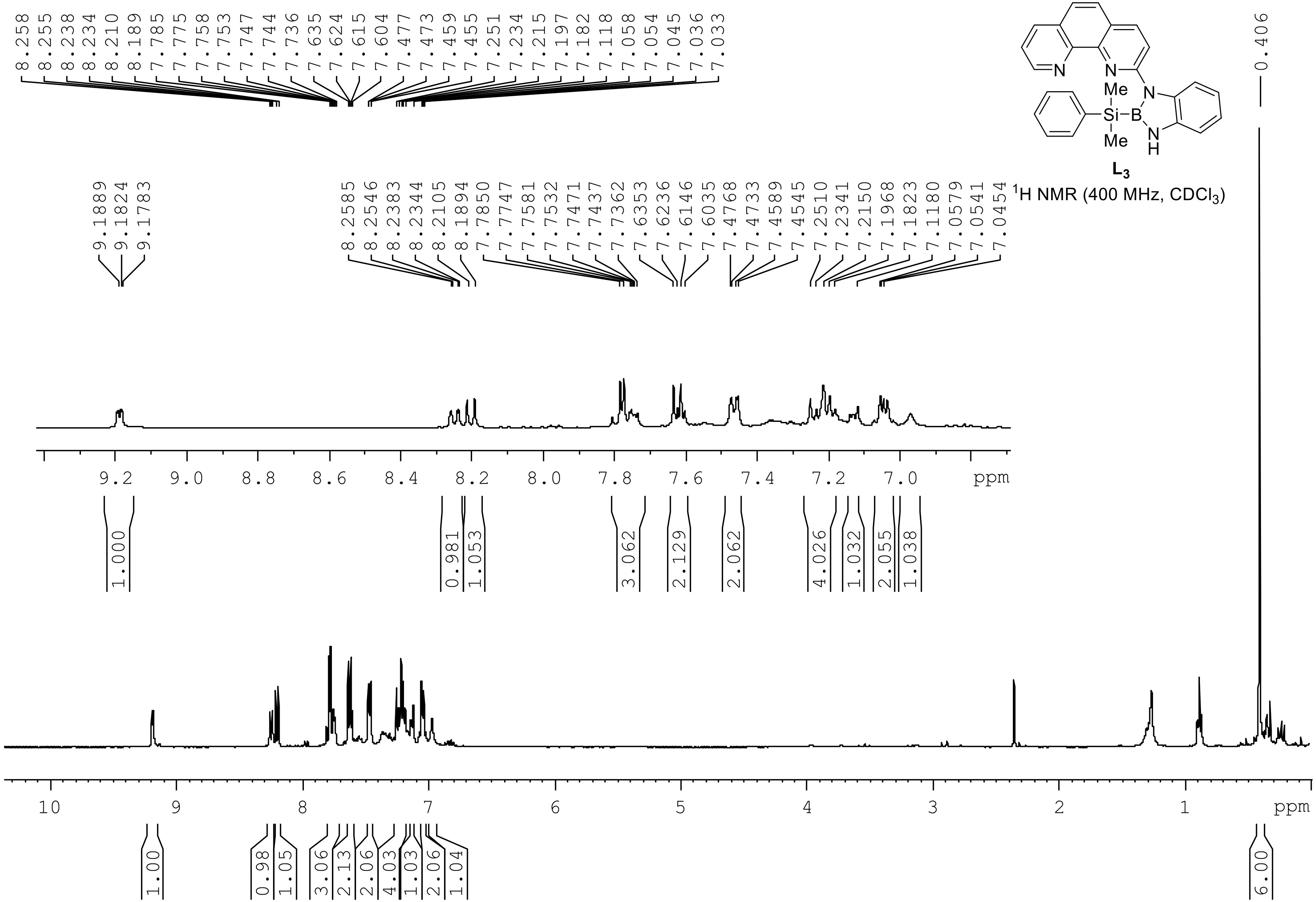
L3
1H NMR (400 MHz, CDCl3)
ppm

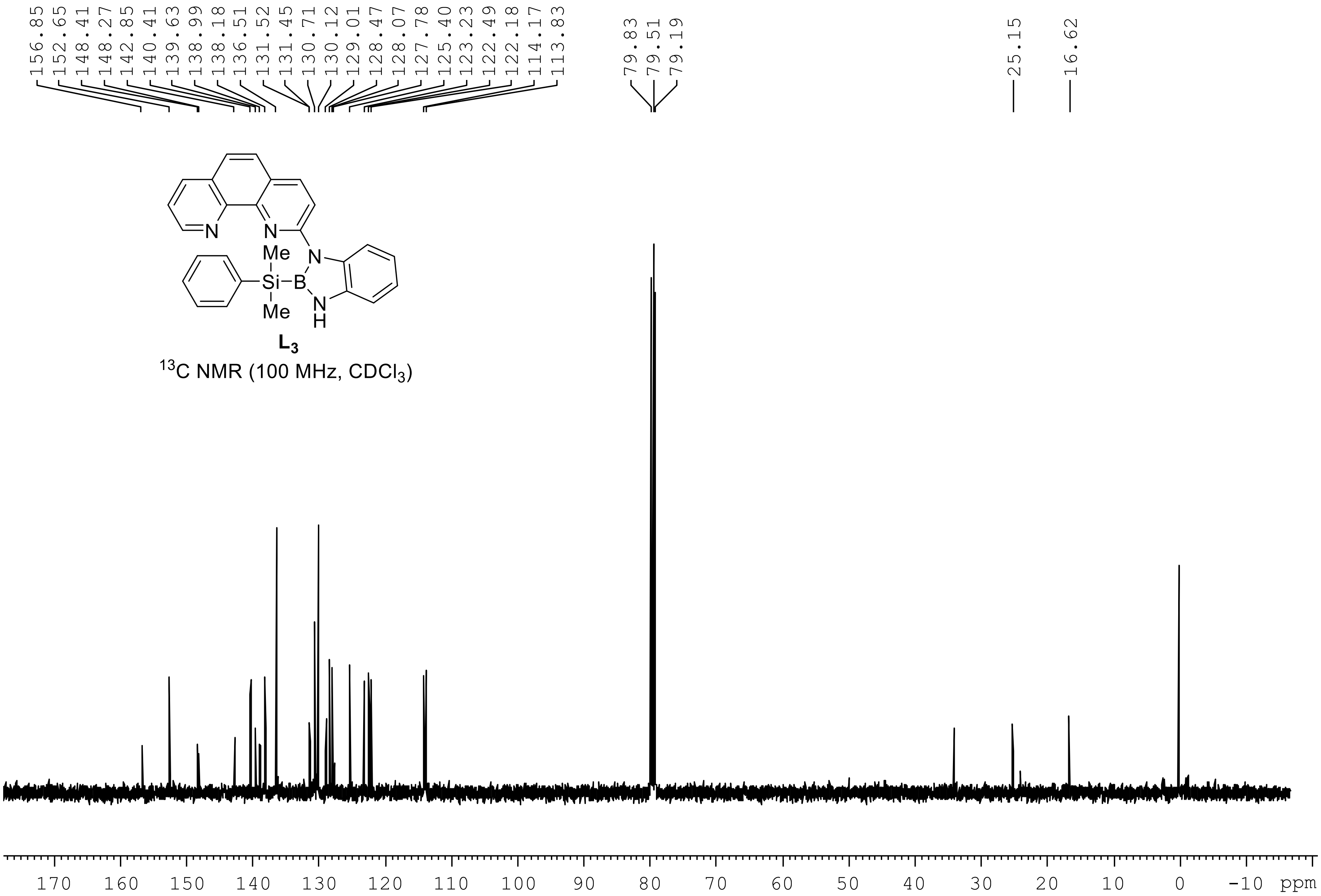

^{13}C NMR (100 MHz, $CDCl_3$)

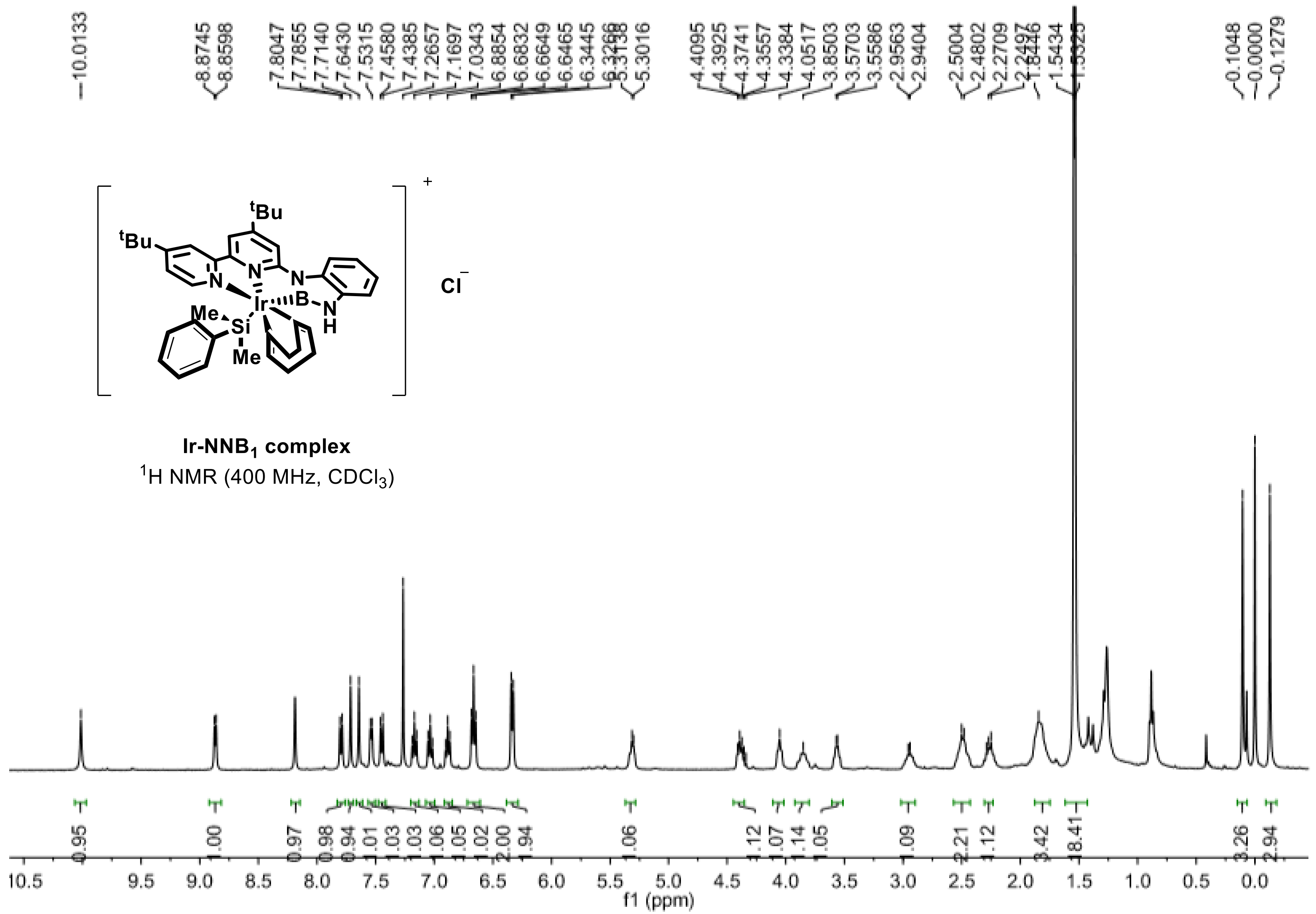

Ir-NNB$_1$ complex
^{1}H NMR (400 MHz, $CDCl_3$)

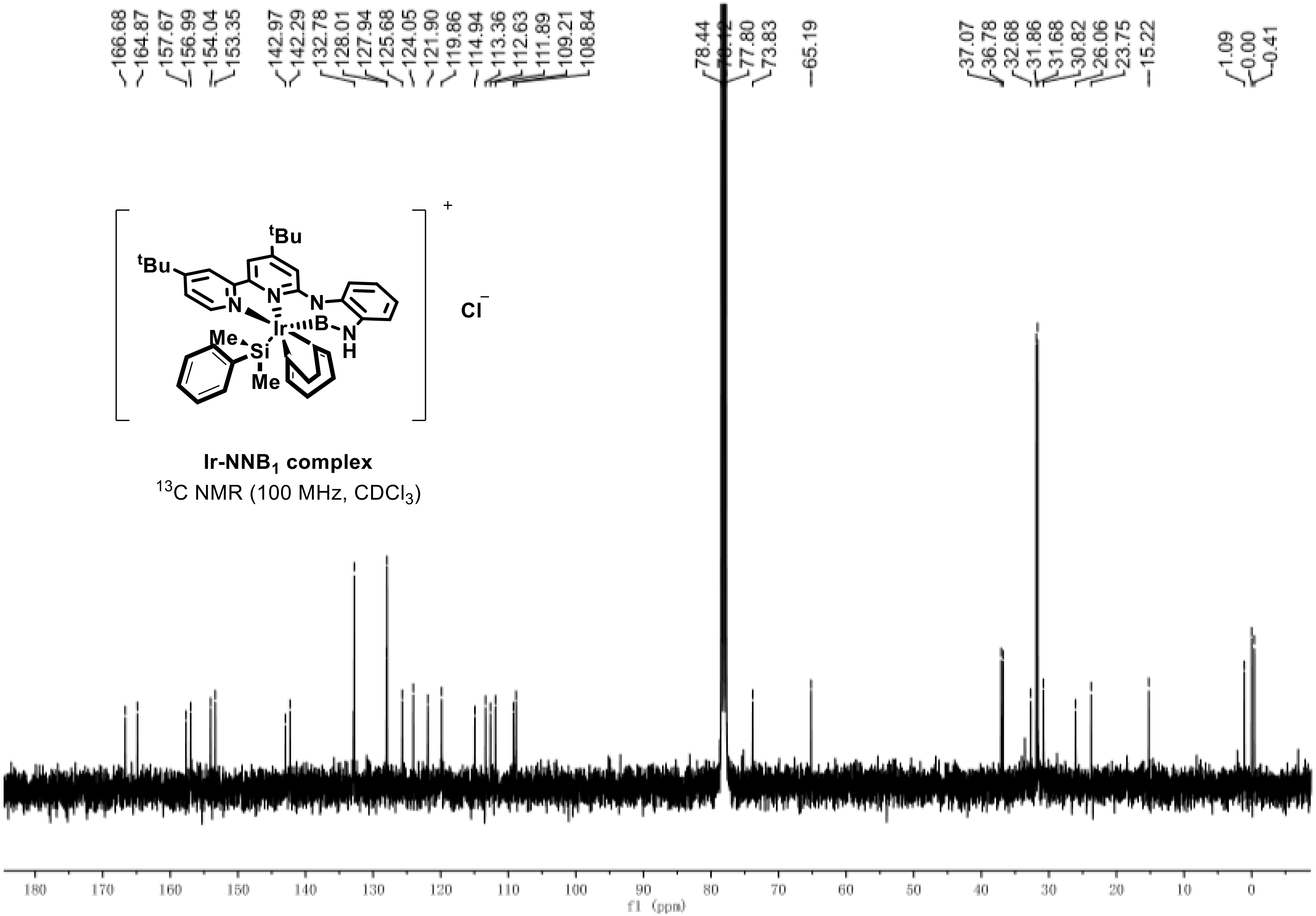

Ir-NNB$_1$ complex

^{13}C NMR (100 MHz, $CDCl_3$)

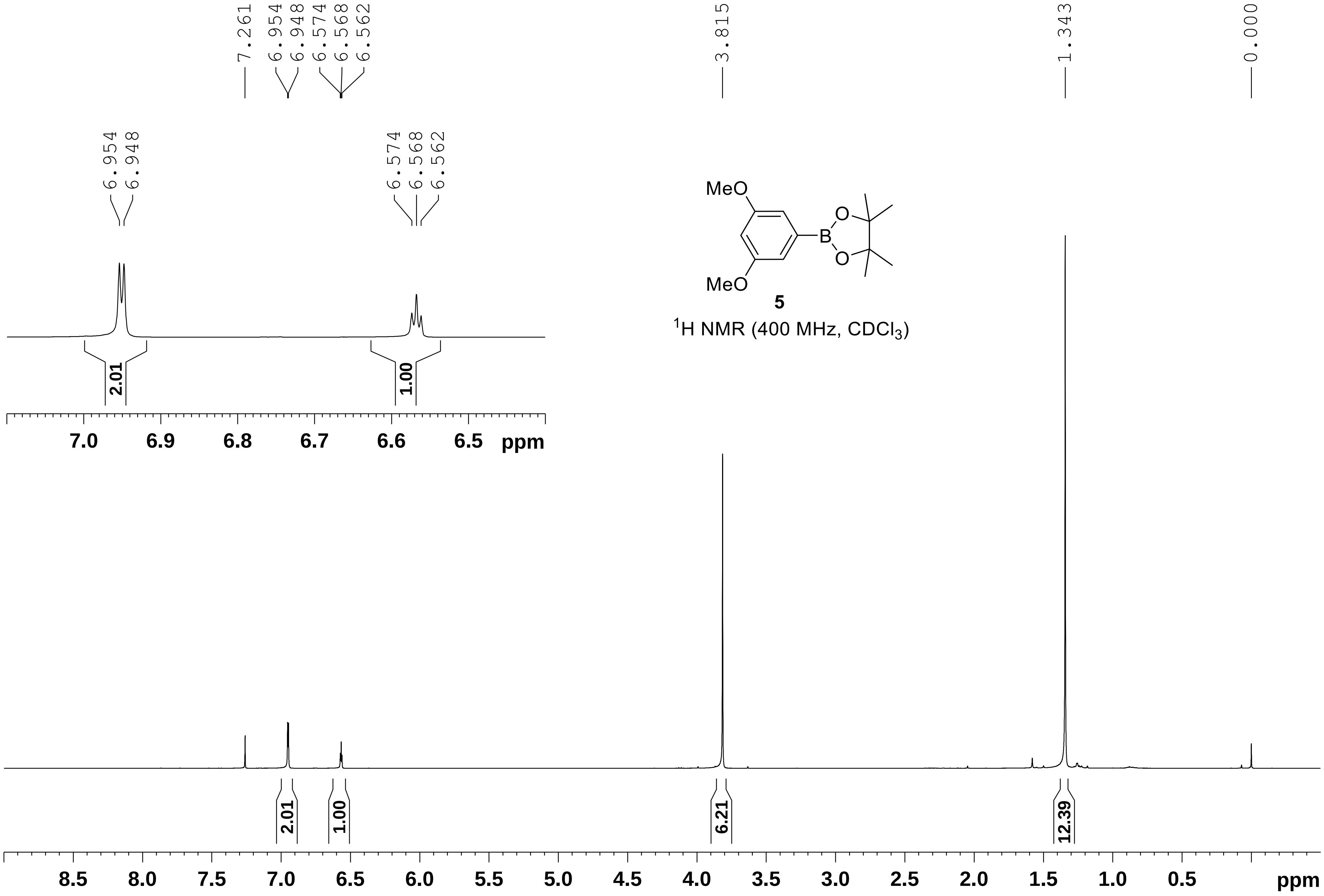
7.261
6.954
6.948
6.574
6.568
6.562
3.815
1.343
0.000
MeO
MeO
B
O
O
5
^{1}H NMR (400 MHz, $CDCl_3$)
2.01
1.00
6.21
12.39
7.0 6.9 6.8 6.7 6.6 6.5 ppm
8.5 8.0 7.5 7.0 6.5 6.0 5.5 5.0 4.5 4.0 3.5 3.0 2.5 2.0 1.5 1.0 0.5 ppm

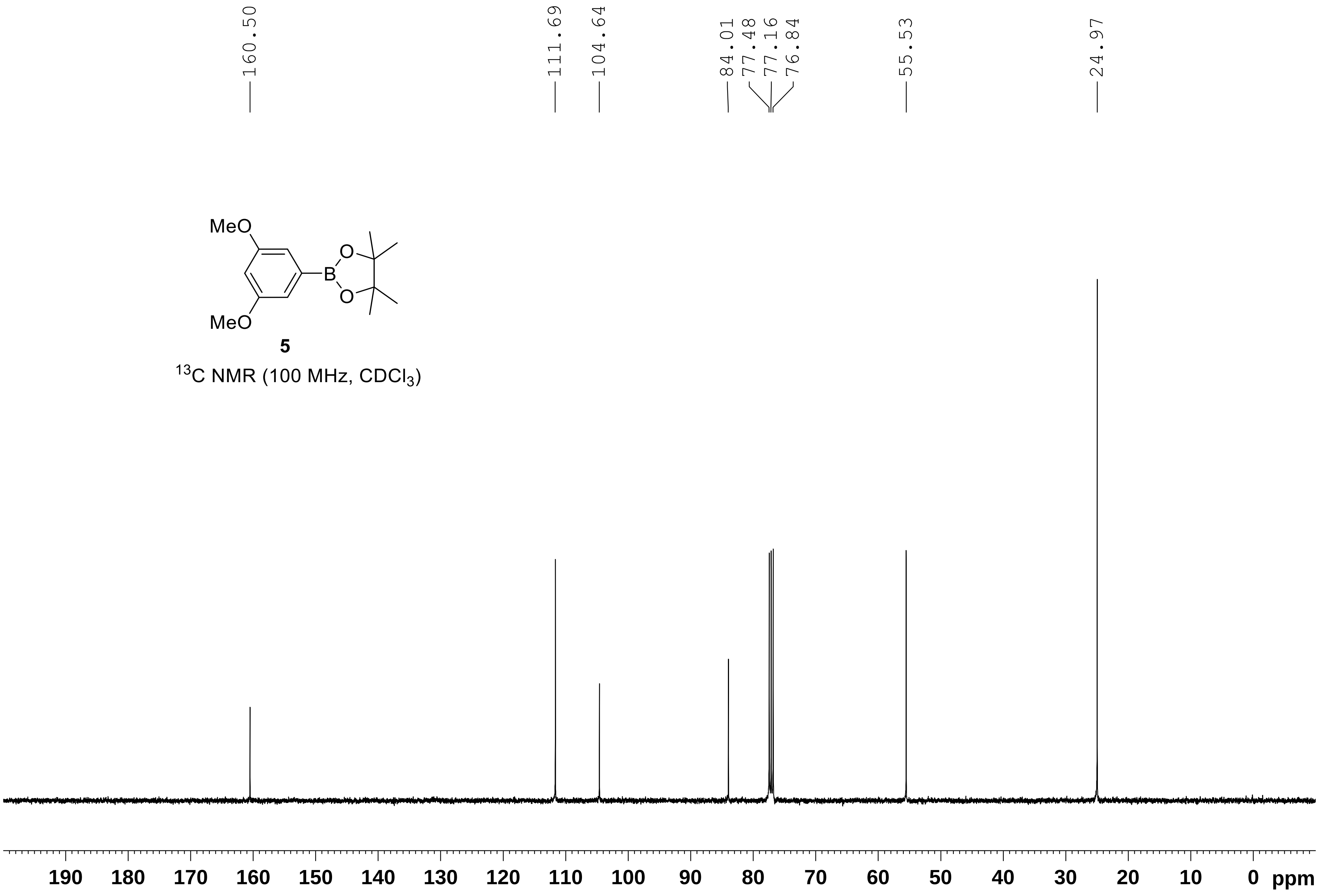
160.50
111.69
104.64
84.01
77.48
77.16
76.84
55.53
24.97
MeO
MeO
O
O
B
5
^{13}C NMR (100 MHz, $CDCl_3$)
190 180 170 160 150 140 130 120 110 100 90 80 70 60 50 40 30 20 10 0 ppm

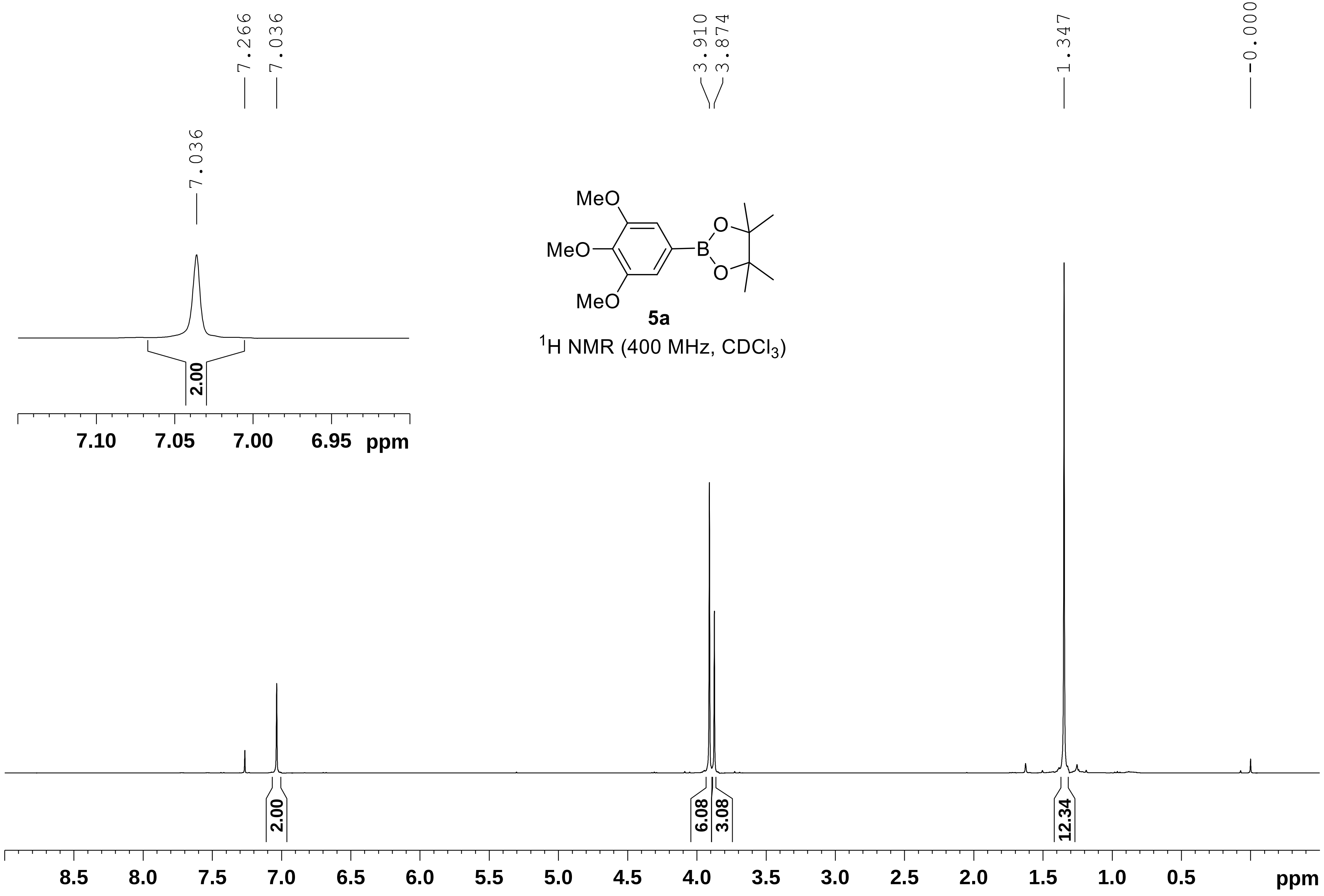
7.266
7.036
3.910
3.874
1.347
-0.000
7.036
2.00
7.10
7.05
7.00
6.95
ppm
MeO
MeO
MeO
B
O
O
5a
1H NMR (400 MHz, CDCl3)
2.00
6.08
3.08
12.34
8.5
8.0
7.5
7.0
6.5
6.0
5.5
5.0
4.5
4.0
3.5
3.0
2.5
2.0
1.5
1.0
0.5
ppm

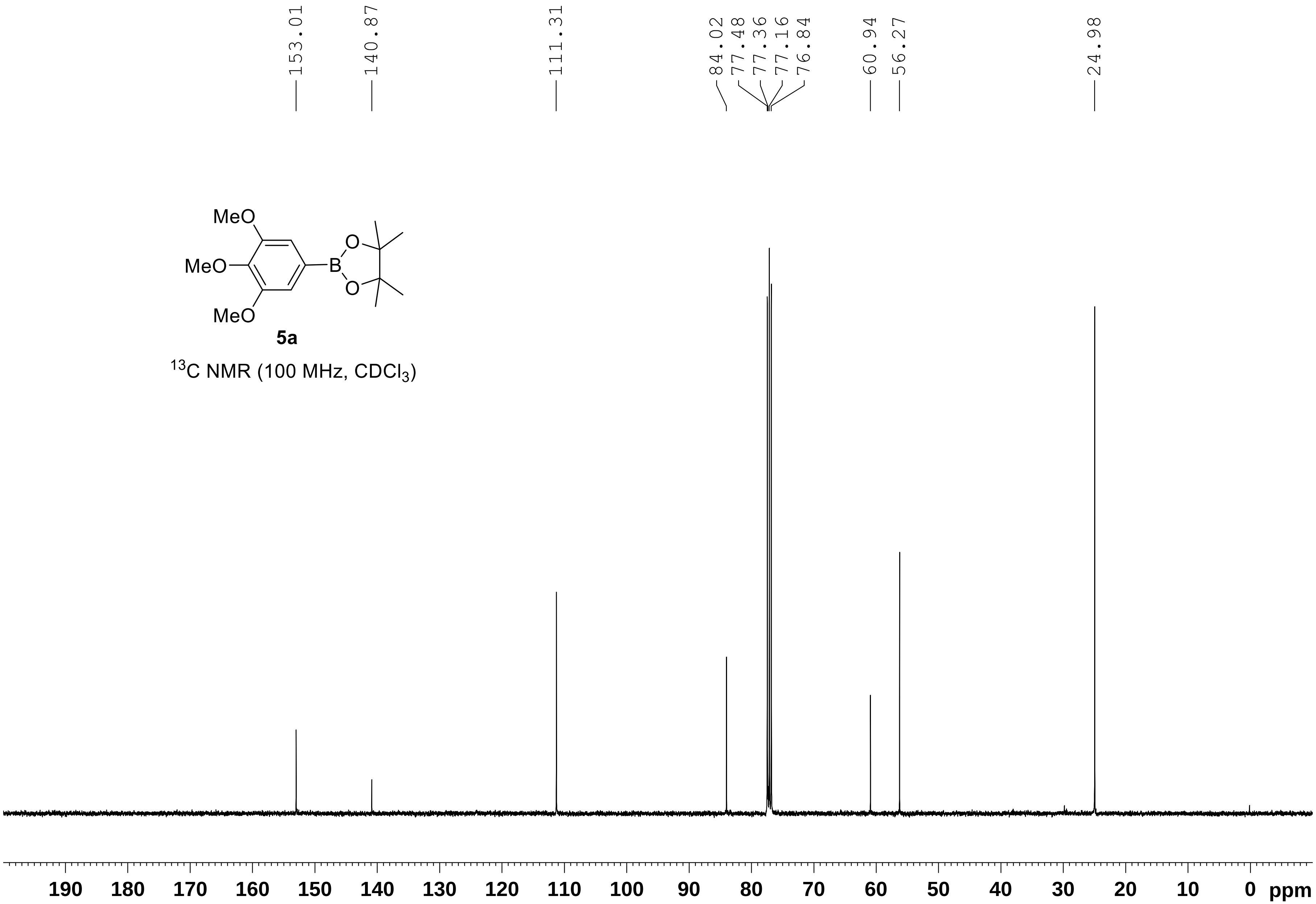

153.01
140.87
111.31
84.02
77.48
77.36
77.16
76.84
60.94
56.27
24.98
MeO
MeO
MeO
B
O
O
5a
^{13}C NMR (100 MHz, $CDCl_3$)
190 180 170 160 150 140 130 120 110 100 90 80 70 60 50 40 30 20 10 0 ppm

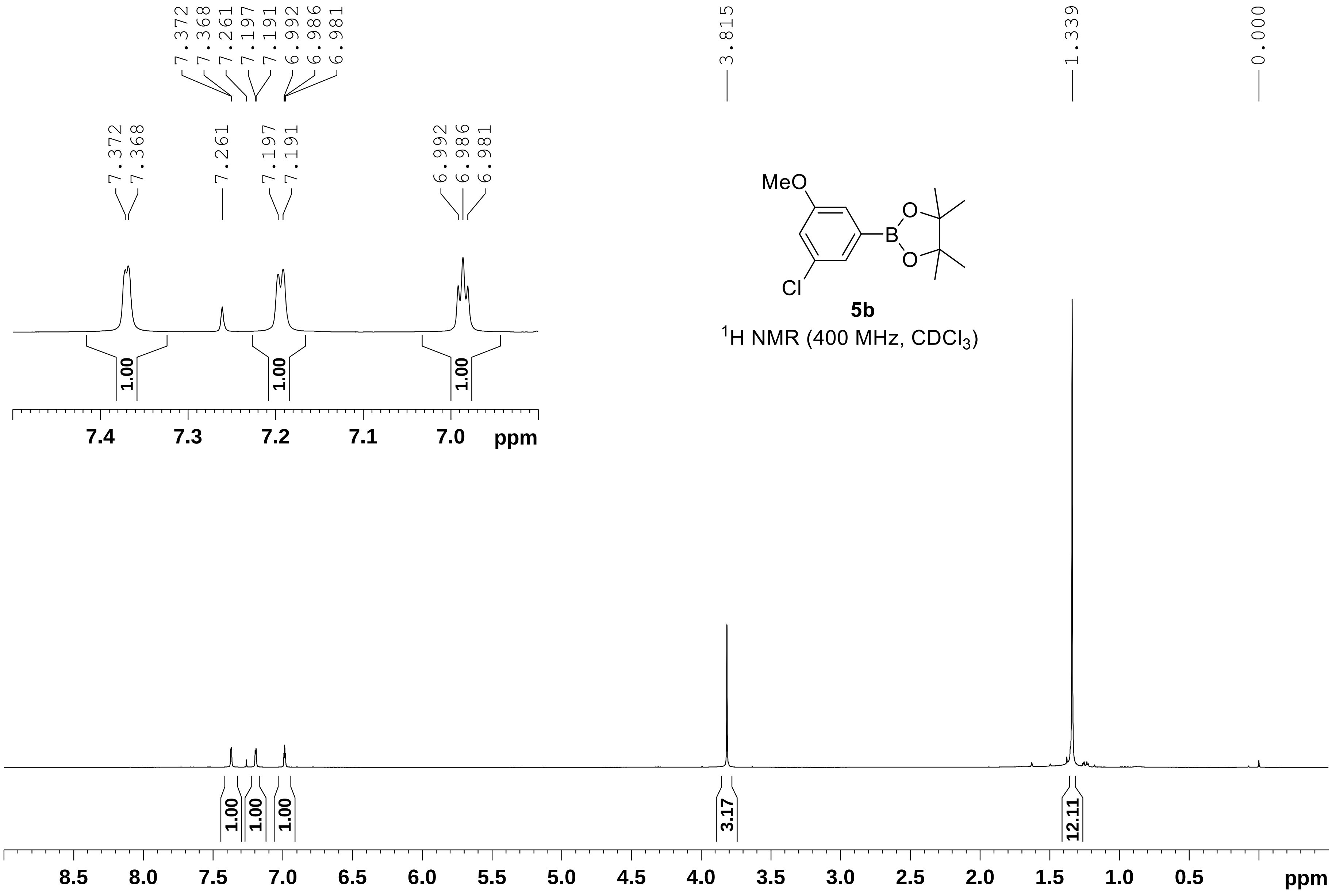
7.372
7.368
7.261
7.197
7.191
6.992
6.986
6.981
3.815
1.339
0.000
MeO
Cl
B
O
O
5b
¹H NMR (400 MHz, CDCl₃)
1.00
1.00
1.00
3.17
12.11
ppm

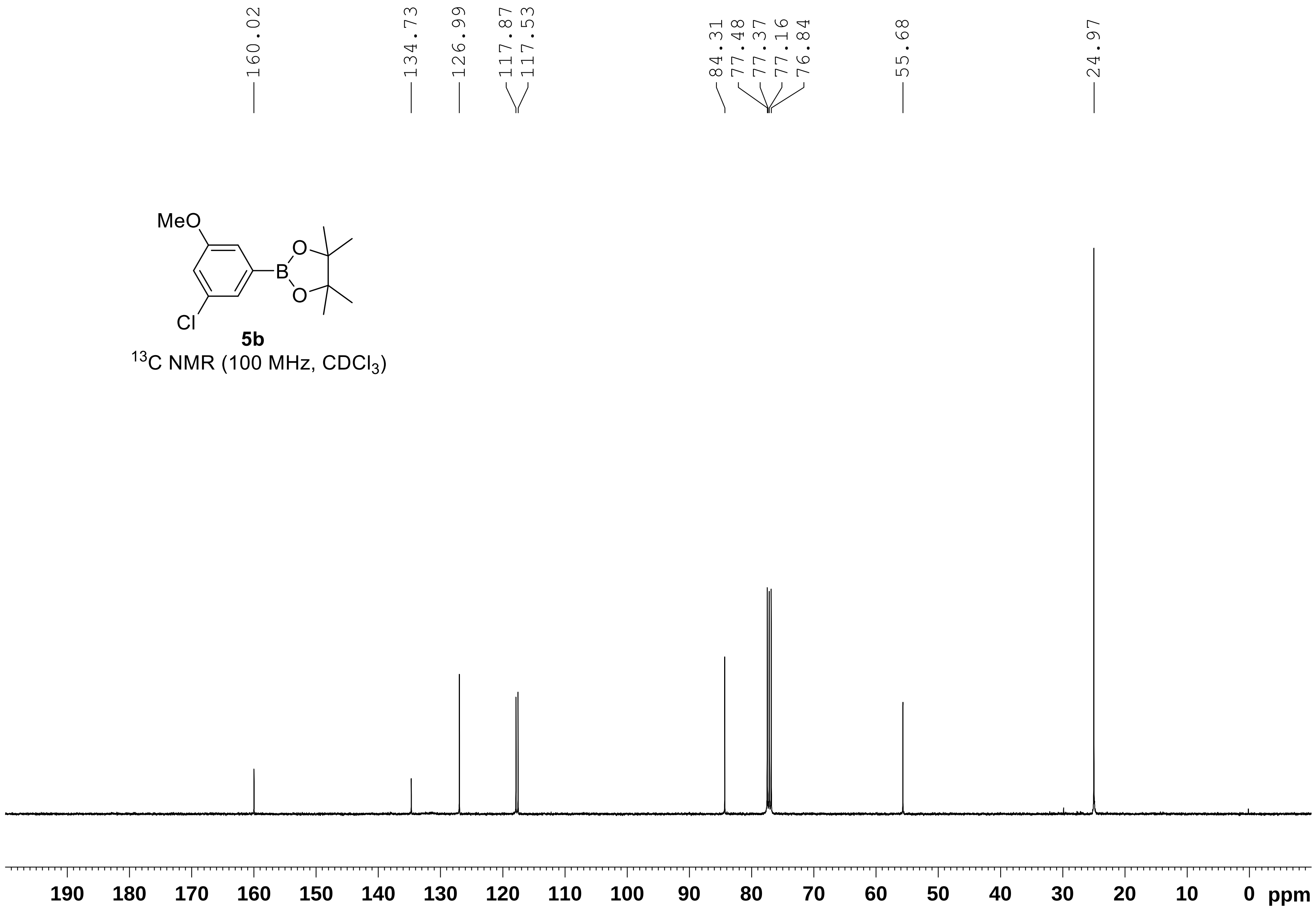
160.02
134.73
126.99
117.87
117.53
84.31
77.48
77.37
77.16
76.84
55.68
24.97
MeO
O
B
O
Cl
5b
13C NMR (100 MHz, CDCl3)
190 180 170 160 150 140 130 120 110 100 90 80 70 60 50 40 30 20 10 0 ppm

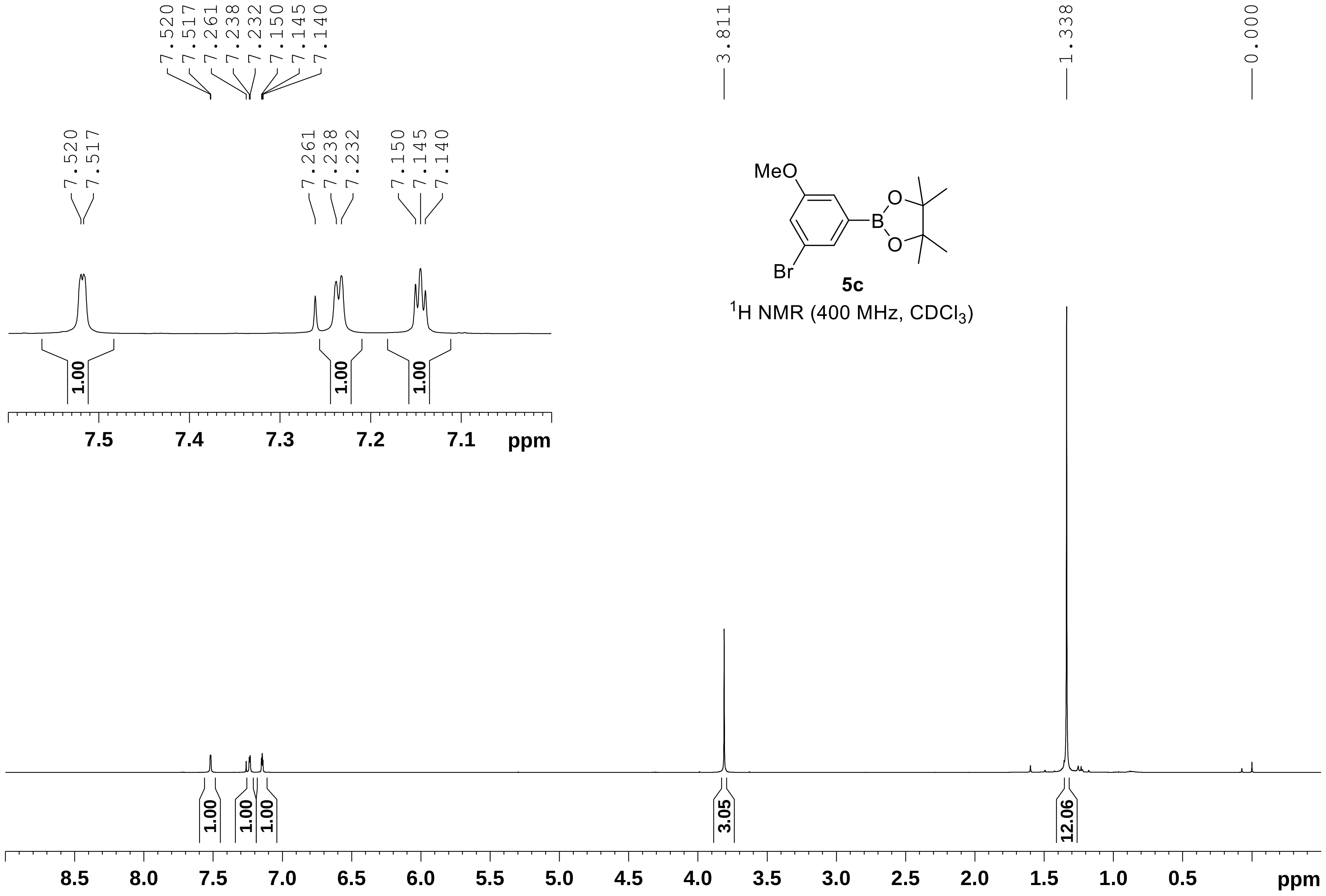

7.520
7.517
7.261
7.238
7.232
7.150
7.145
7.140
3.811
1.338
0.000
MeO
Br
B
O
O
5c
1H NMR (400 MHz, CDCl3)
1.00
1.00
1.00
3.05
12.06
7.5
7.4
7.3
7.2
7.1
ppm
8.5
8.0
7.0
6.5
6.0
5.5
5.0
4.5
4.0
3.5
3.0
2.5
2.0
1.5
1.0
0.5

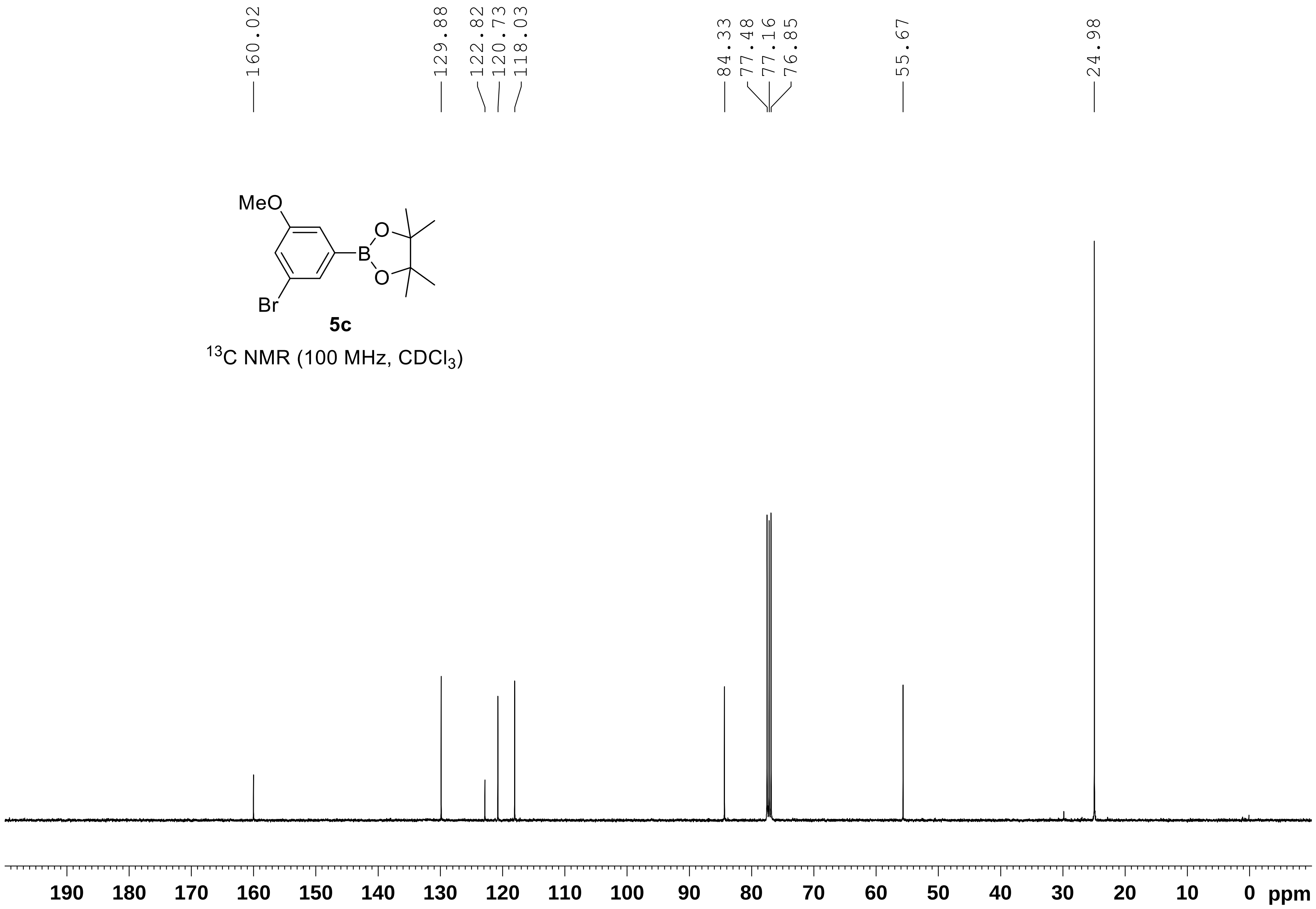
160.02
129.88
122.82
120.73
118.03
84.33
77.48
77.16
76.85
55.67
24.98
MeO
Br
O
B
O
5c
^{13}C NMR (100 MHz, $CDCl_3$)
190 180 170 160 150 140 130 120 110 100 90 80 70 60 50 40 30 20 10 0 ppm

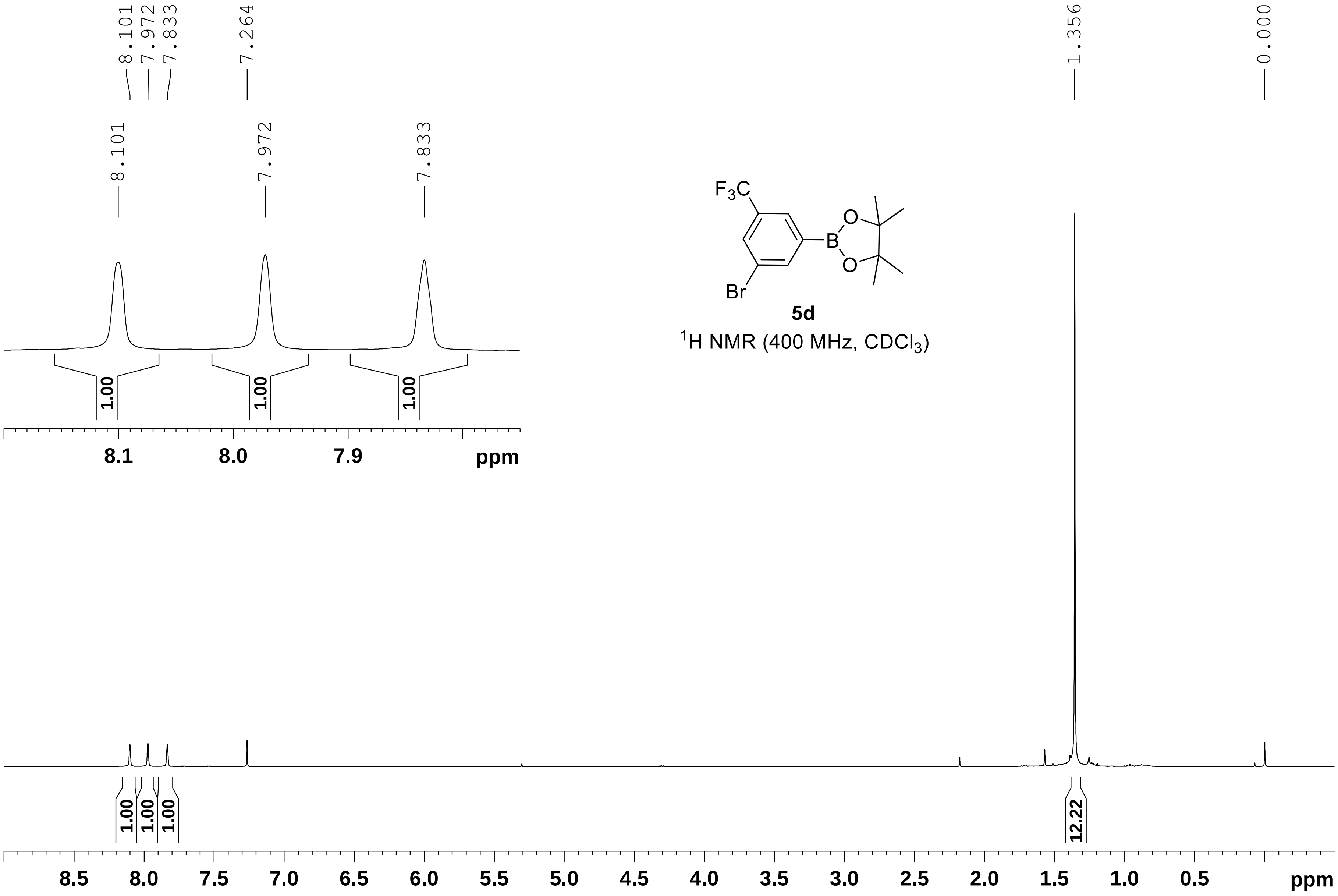
8.101
7.972
7.833
7.264
1.356
0.000
F_3C
B
O
O
Br
5d
^{1}H NMR (400 MHz, $CDCl_3$)
1.00
1.00
1.00
12.22
8.5 8.0 7.5 7.0 6.5 6.0 5.5 5.0 4.5 4.0 3.5 3.0 2.5 2.0 1.5 1.0 0.5 ppm
8.1 8.0 7.9 ppm

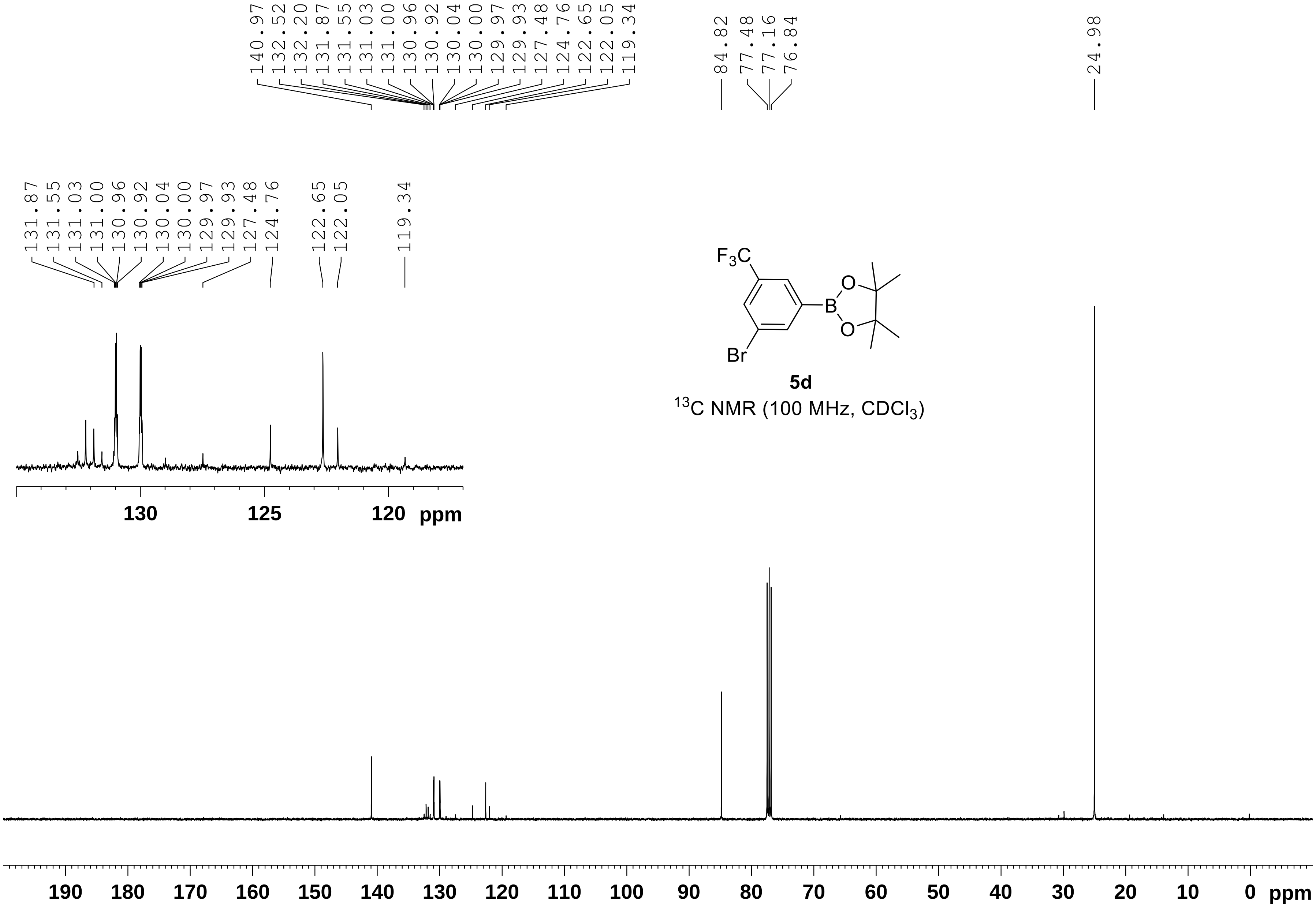

5d

^{13}C NMR (100 MHz, $CDCl_3$)

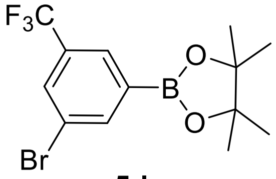

5d

^{19}F NMR (376.5 MHz, $CDCl_3$)

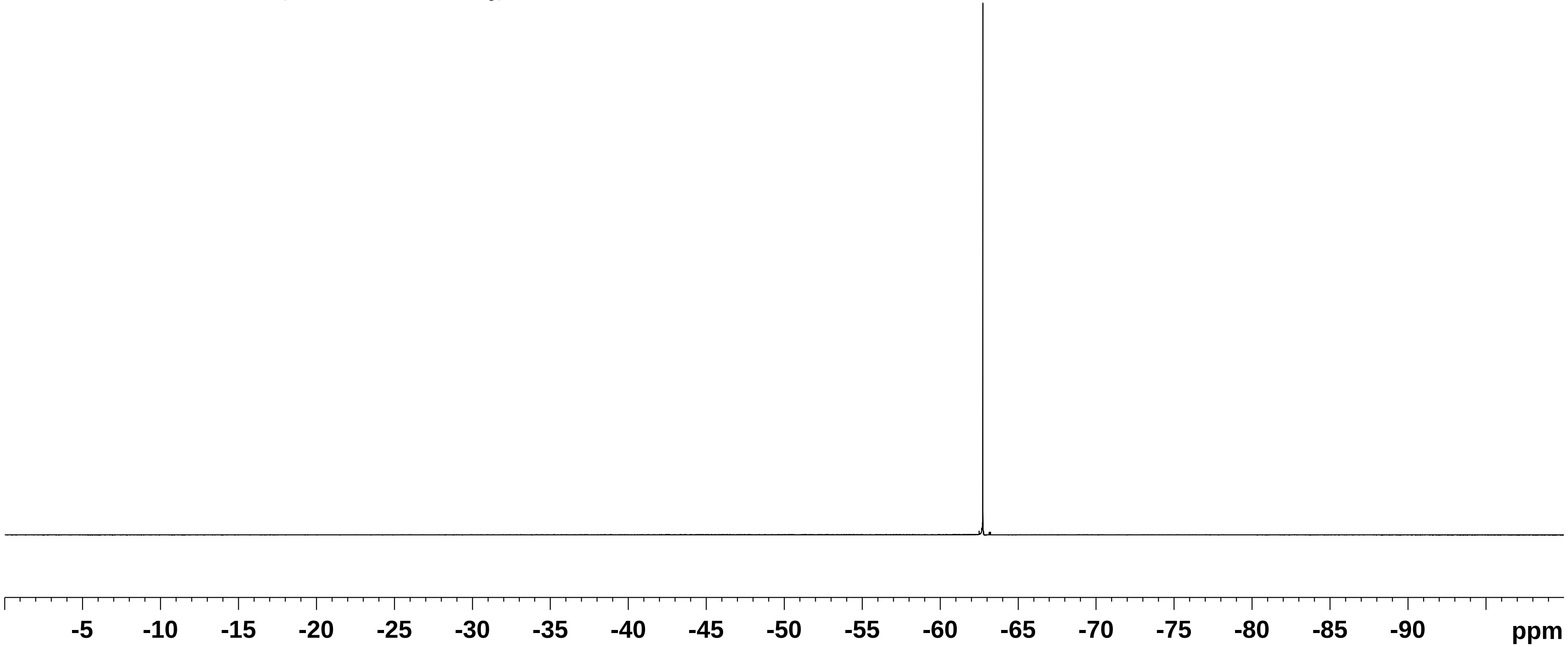

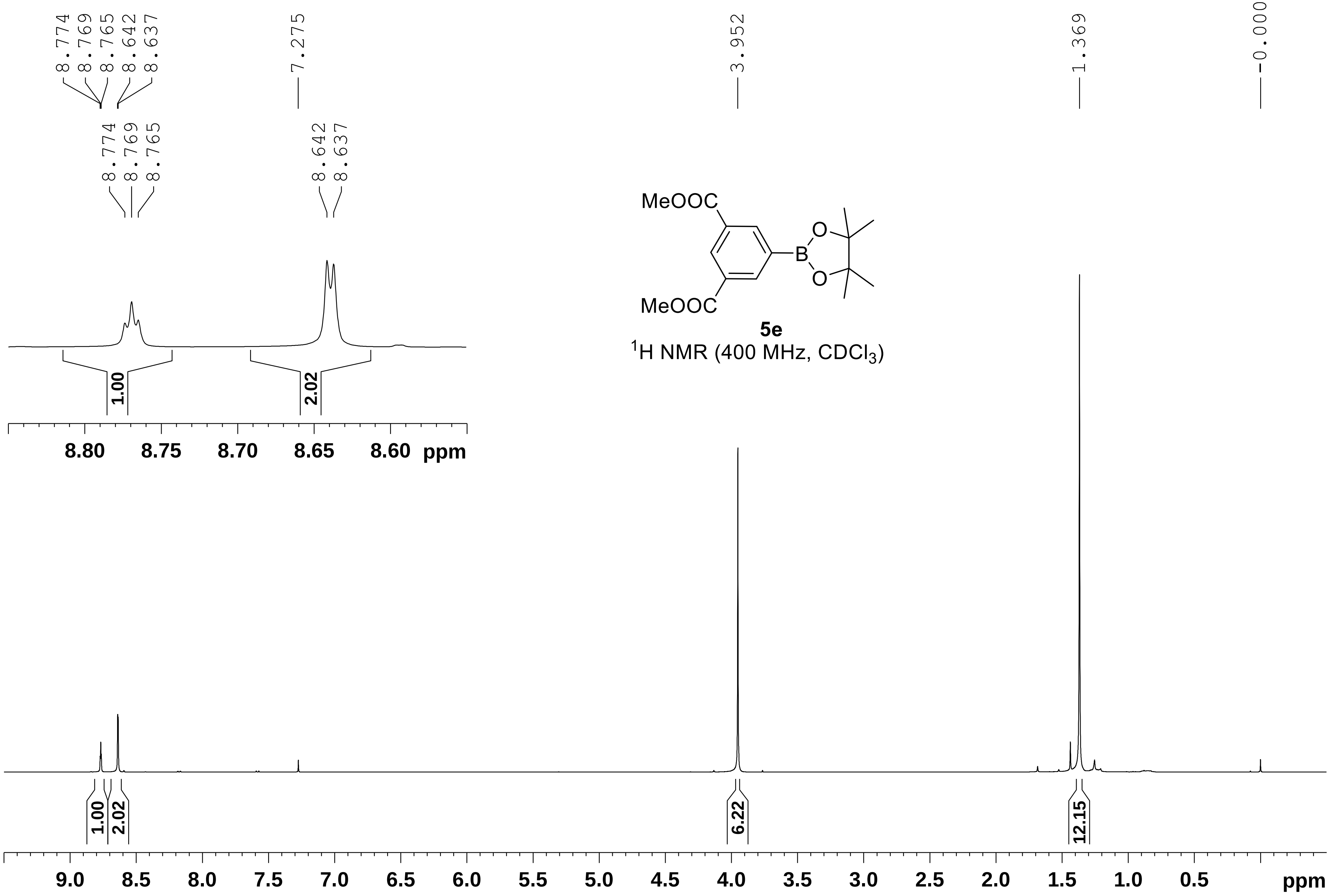
8.774
8.769
8.765
8.642
8.637
7.275
3.952
1.369
-0.000
MeOOC
MeOOC
B
O
O
5e
1H NMR (400 MHz, CDCl3)
1.00
2.02
8.80
8.75
8.70
8.65
8.60
ppm
6.22
12.15
9.0
8.5
8.0
7.5
7.0
6.5
6.0
5.5
5.0
4.5
4.0
3.5
3.0
2.5
2.0
1.5
1.0
0.5
ppm

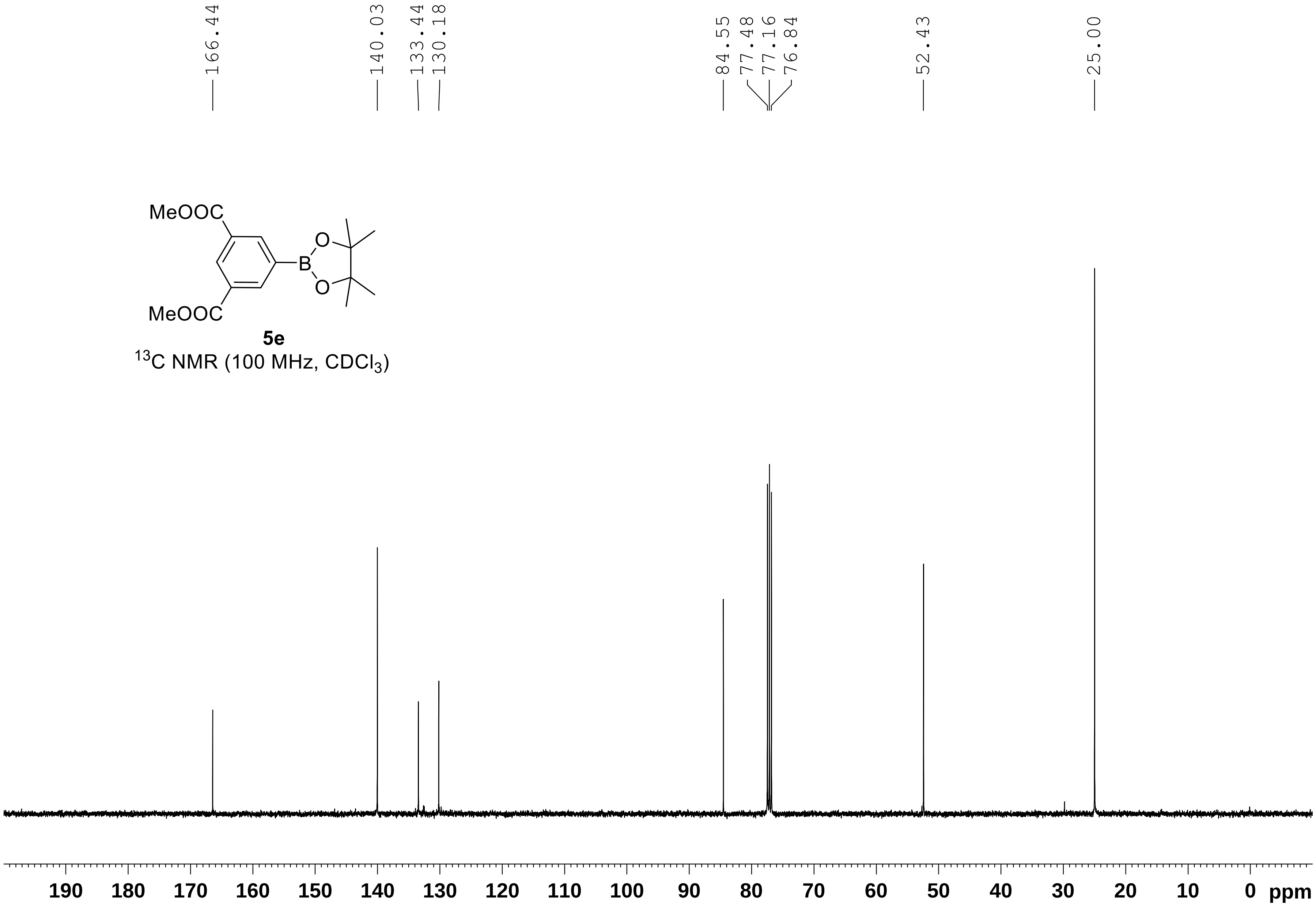
166.44
140.03
133.44
130.18
84.55
77.48
77.16
76.84
52.43
25.00
MeOOC
MeOOC
B
O
O
5e
13C NMR (100 MHz, CDCl3)
190
180
170
160
150
140
130
120
110
100
90
80
70
60
50
40
30
20
10
0
ppm

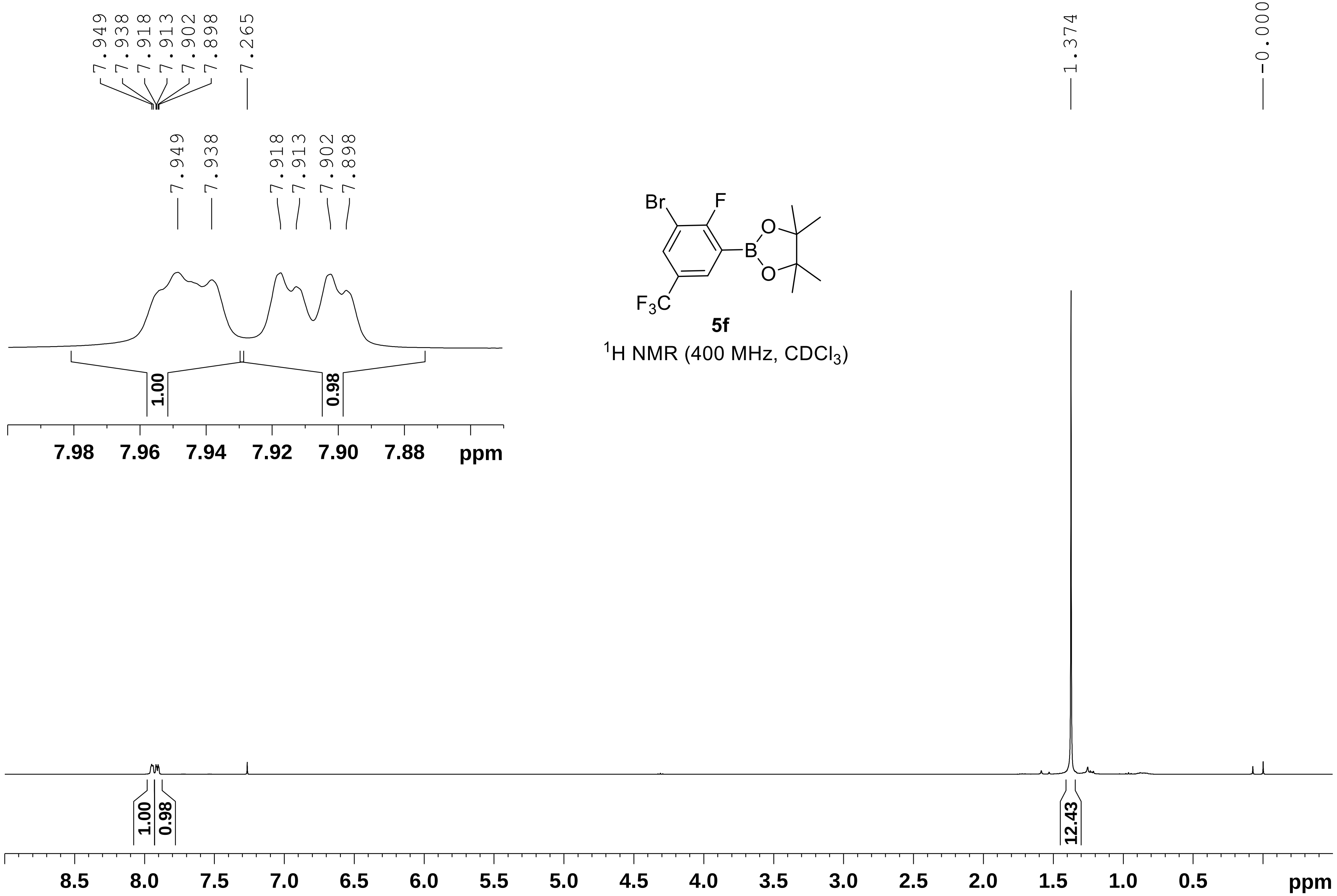
7.949
7.938
7.918
7.913
7.902
7.898
7.265
1.374
-0.000
Br
F
O
B
O
F3C
5f
1H NMR (400 MHz, CDCl3)
1.00
0.98
12.43
7.98 7.96 7.94 7.92 7.90 7.88 ppm
8.5 8.0 7.5 7.0 6.5 6.0 5.5 5.0 4.5 4.0 3.5 3.0 2.5 2.0 1.5 1.0 0.5 ppm

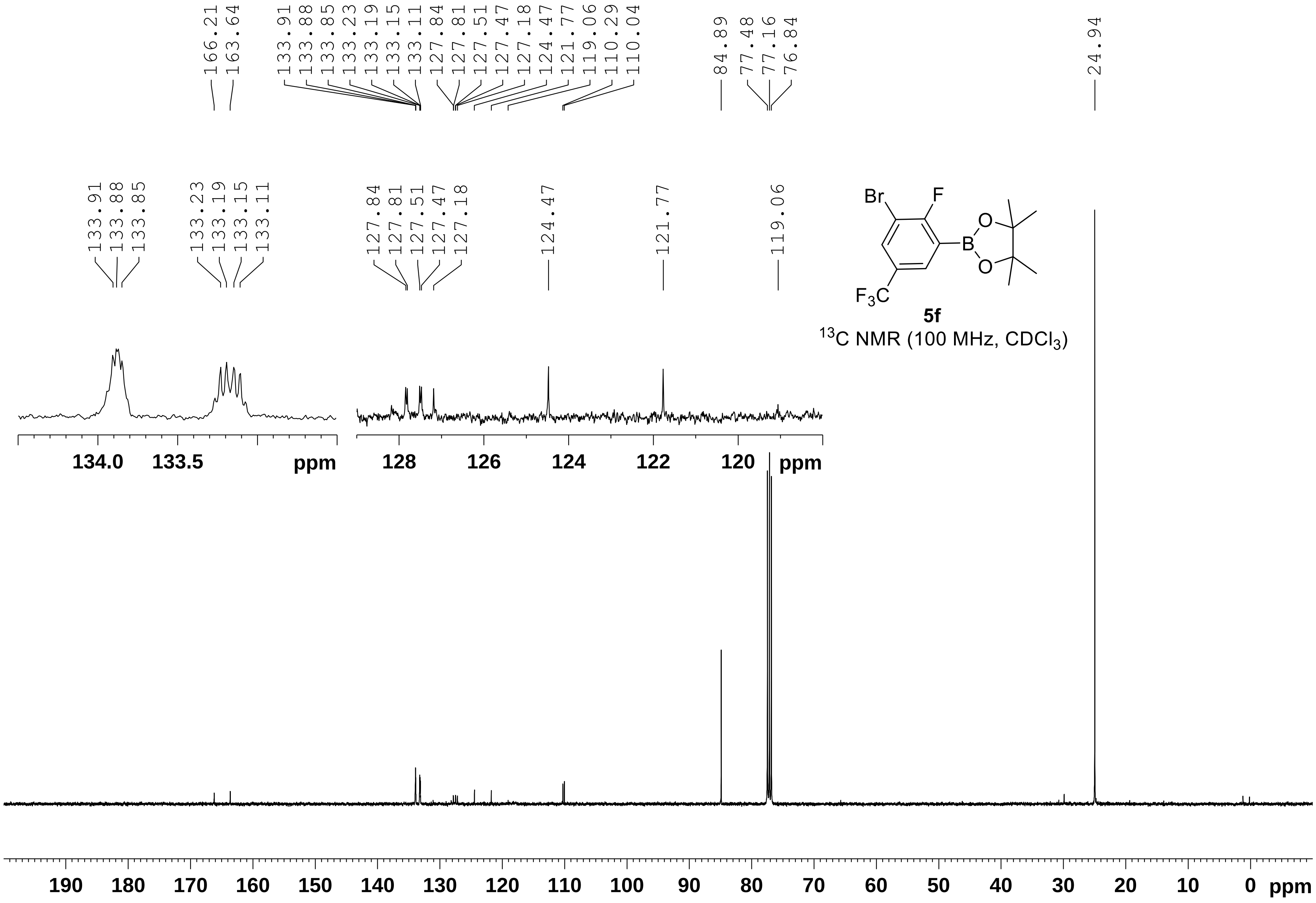
166.21
163.64
133.91
133.88
133.85
133.23
133.19
133.15
133.11
127.84
127.81
127.51
127.47
127.18
124.47
121.77
119.06
110.29
110.04
84.89
77.48
77.16
76.84
24.94
Br
F
O
B
O
F3C
5f
13C NMR (100 MHz, CDCl3)
134.0
133.5
ppm
128
126
124
122
120
ppm
190
180
170
160
150
140
130
120
110
100
90
80
70
60
50
40
30
20
10
0
ppm

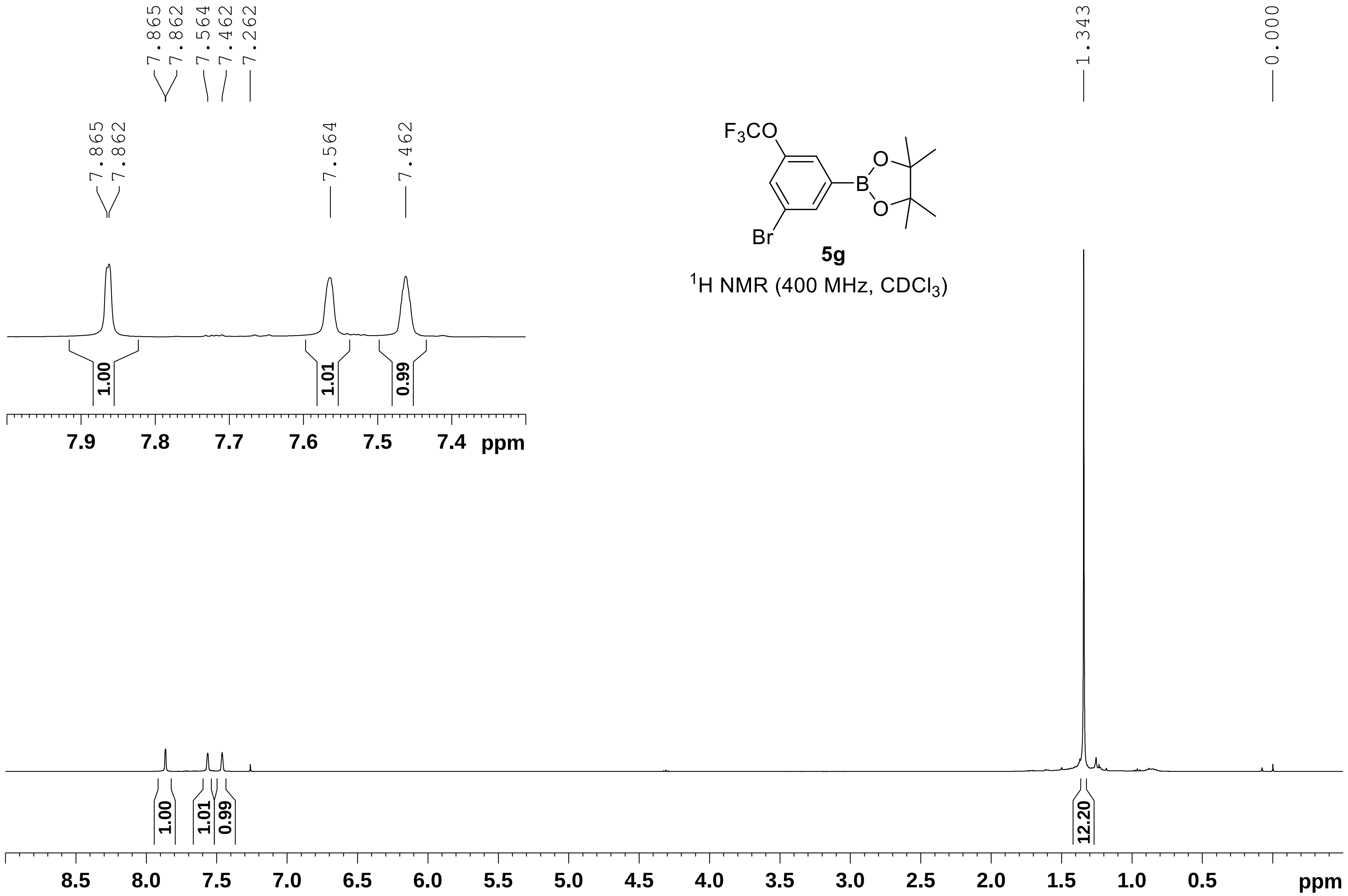
7.865
7.862
7.564
7.462
7.262
1.343
0.000
F3CO
Br
B
O
O
5g
1H NMR (400 MHz, CDCl3)
1.00
1.01
0.99
12.20
7.9 7.8 7.7 7.6 7.5 7.4 ppm
8.5 8.0 7.5 7.0 6.5 6.0 5.5 5.0 4.5 4.0 3.5 3.0 2.5 2.0 1.5 1.0 0.5 ppm

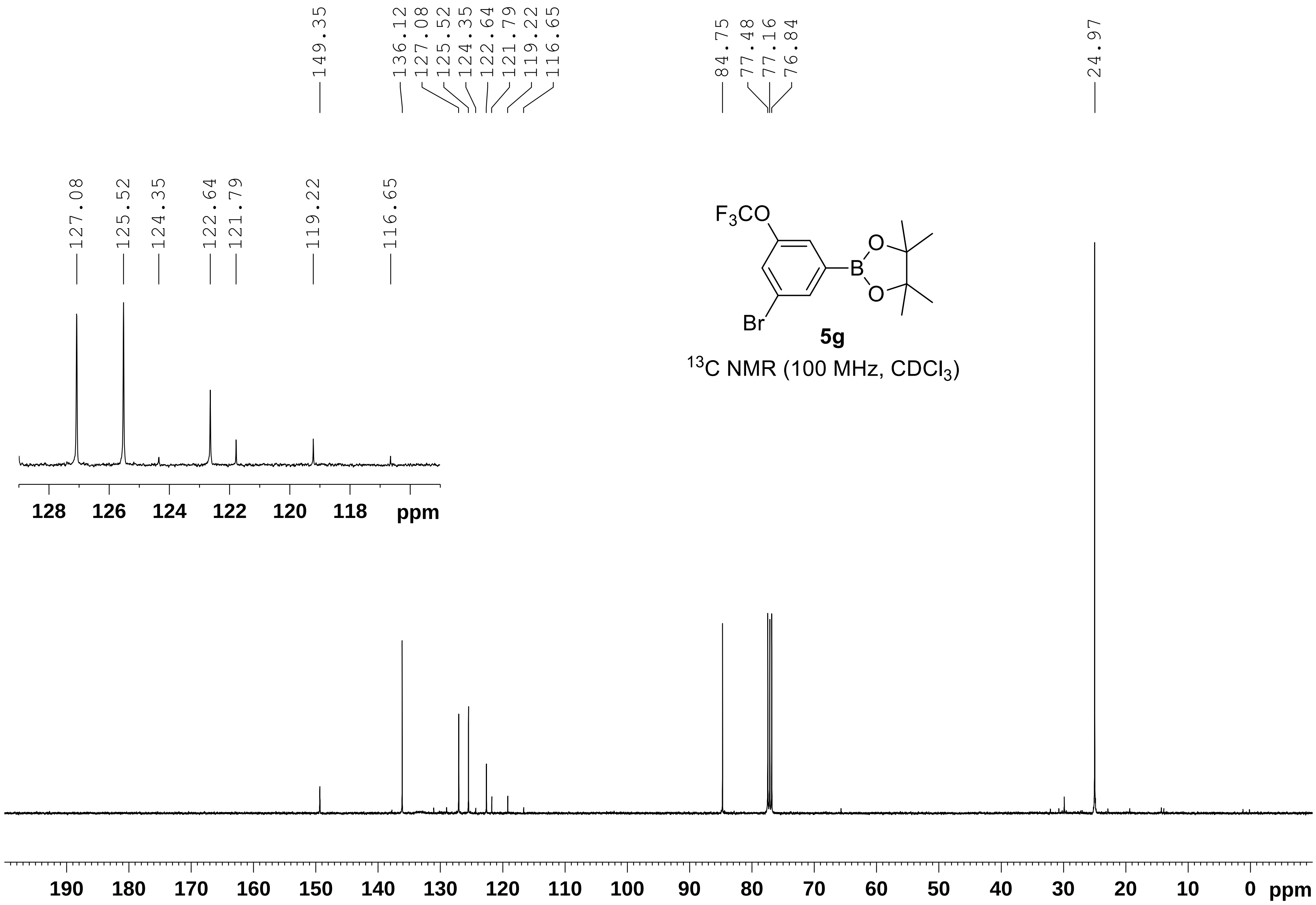
149.35
136.12
127.08
125.52
124.35
122.64
121.79
119.22
116.65
84.75
77.48
77.16
76.84
24.97
F3CO
O
B
O
Br
5g
13C NMR (100 MHz, CDCl3)
128 126 124 122 120 118 ppm
190 180 170 160 150 140 130 120 110 100 90 80 70 60 50 40 30 20 10 0 ppm

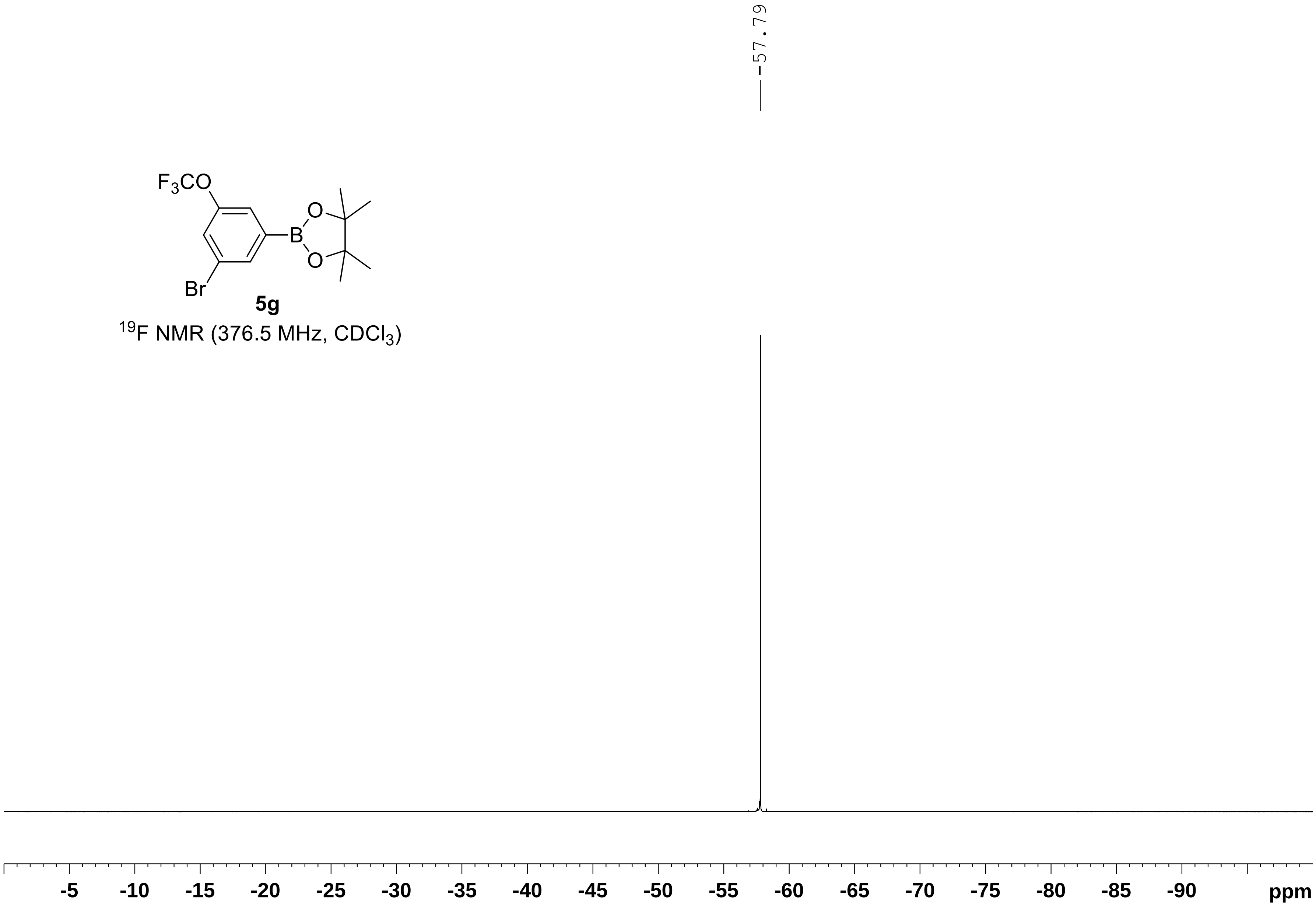

-57.79
F3CO
B
O
O
Br
5g
19F NMR (376.5 MHz, CDCl3)
-5 -10 -15 -20 -25 -30 -35 -40 -45 -50 -55 -60 -65 -70 -75 -80 -85 -90
ppm

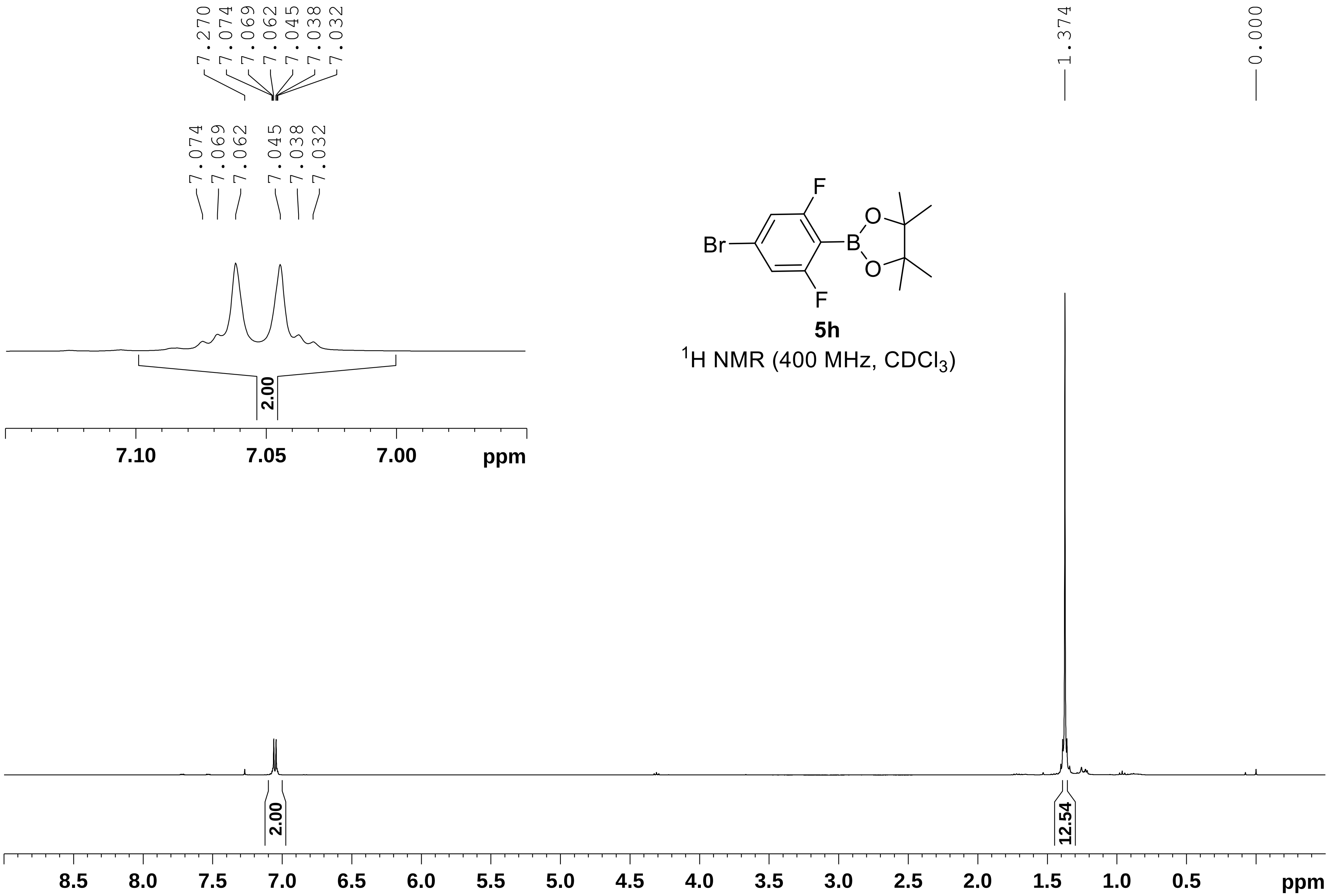
7.270
7.074
7.069
7.062
7.045
7.038
7.032
1.374
0.000
2.00
7.10
7.05
7.00
ppm
Br
F
F
B
O
O
5h
1H NMR (400 MHz, CDCl3)
2.00
12.54
8.5
8.0
7.5
7.0
6.5
6.0
5.5
5.0
4.5
4.0
3.5
3.0
2.5
2.0
1.5
1.0
0.5
ppm

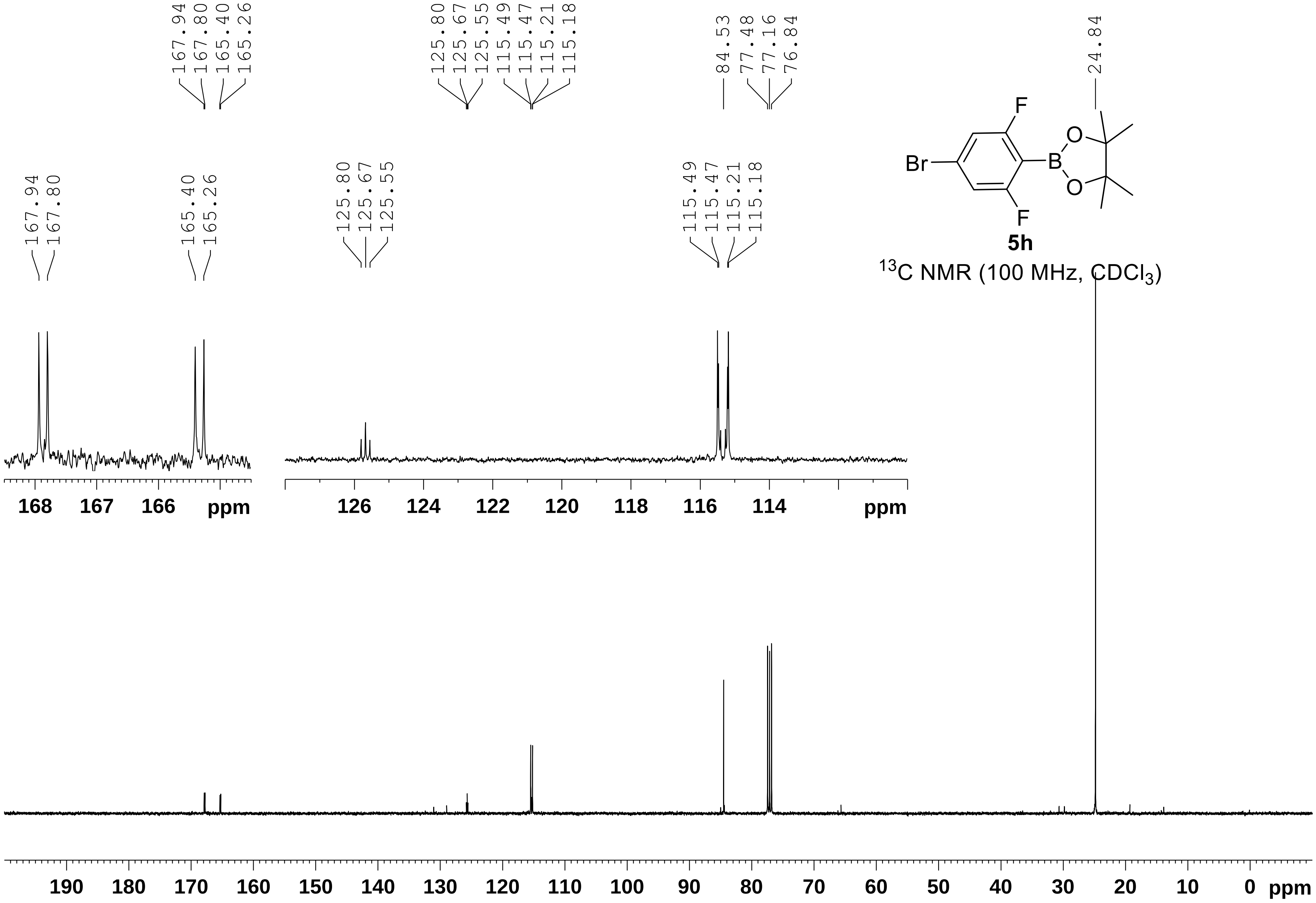

5h

^{13}C NMR (100 MHz, $CDCl_3$)

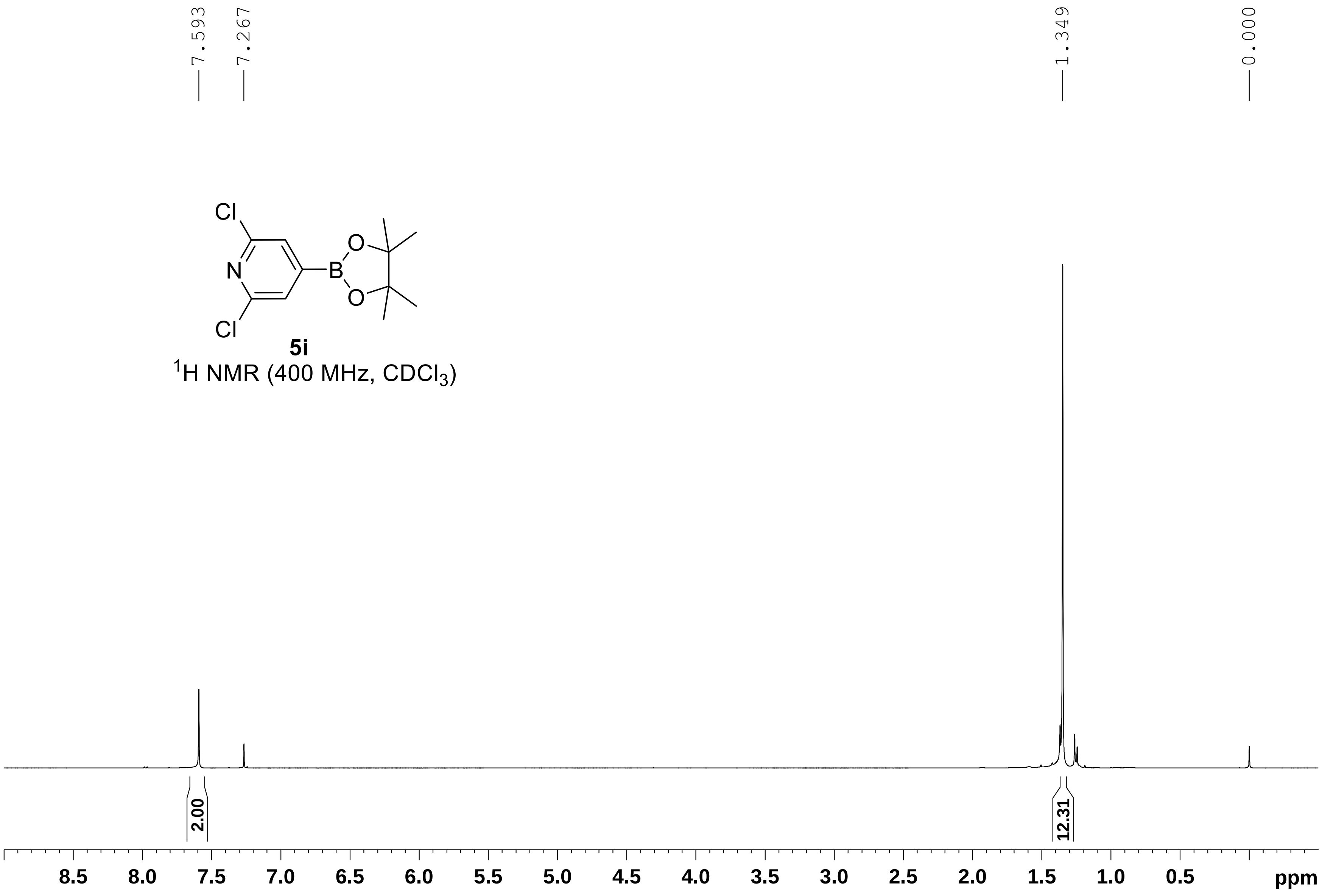
7.593
7.267
1.349
0.000
Cl
N
Cl
B
O
O
5i
1H NMR (400 MHz, CDCl3)
2.00
12.31
8.5
8.0
7.5
7.0
6.5
6.0
5.5
5.0
4.5
4.0
3.5
3.0
2.5
2.0
1.5
1.0
0.5
ppm

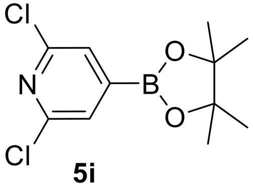

5i

^{13}C NMR (100 MHz, $CDCl_3$)

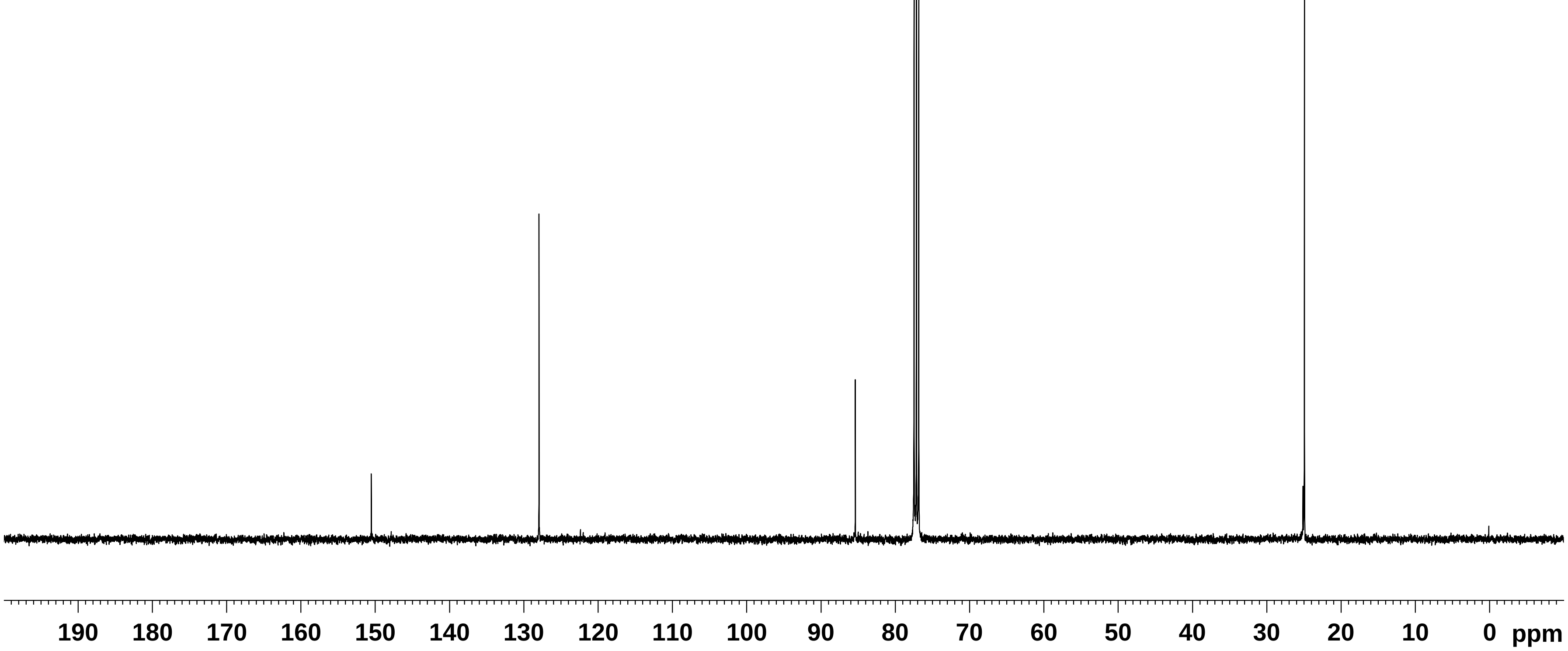

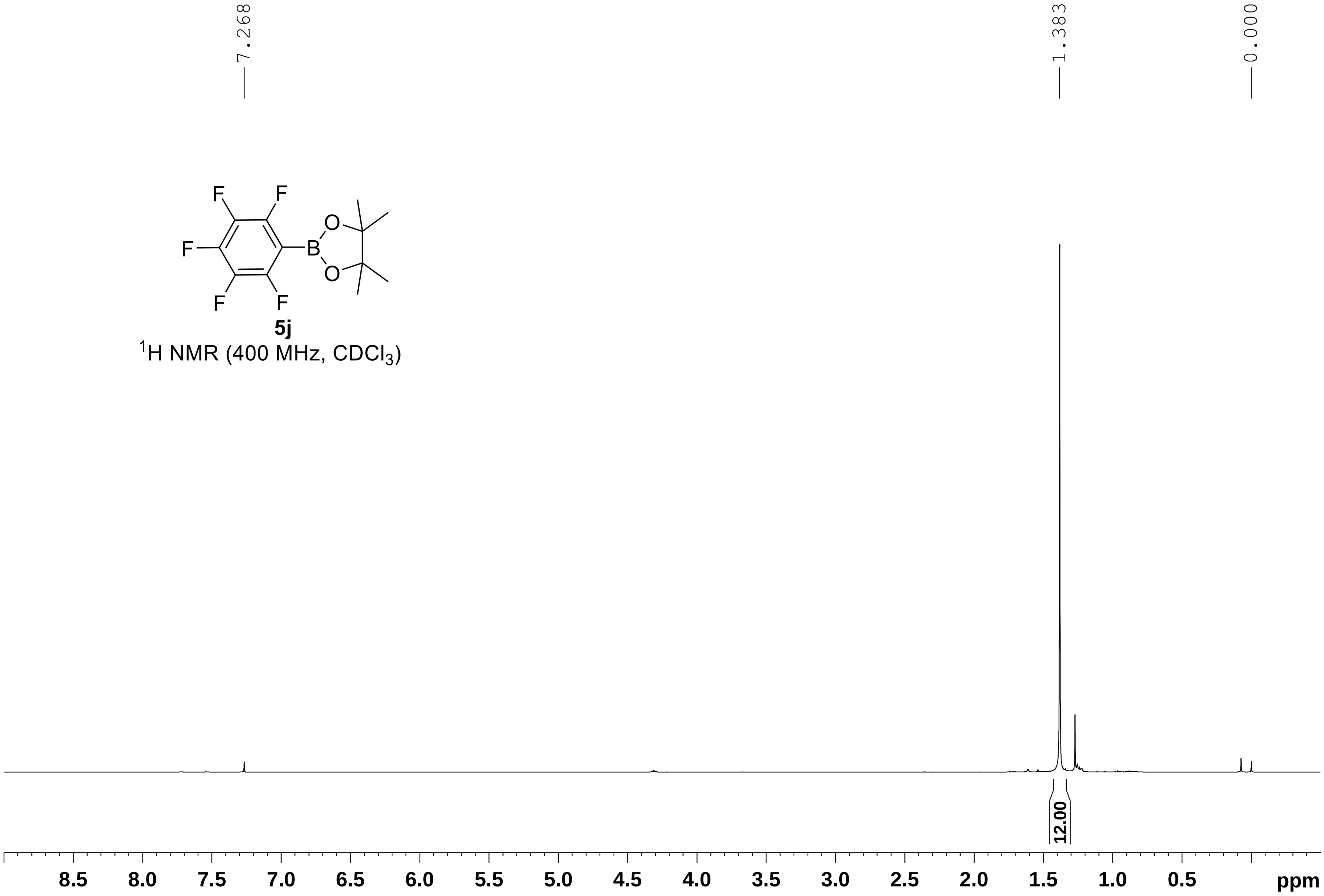
7.268
1.383
0.000
F
F
F
B
O
O
F
F
5j
¹H NMR (400 MHz, CDCl₃)
12.00
8.5
8.0
7.5
7.0
6.5
6.0
5.5
5.0
4.5
4.0
3.5
3.0
2.5
2.0
1.5
1.0
0.5
ppm

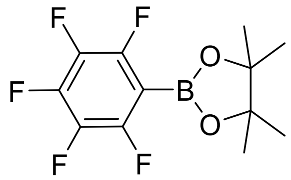

5j

^{13}C NMR (100 MHz, $CDCl_3$)

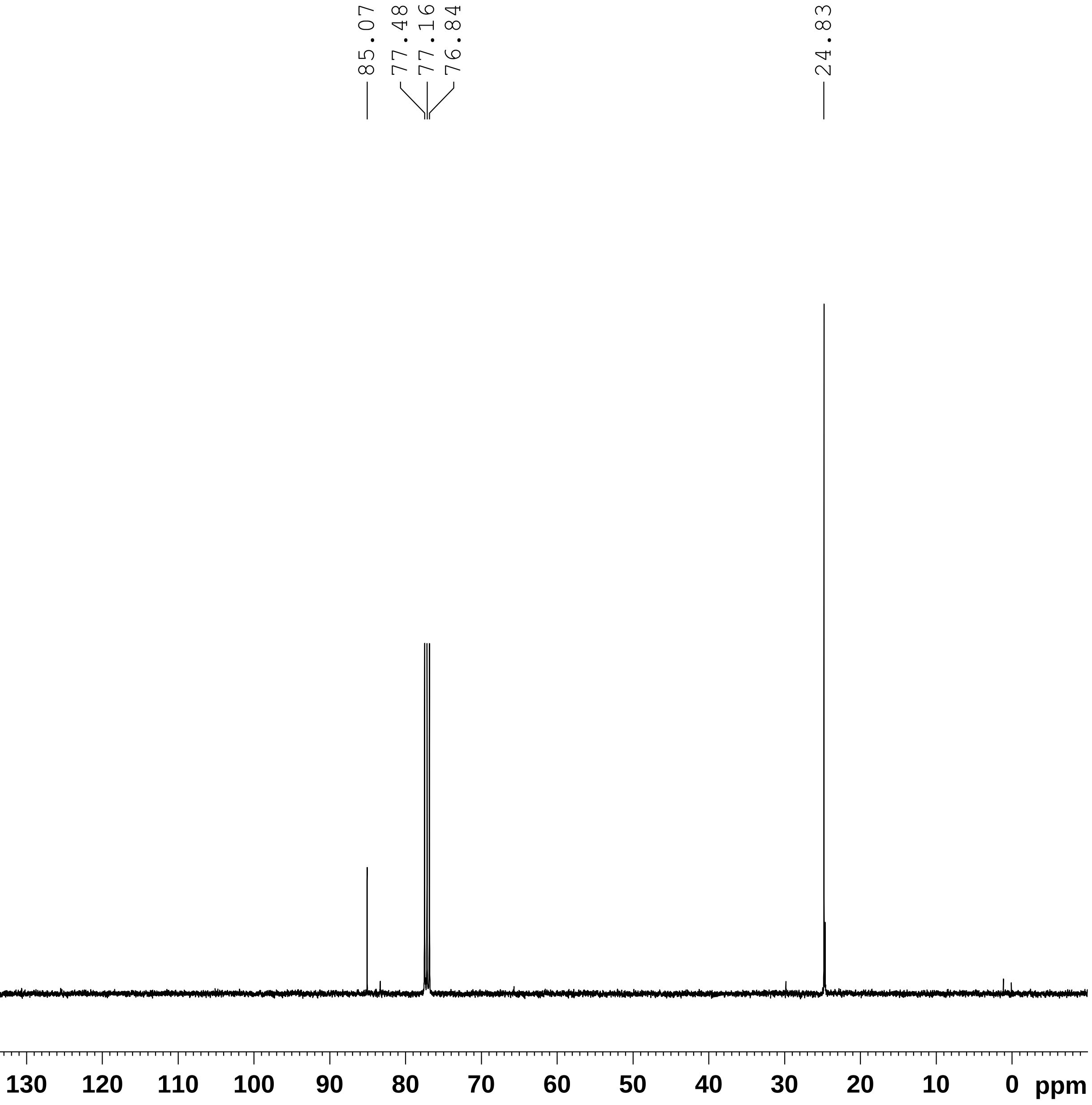

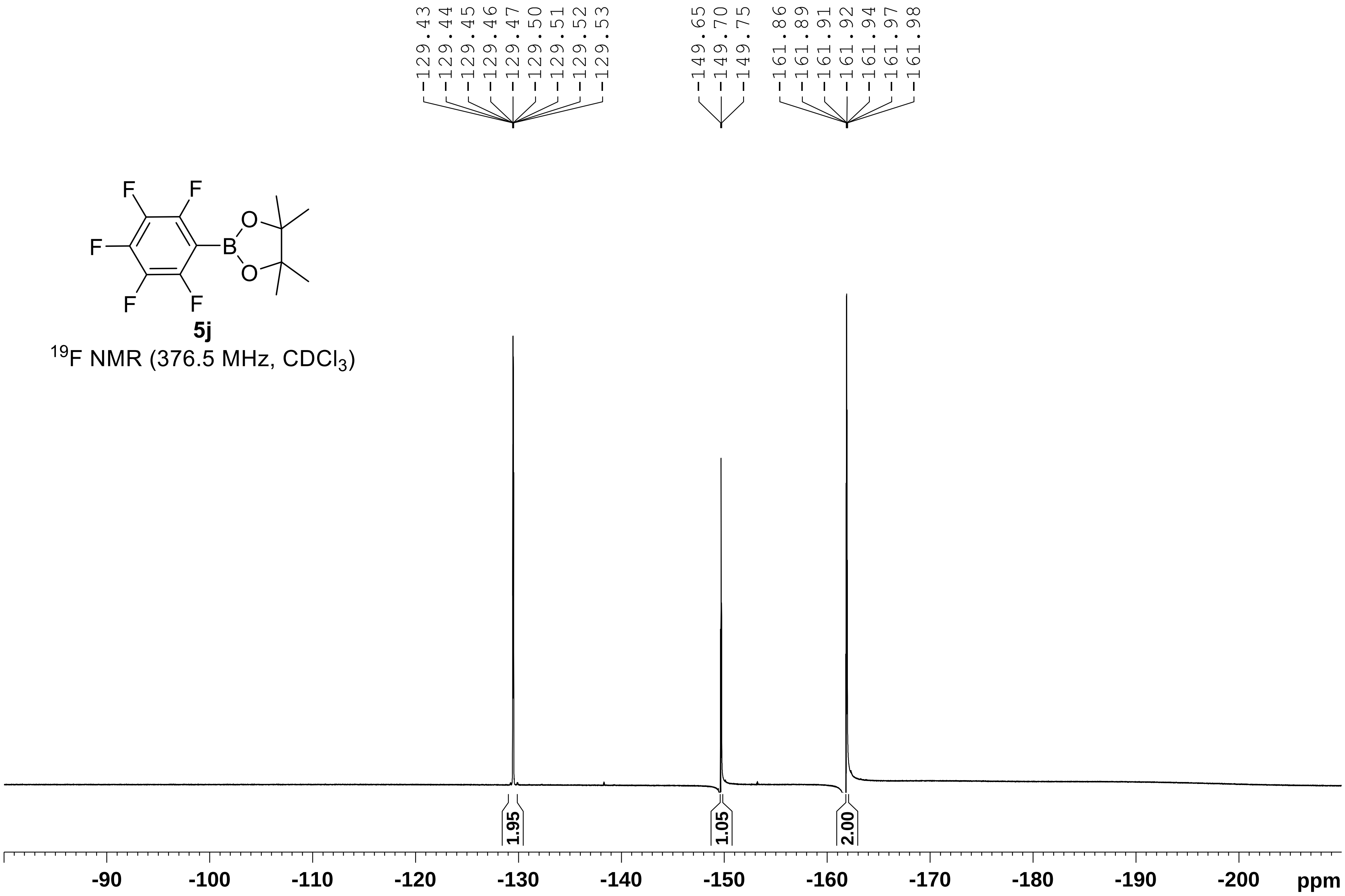

^{19}F NMR (376.5 MHz, $CDCl_3$)

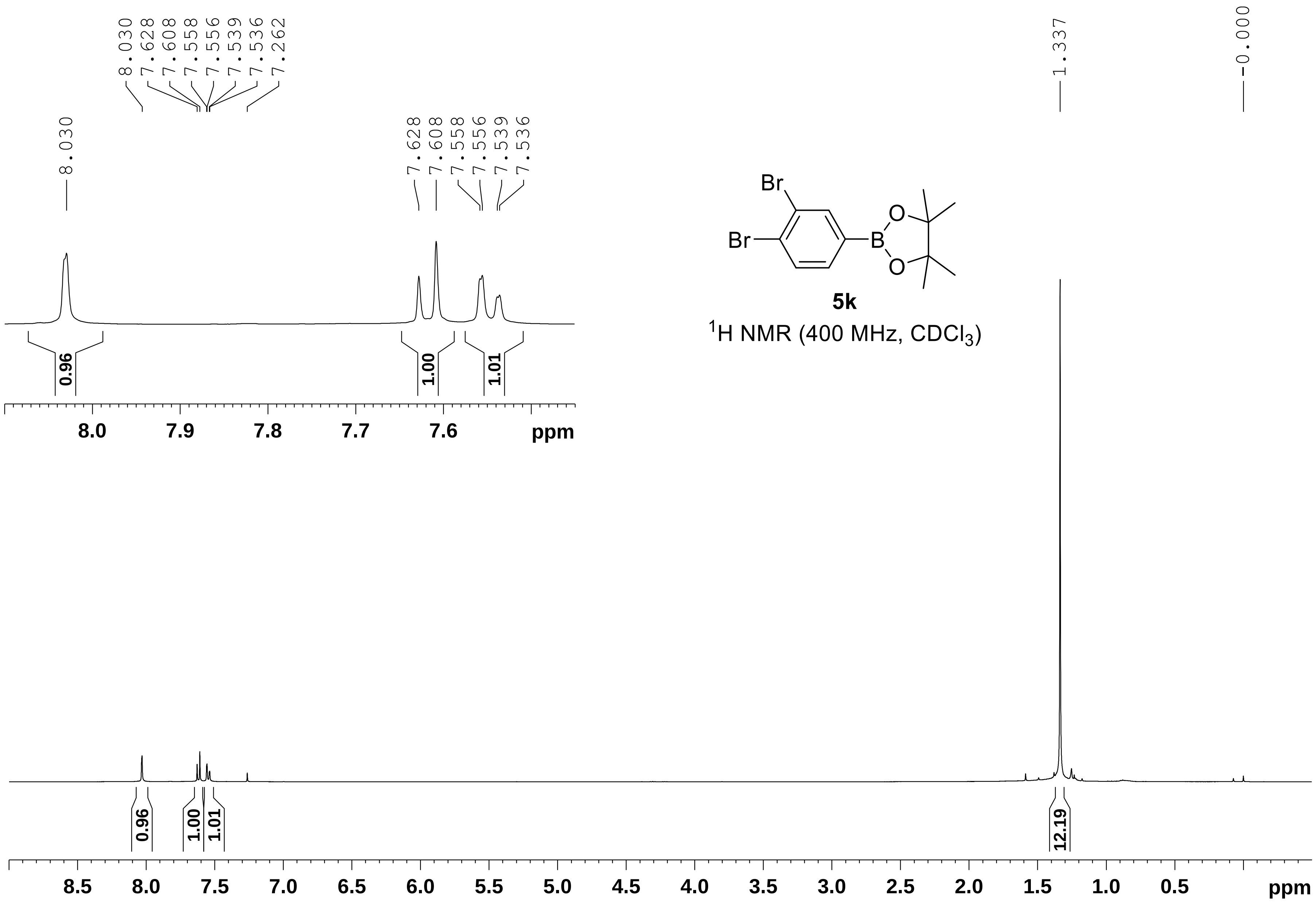
8.030
7.628
7.608
7.558
7.556
7.539
7.536
7.262
1.337
-0.000
Br
Br
B
O
O
5k
^{1}H NMR (400 MHz, $CDCl_3$)
0.96
1.00
1.01
12.19
ppm

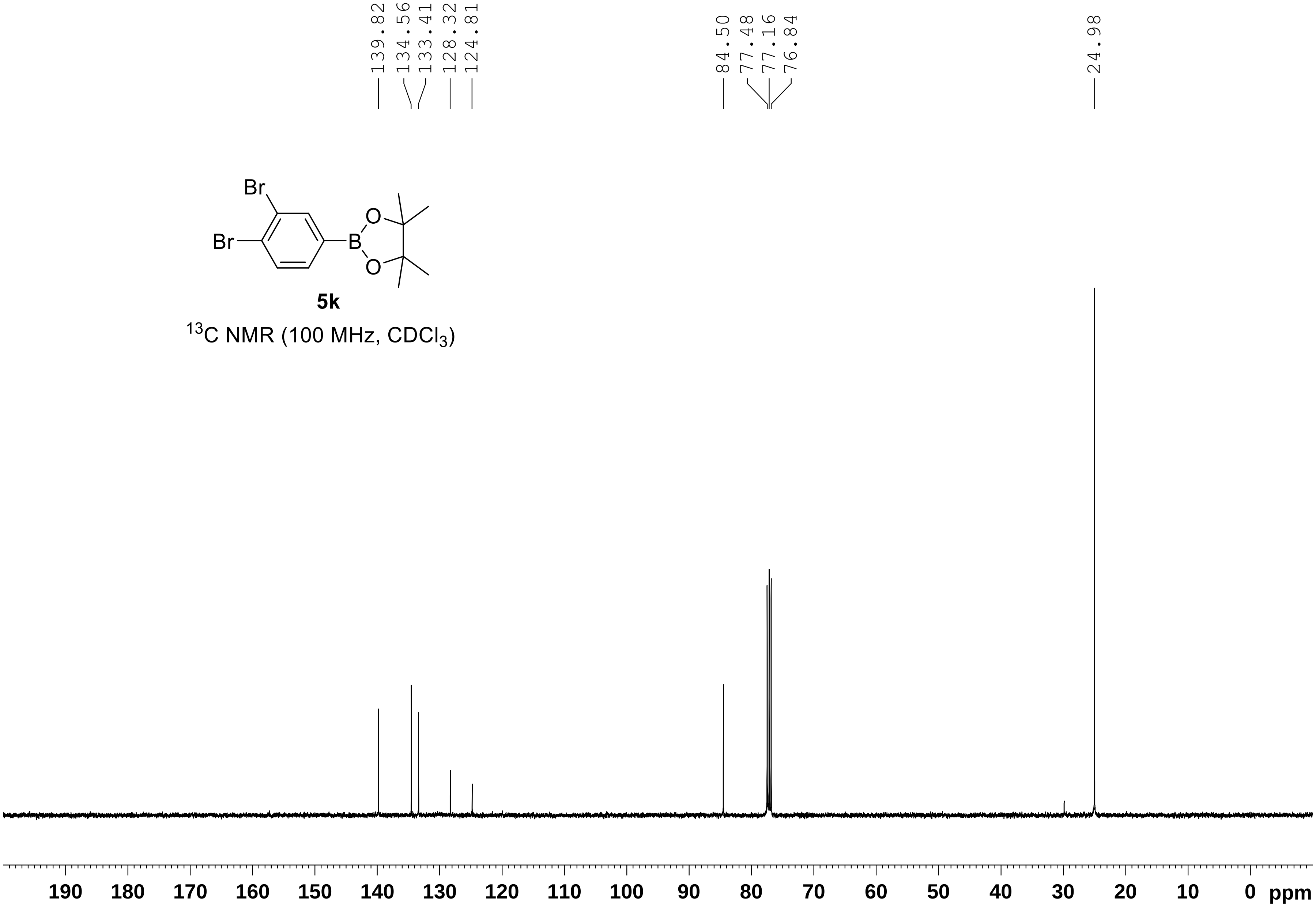

5k

^{13}C NMR (100 MHz, $CDCl_3$)

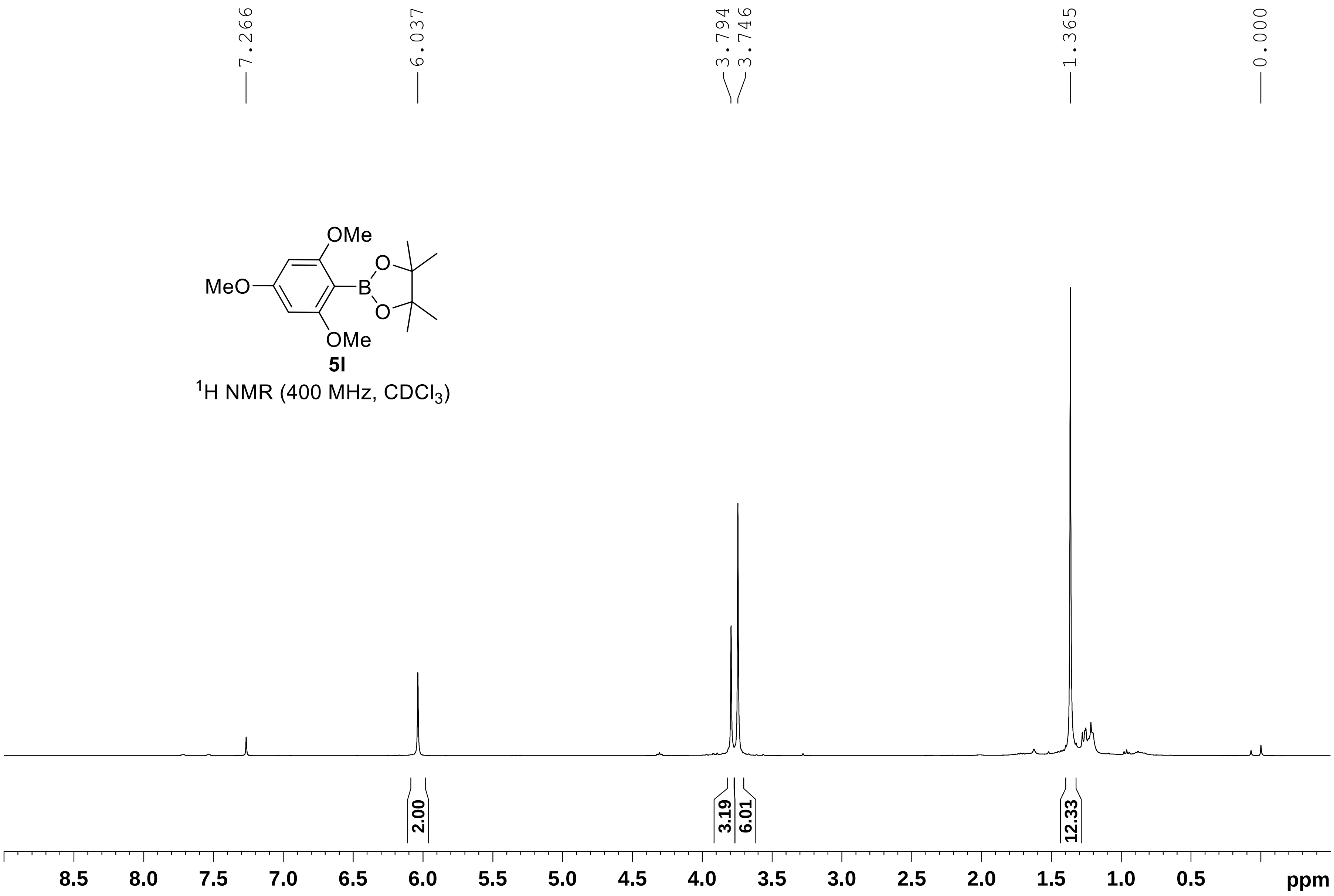
7.266
6.037
3.794
3.746
1.365
0.000
OMe
MeO
B
O
O
OMe
5l
^{1}H NMR (400 MHz, $CDCl_3$)
2.00
3.19
6.01
12.33
8.5
8.0
7.5
7.0
6.5
6.0
5.5
5.0
4.5
4.0
3.5
3.0
2.5
2.0
1.5
1.0
0.5
ppm

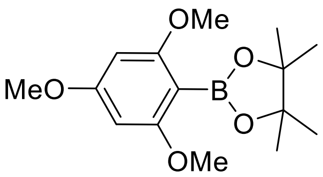

5l

^{13}C NMR (100 MHz, $CDCl_3$)

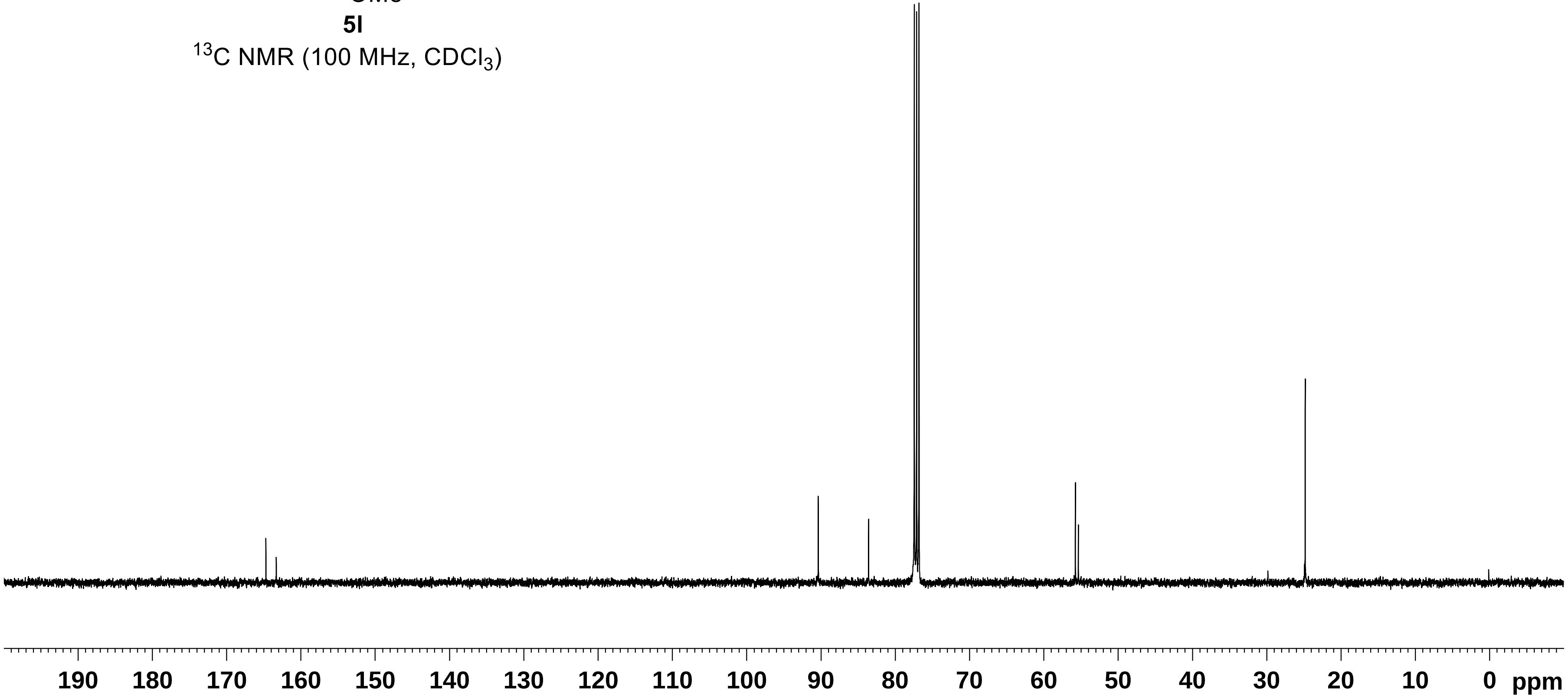

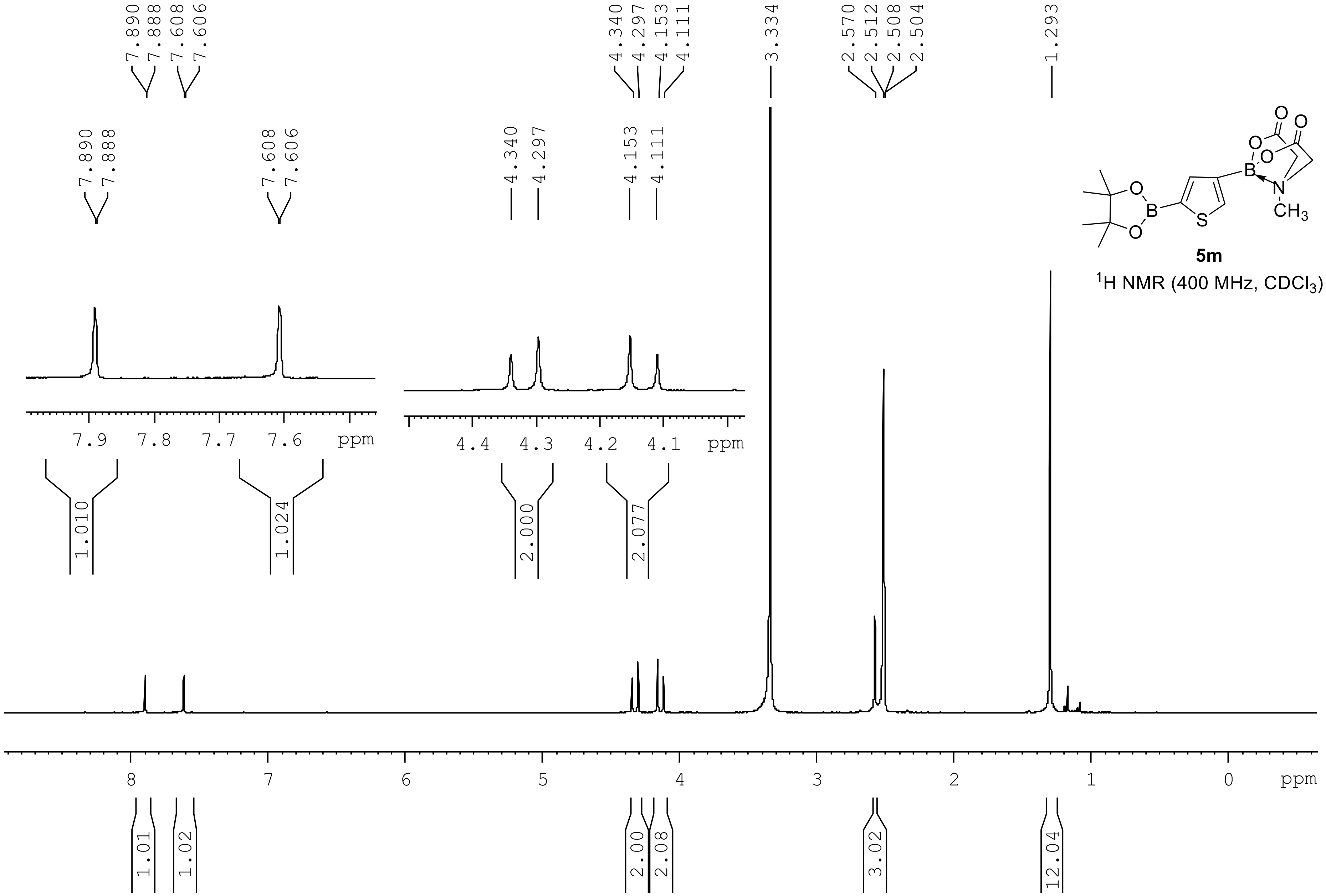

7.890
7.888
7.608
7.606
4.340
4.297
4.153
4.111
3.334
2.570
2.512
2.508
2.504
1.293
5m
^{1}H NMR (400 MHz, CDCl$_3$)
1.010
1.024
2.000
2.077
1.01
1.02
2.00
2.08
3.02
12.04
ppm

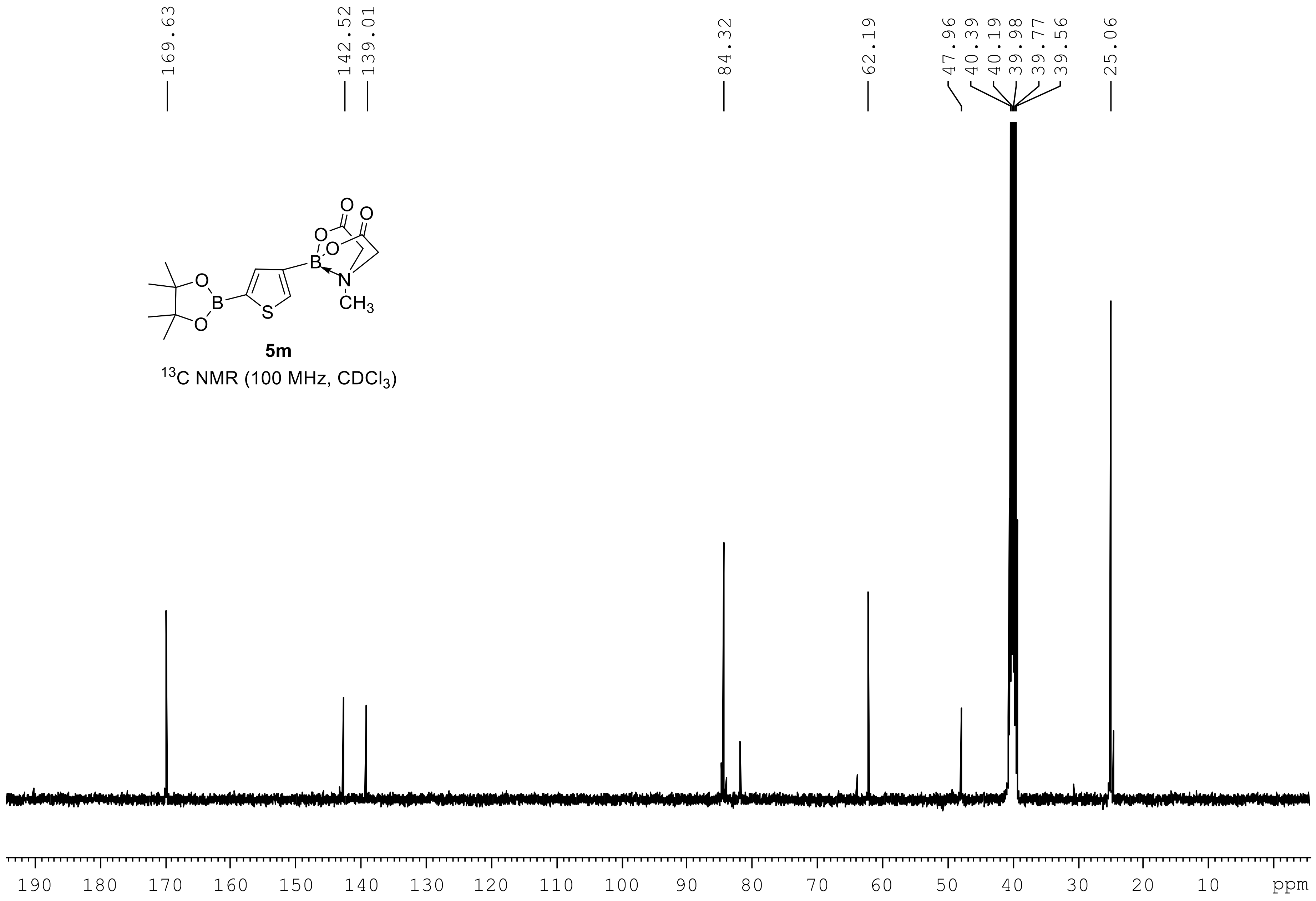
169.63
142.52
139.01
84.32
62.19
47.96
40.39
40.19
39.98
39.77
39.56
25.06
O
O
O
O
B
N
CH_3
S
B
O
O
5m
^{13}C NMR (100 MHz, $CDCl_3$)
190 180 170 160 150 140 130 120 110 100 90 80 70 60 50 40 30 20 10 ppm

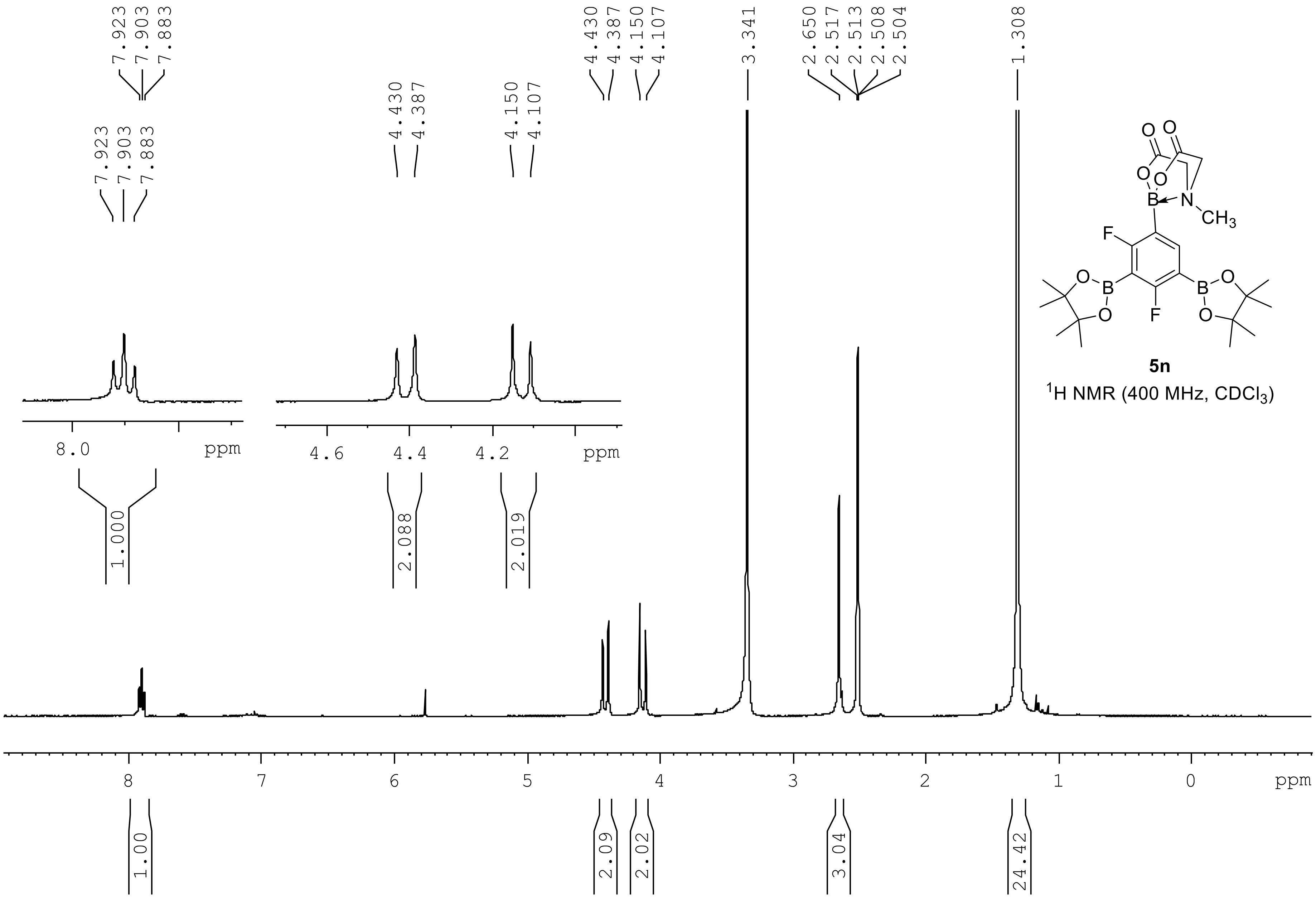

5n

^{1}H NMR (400 MHz, $CDCl_3$)

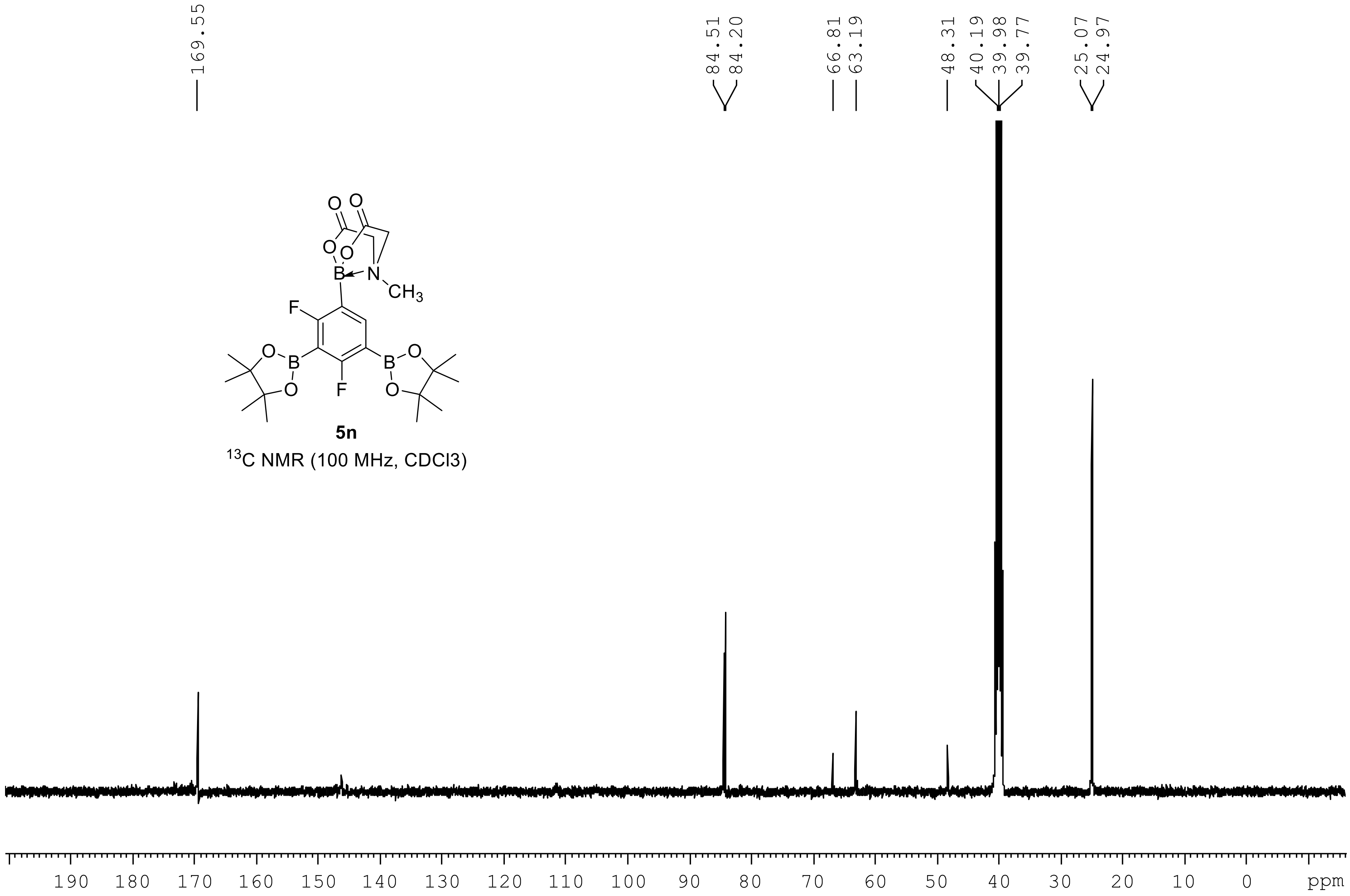
169.55
84.51
84.20
66.81
63.19
48.31
40.19
39.98
39.77
25.07
24.97
5n
^{13}C NMR (100 MHz, CDCl3)
190 180 170 160 150 140 130 120 110 100 90 80 70 60 50 40 30 20 10 0 ppm

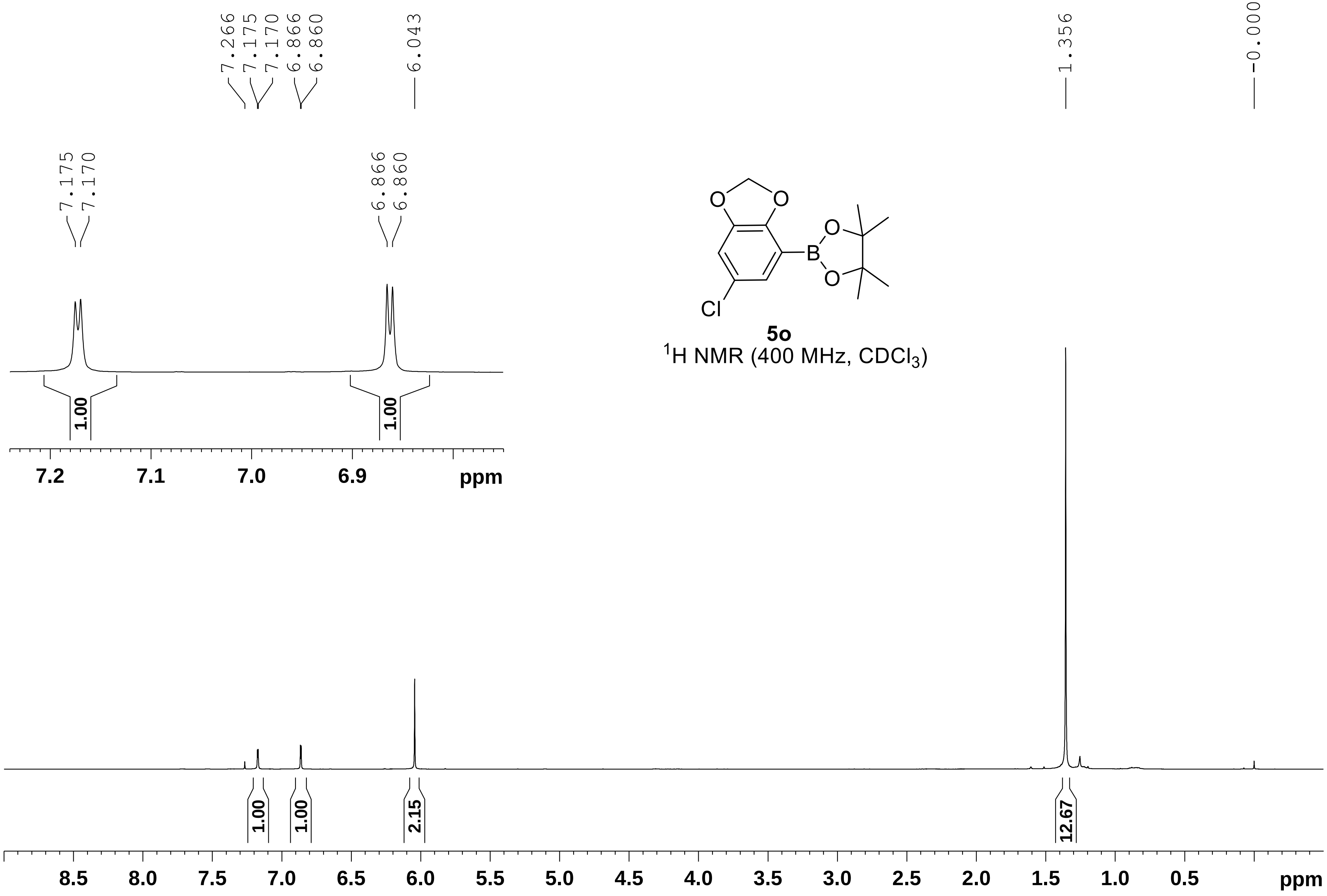

5o
^{1}H NMR (400 MHz, $CDCl_3$)

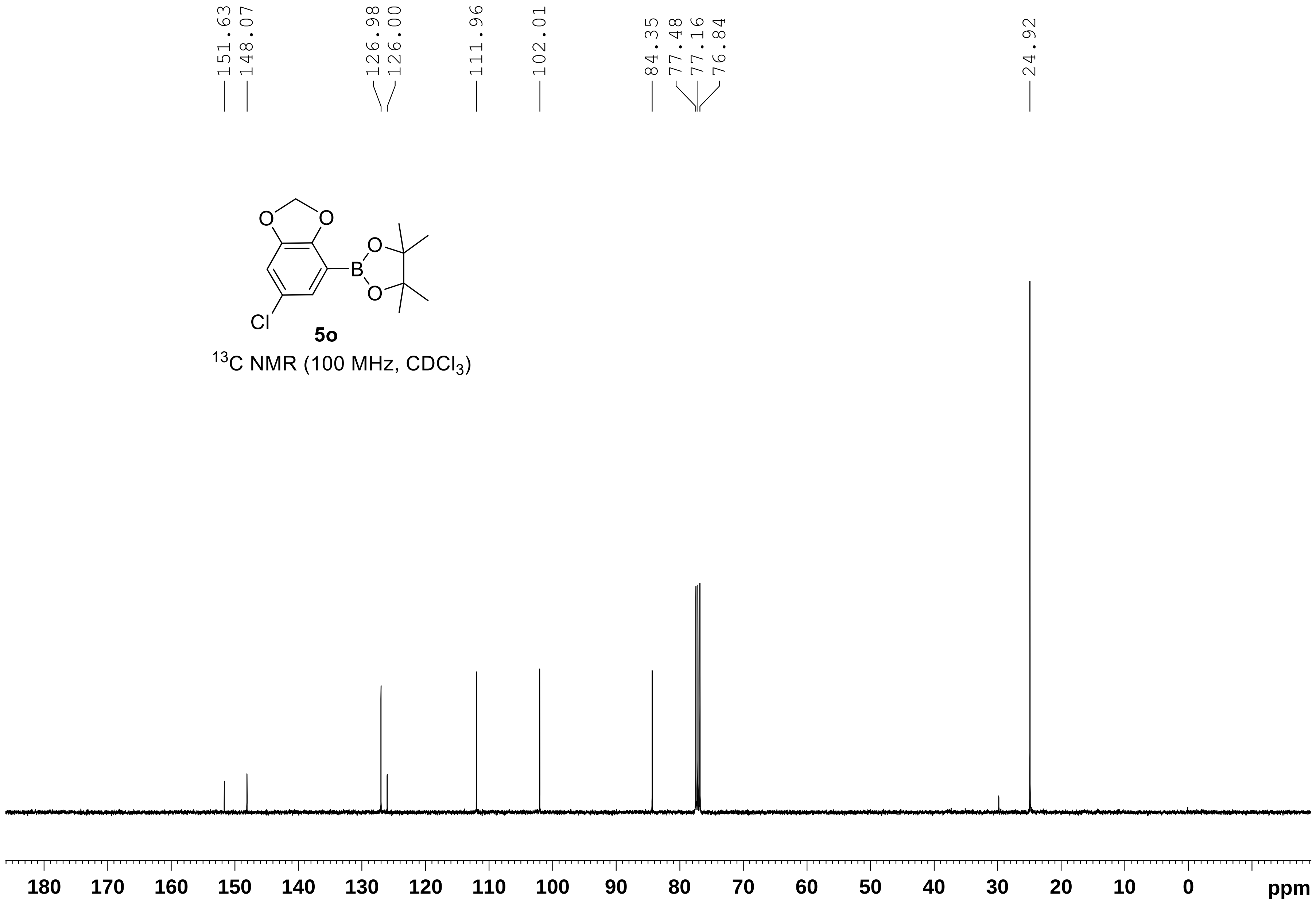
151.63
148.07
126.98
126.00
111.96
102.01
84.35
77.48
77.16
76.84
24.92
5o
^{13}C NMR (100 MHz, $CDCl_3$)
180 170 160 150 140 130 120 110 100 90 80 70 60 50 40 30 20 10 0 ppm

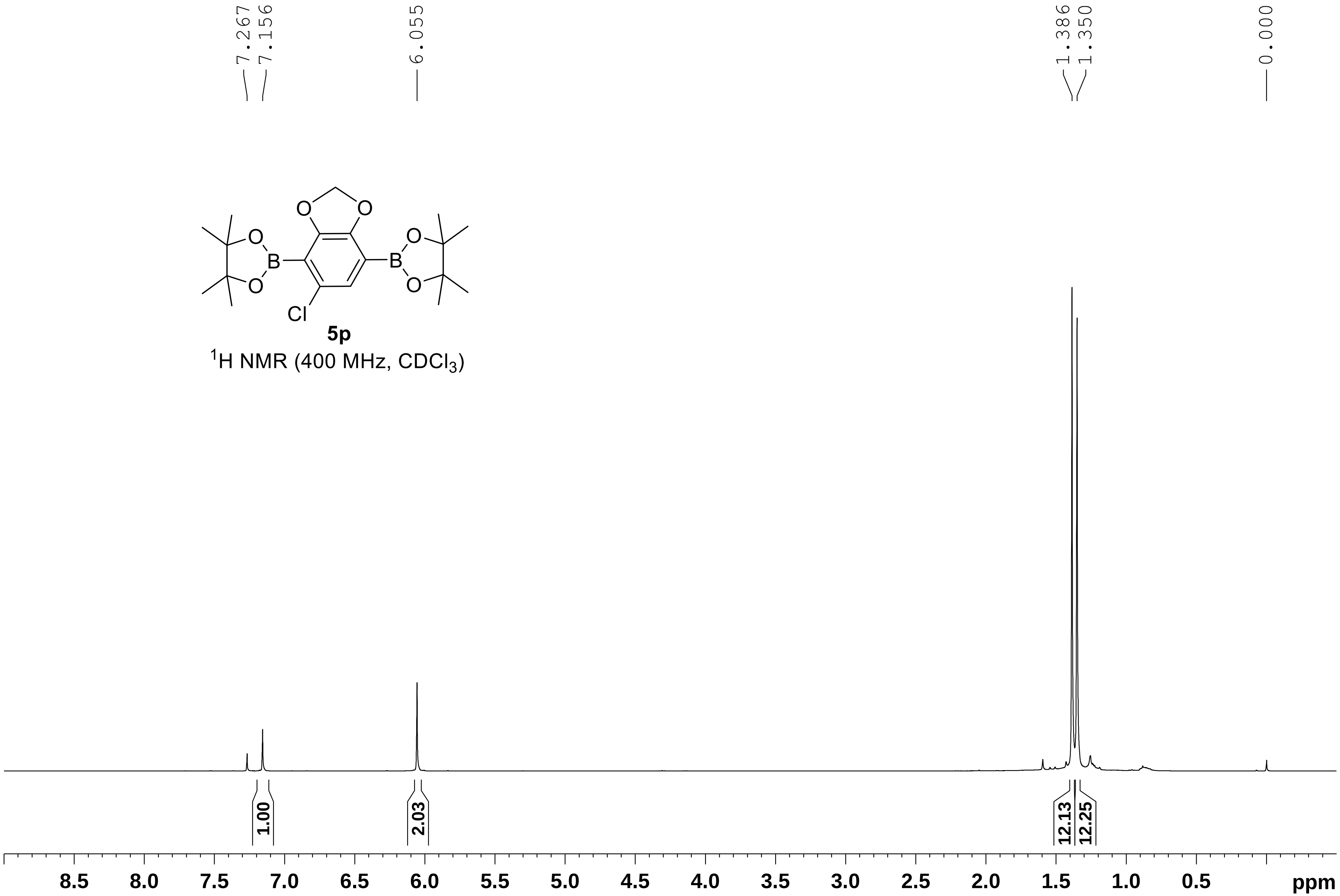
7.267
7.156
6.055
1.386
1.350
0.000
O
O
O
O
O
O
B
B
Cl
5p
^{1}H NMR (400 MHz, CDCl$_3$)
1.00
2.03
12.13
12.25
8.5
8.0
7.5
7.0
6.5
6.0
5.5
5.0
4.5
4.0
3.5
3.0
2.5
2.0
1.5
1.0
0.5
ppm

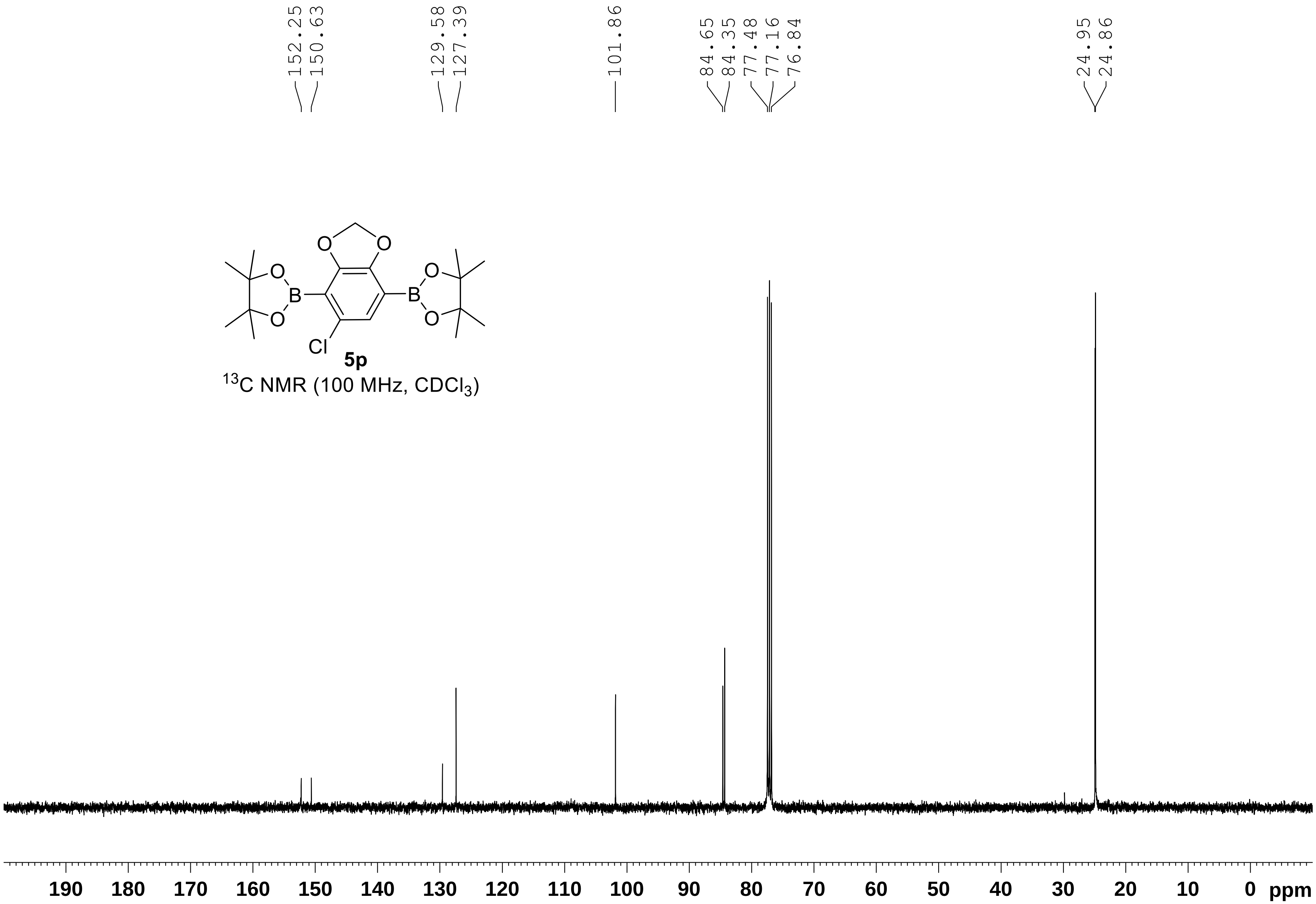
152.25
150.63
129.58
127.39
101.86
84.65
84.35
77.48
77.16
76.84
24.95
24.86
O
O
O
B
O
O
B
O
Cl
5p
^{13}C NMR (100 MHz, $CDCl_3$)
190 180 170 160 150 140 130 120 110 100 90 80 70 60 50 40 30 20 10 0 ppm
